# Permissive Transcriptional Activity at the Centromere through Pockets of DNA Hypomethylation

**DOI:** 10.1371/journal.pgen.0020017

**Published:** 2006-02-10

**Authors:** Nicholas C Wong, Lee H Wong, Julie M Quach, Paul Canham, Jeffrey M Craig, Jenny Z Song, Susan J Clark, K. H. Andy Choo

**Affiliations:** 1 Chromosome Research Laboratory, Murdoch Childrens Research Institute, Royal Children's Hospital, Parkville, Victoria, Australia; 2 Department of Paediatrics, University of Melbourne, Parkville, Victoria, Australia; 3 Epigenetics Group, the Garvan Institute of Medical Research, Darlinghurst, New South Wales, Australia; Babraham Institute, United Kingdom

## Abstract

DNA methylation is a hallmark of transcriptional silencing, yet transcription has been reported at the centromere. To address this apparent paradox, we employed a fully sequence-defined ectopic human centromere (or neocentromere) to investigate the relationship between DNA methylation and transcription. We used sodium bisulfite PCR and sequencing to determine the methylation status of 2,041 CpG dinucleotides distributed across a 6.76-Mbp chromosomal region containing a neocentromere. These CpG dinucleotides were associated with conventional and nonconventional CpG islands. We found an overall hypermethylation of the neocentric DNA at nonconventional CpG islands that we designated as CpG islets and CpG orphans. The observed hypermethylation was consistent with the presence of a presumed transcriptionally silent chromatin state at the neocentromere. Within this neocentric chromatin, specific sites of active transcription and the centromeric chromatin boundary are defined by DNA hypomethylation. Our data demonstrate, for the first time to our knowledge, a correlation between DNA methylation and centromere formation in mammals, and that transcription and “chromatin-boundary activity” are permissible at the centromere through the selective hypomethylation of pockets of sequences without compromising the overall silent chromatin state and function of the centromere.

## Synopsis

The centromere is a chromosomal structure that is vital for the correct partitioning of chromosomes during cell division. Recent studies in a number of different species have shown that transcription is permissible within the centromere, but the mode of transcription regulation at the centromere remains unclear. DNA methylation is a well-characterized mechanism for the genomic regulation of transcription. Here, the authors investigate the relationship between DNA methylation and transcription activity at a functional human centromere. They demonstrate a high level of DNA methylation across the centromere but identify pockets of DNA sequences within the methylated domain that are non-methylated. These pockets correspond to sites of transcription and/or boundaries that separate major centromeric chromatin sub-domains. This study shows the complexity of the centromere as it uses DNA methylation to both maintain a tight chromatin structure and to allow transcription to occur.

## Introduction

Centromeres are essential for the proper segregation of replicated chromosomes in eukaryotes. Recent studies have identified a plethora of conserved centromeric proteins [1,2]. Some of these proteins provide the foundation for the assembly and maintenance of a constitutive kinetochore, while others transiently associate with the centromere to confer specific roles such as sister chromatid cohesion, mitotic checkpoint function, and chromatid motor activity [3,4]. In addition, an increasing number of chromatin-modification proteins have been detected at the centromere and its surrounding heterochromatin [4–6]. Unlike the conservation of proteins at the centromere, centromeric DNA is highly divergent across species, suggesting an epigenetic influence on centromere identity and function [7–9].

DNA methylation is an important epigenetic marker that acts through the covalent addition of a methyl moiety to the cytosine residue of a CpG dinucleotide by the DNA methyltransferase (DNMT) family of proteins [10]. In humans, a CpG dinucleotide occurs at 5-fold less than the expected frequency [11]. It has been shown that the decrease in observed CpG frequency is complemented by an equal increase in observed TpG frequency, in part owing to the hypermutability of methylated cytosines to thymine [12,13]. However, there are pockets within the genome where the observed CpG frequency reaches the expected levels—suggesting that these regions may have a biological function. These regions are defined as CpG islands [14,15].

It has been estimated that up to 80% of all CpG dinucleotides in mammals are methylated [16]. CpG dinucleotides within CpG islands are more often than not unmethylated. The classical definition of these islands has been for sequences greater than 200 bp in length, with a GC content greater than 50% and an observed-to-expected GpC ratio of greater than or equal to 0.6 [15]. CpG islands are present in the promoter regions of approximately 40% of the genes in the mammalian genome [17,18]. Methylation of these CpG islands is thought to play a direct role in the control of gene transcription, genomic imprinting [19], X-chromosome inactivation [20], and in tumorigenesis [21].

The definition of a CpG island has recently been revised to include sequences greater than 500 bp in length, with a GC content greater than 55% and an observed-to-expected GC ratio greater than 0.65 [22]. These stringent measures enable the identification of functional CpG islands associated with the promoter region of genes and the exclusion of all parasitic GC-rich repetitive elements, generally thought to be hypermethylated as a means to protect the host [23].

The paucity of sequence markers at the highly repetitive centromeres presents a considerable challenge to the study of DNA methylation. However, studies in immunodeficiency, chromosome instability, and facial abnormalities (ICF) syndrome have given some insight into the role of DNA methylation in centromere stability. Patients with ICF syndrome carry a mutation in the DNMT3b gene and lose the ability to methylate DNA [24]. Owing to a substantial decrease in DNA methylation at both centromeric [25] and pericentric sequences [26], the chromosomes of these patients contain large regions of decondensed pericentromeric heterochromatin, suggesting that normal human centromeres are, overall, in a hypermethylated state. However, the repetitive-DNA nature of centromeres has precluded a detailed analysis of the pattern of methylation at the centromere.

Previously, we identified and characterized the phenomenon of ectopic human centromeres, known as neocentromeres [9,27,28]. Human neocentromeres form epigenetically at euchromatic chromosomal sites and are functionally and structurally similar to normal human centromeres [1,7,9]. These neocentromeres contain fully definable sequences and provide a tractable system for the molecular analysis of centromeric chromatin [29–31]. Using a neocentromere formed at band q25 of a Chromosome 10–derived neocentric marker chromosome mardel(10) (M10), we previously defined the relative positions of the constitutive centromeric proteins CENP-A and CENP-H, heterochromatin protein HP1α, enhanced chromosomal scaffold/matrix attachment regions (S/MAR), and delayed timing of centromeric DNA replication [30,32,33]. In addition, we previously showed that the transcriptional activity of genes within the modified neocentric chromatin is unaffected [32]. Here we take advantage of the fully known sequence, together with emerging knowledge regarding the chromatin organizational and transcriptional properties of the M10 neocentromere, to investigate the relationship between DNA methylation and chromatin-domain distribution and transcriptional competence at a functional human centromere.

## Results

We use the term CpG “islet” to describe a genomic region that is not classified as a CpG island because of its shorter length (<200 bp), but otherwise has a GC content and observed-to-expected CpG ratio that is characteristic of a CpG island. This study predominantly utilized two monochromosomal somatic cell hybrid lines, CHO–M10 and CHO–N10, which carried M10 or the normal paternal progenitor human Chromosome 10 (N10), from which M10 was derived [27,32]. To measure DNA methylation, we performed sodium bisulfite PCR and sequencing [34] on CpG islands and CpG islets selected from M10, and we compared their methylation pattern to the same islands and islets on N10. At least eight PCR amplicons were cloned and sequenced from M10 as well as from N10. The methylation level was expressed as a percentage of the number of CpG sites methylated to the total number of CpG sites assayed for each CpG island or islet.

**Figure 1:**
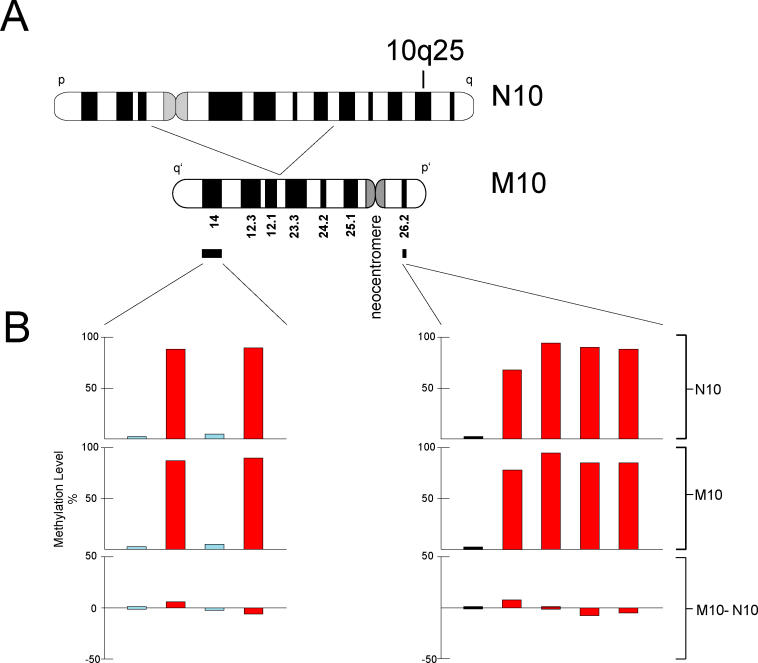
The 10q25 Locus and Control Regions Used in This Study (A) Chromosome ideogram representing the derivation of the M10 chromosome containing the neocentromere formed at the 10q25 band. CpG islands and CpG islets selected from the p14 and q26.2 bands situated 39 and 7 Mbp from the neocentric sites, respectively, were measured for methylation in both N10 and M10 cell lines. (B) The level of methylation is shown for the same CpG islands and CpG islets in N10 and M10, and for their differences (M10 minus N10). CpG islands of >500 bp are represented by black bars; CpG islands of >200 bp and <500 bp are represented by light-blue bars; and CpG islets are represented by red bars.

To ensure that any differences in DNA methylation between M10 and N10 were not an artifact of cell-line generation and maintenance [35], we used early-passage clonal lines of M10 and N10 for analysis. In addition, as controls, we used a number of CpG islands and islets from chromosomal bands 10p14 and 10q26.2, which were 39 Mbp and 7 Mbp, respectively, from the 10q25 neocentromere (Figure 1A). These were presumed to be sufficiently distant to be independent of the chromatin environment of the 10q25 neocentromere. The methylation level of these control CpG islands ranged from 0%–2% in both M10 and N10 (Figure 1B; Table S1). In N10, the methylation level of the control CpG islets ranged between 68% and 95%, while the same CpG islets in M10 ranged between 75% and 96%. Overall, the difference in methylation (M10 minus N10) ranged between −6% and +8%, reflecting background variation of our analytical system.

To determine the DNA-methylation profile and its relation to the chromatin environment of the neocentromere, we identified 129 GC-rich sequences within a 6.76-Mbp DNA region spanning the 10q25 neocentromere. In total, 2,041 individual CpG dinucleotides were assayed for DNA methylation, both before and after neocentromere formation (Tables S2 and S3). Throughout this study, methylation of cytosine at non-CpG sites was not observed.

### Methylation of CpG Islands

Across the 6.76-Mbp region, a total of 180 CpG islands were identified using the classical definition of a CpG island [15] (Table 1). Of the 180 islands, 78 were identified as being members of repetitive families. The number of CpG islands across this region was reduced to 20 using the modern CpG island definition [22]. This was in keeping with a total number of 50 annotated genes within this region. The more stringent criteria excluded all DNA repeats that were identified by the classical definition. For our methylation analysis, we selected a total of 22 CpG islands, of which six conformed to the modern criteria (Table 1).

**Table 1:**
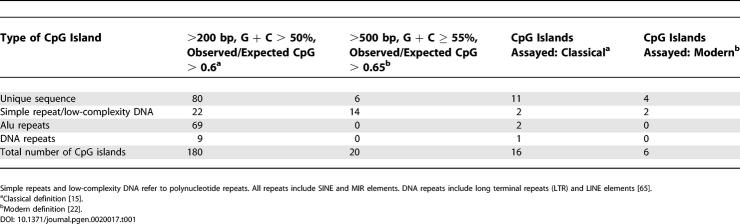
CpG Islands across the 6.76-Mbp 10q25 Region Analyzed

**Figure 2:**
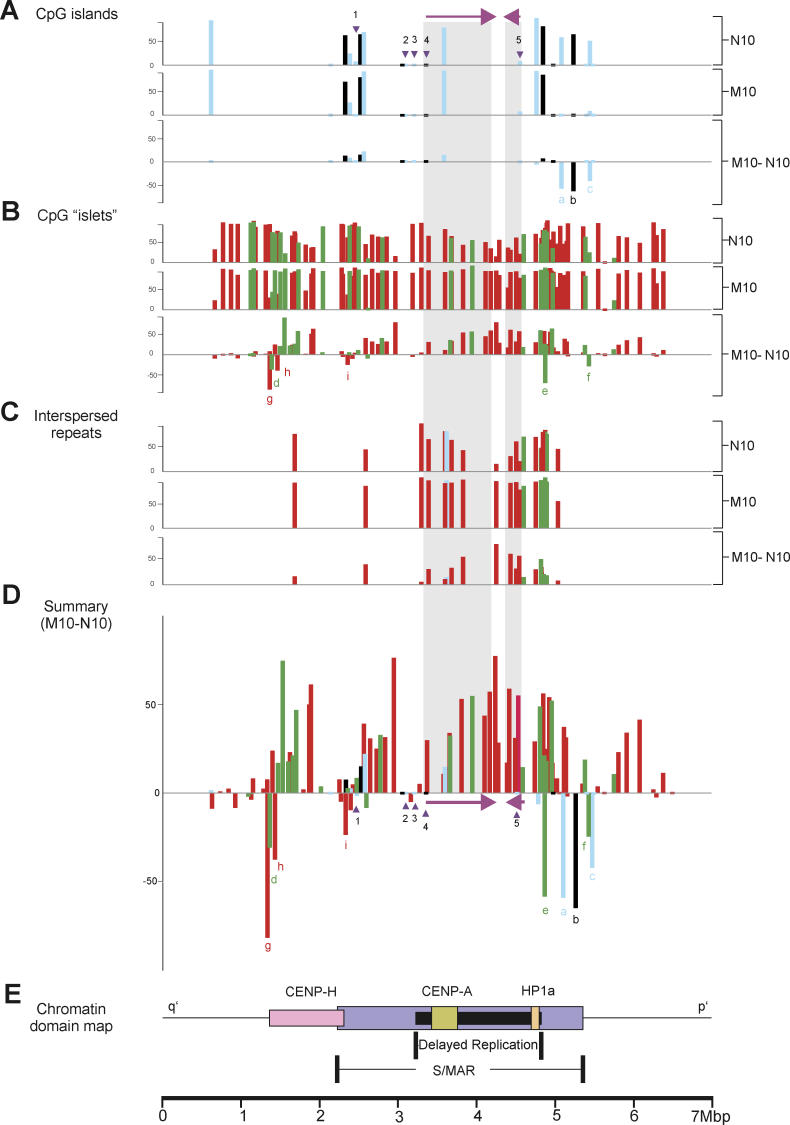
DNA-Methylation Profiles for 129 Separate CpG Islands and Islets Profiles apply across a 6.76-Mbp DNA section of the 10q25 region before (N10) and after (M10) neocentromere formation. The *y*-axis shows the percentage methylation in CHO–N10 and CHO–M10, and the percentage methylation difference (CHO–M10 minus CHO–N10). (A) Classically defined CpG islands [15] are represented by blue bars, while CpG islands conforming to the modern definition [22] are identified by black bars. Genes previously shown to be expressed in both CHO–N10 and CHO–M10 and known to be associated with a 5′ CpG island are labeled (1) FLJ10188, (2) KIAA1600, (3) TRUB1, (4) ATRNL1, and (5) GFRA1, with the position of their 5′-associated CpG islands indicated by inverted triangles, and the direction of transcription and the full extent of the two larger genes (4 and 5) indicated by the horizontal arrows and shaded areas, respectively. (B) CpG islets analyzed in this study with lengths ranging from 100 to 200 bp (green bars) or 50–99 bp (red bars). (C) Methylation levels of CpG islets identified as retrotransposable and other interspersed repetitive elements analyzed in this study. The full extent of the two larger genes (4 and 5) is indicated by the shaded areas. The length of CpG islets ranges from 100 to 200 bp (green bars) or 50–99 bp (red bars). (D) Summary of percentage methylation difference between CHO–M10 and CHO–N10. Three CpG islands and six CpG islets that are hypomethylated in M10 compared to N10 are denoted by a–i. (E) Relative positions of previously described chromatin domains corresponding to foundation centromeric proteins CENP-H and CENP-A, heterochromatin protein HP-1α, and enhanced S/MAR regions [32], and a region of delayed DNA replication [30].

CpG islands selected for DNA-methylation analysis ranged in size from 200 to 2,428 bp. Eleven of these CpG islands showed almost no detectable DNA methylation (0%–3% of sites methylated) for both N10 and M10 (Figure 2). Five of these islands were directly associated with the 5′ end of genes FLJ10188, KIAA1600, TRUB1, ATRNL1, and GFRA1 (labeled 1–5, respectively, in Figure 2) that we have previously shown to be expressed at equal levels on both N10 and M10 [32]. The remaining 11 islands showed detectable levels of DNA methylation on N10. Following neocentromere formation, the levels in five of these islands were largely unaltered, while three islands showed a 2%–15% increase in CpG methylation. Taken together, we observed an overall pre- and post-neocentromere DNA-methylation level mean of 34.3% and 37.1%, respectively—a difference of 2.8% (*p* = 0.113, paired *t*-test)—demonstrating that the levels of methylation of these CpG islands were not significantly different between N10 and M10.

In contrast, the methylation levels for the final three islands (labeled a–c in Figure 2A and 2D) were significantly reduced from a range of 51.3%–65.2% in N10 to a range of 0%–0.4% in M10 (*p* < 0.0001, paired *t*-test). These three islands were positioned towards the p′ outer boundary region of the previously mapped HP1α-binding and S/MAR–enriched chromatin [32] (Figure 2E).

### Methylation of CpG Islets

The failure to detect a significant overall methylation difference within the neocentromere core region, owing to the conventionally defined CpG islands, prompted us to examine DNA sequences that we have designated as CpG islets. We analyzed a total of 107 CpG islets (Figure 2B). A small number of CpG islets (4/107) showed a low level of methylation that was not detectably altered in N10 and M10. Of the remaining CpG islets, 97 had detectable underlying DNA methylation in N10, with the majority (84/97) showing increased DNA methylation to saturating levels in M10 (Figure 2B). Twelve CpG islets showing increased methylation were located within the introns of expressed genes ATRNL1 (eight islets) and GFRA1 (four islets) (genes 4 and 5, Figure 2B and 2D). Overall, the level of CpG islet-related DNA methylation within the neocentromere increased from a pre-neocentromere mean of 61% (in N10) to a significantly higher post-neocentromere value of 83% (in M10)—a difference of 22% (*p* < 0.0001, paired *t*-test).

Not all differentially modified CpG islets showed an increased methylation pattern. Six islets, labeled d–i in Figure 2B and 2D, showed a substantial decrease in methylation level from 64.9% in N10 to 35.9% in M10 (a difference of −29.1%, *p* < 0.002, paired *t*-test). As with the three hypomethylated CpG islands, these hypomethylated CpG islets resided towards the outer boundaries of the S/MAR enrichment—CENP-H- and HP1α-binding domains within the neocentromere (Figure 2E).

### Methylation of Interspersed Repeats

Our analysis also investigated the level of DNA methylation within 25 sequences that were comprised of retrotransposable or other repetitive elements, including LINEs (long interspersed nuclear elements), SINEs (short interspersed nuclear elements), and MIRs (mammalian interspersed repeats) (Figure 2C) (see Tables S2 and S3 for detailed locations and repeat subtypes). These elements showed variable levels of DNA methylation on N10; however, upon neocentromere formation, these levels increased to saturation within the M10 neocentric chromatin (Figure 2C). Four islets (and one island) within the introns of ATRNL1 (gene 4, Figure 2A and 2D) and three islets within those of GFRA1 (gene 5, Figure 2A and 2D) belonged to these classes of interspersed repeats.

### Methylation of CpG-Orphan Sites

The observed differences in methylation of CpG islets prompted us to further assess DNA methylation at CpG dinucleotides within DNA sequences that were GC-poor (<40%) and without the expected frequency of CpG dinucleotides. As such, these sequences were not classified as either CpG islands or CpG islets. The CpG dinucleotides found in these sequences, which we have termed orphaned CpGs, are thought to be methylated as they reside outside the realm of CpG islands [14].

**Figure 3:**
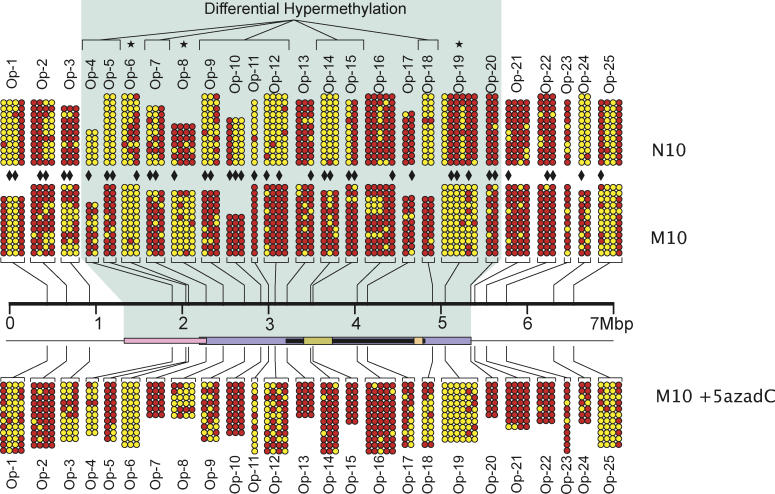
Methylation Analysis of CpG Orphans A total of 75 CpG orphans were selected from 25 sites (Op-1 to Op-25). Red circles denote methylated CpG dinucleotides, and yellow circles denote unmethylated CpG dinucleotides. The positions of Op-1 to Op-25 sites with respect to the previously defined centromeric chromatin domains (shaded area) are indicated (refer to Figure 2E for details). Sites showing differential hypermethylation following neocentromere formation are labeled on top. Sites showing differential hypomethylation are denoted by asterisks. CpG dinucleotides that are putative MeCP2-binding sites are indicated by solid diamonds. Upon treatment with 5-aza-dC, a number of CpG orphans (Op-4, Op-9, Op-11, Op-12, Op-17, and Op-18) demonstrated a significant decrease in DNA methylation.

We selected 25 regions encompassing 75 CpG orphans across the 6.76-Mbp neocentromere domain for bisulfite PCR and sequencing (Figure 3; Table S4). Our results showed that methylation at orphan CpG sites in N10 and M10 were highly variable. However, within the neocentromere region, a significant number of orphan-containing regions (Ops) (Op-4, Op-5, Op-7, Op-9, Op-10, Op-11, Op-12, Op-14, Op-15, and Op-18; Figure 3) showed increased methylation from a pre-neocentromere (N10) mean of 46% to a post-neocentromere (M10) mean of 78%—a difference of 32% (*p* < 0.0001, paired *t*-test). In addition, we observed hypomethylation in a small number of Ops (Op-6, Op-8, and Op-19; Figure 3) in M10 compared to N10 that were again located near the outer boundaries of previously defined neocentric chromatin domains. These findings therefore provided corroborating evidence for the methylation trends observed with the CpG islets following neocentromere formation in M10.

### Maintenance of DNA-Methylation Profile at the Neocentromere in Different Cell Lines

**Figure 4:**
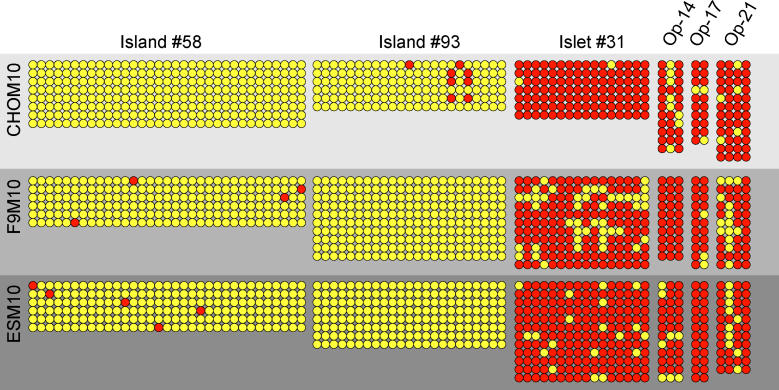
Determination of Possible Variation in DNA-Methylation Pattern owing to Cell-Line Differences The neocentromere-containing M10 chromosome was initially studied in a CHO hybrid cell background but was transferred to a mouse F9 and mouse ES cell line for further testing. DNA methylation was assessed on a selected number of CpG island, islets, and orphans that showed different DNA-methylation levels in the initial assay in CHO. The results indicated no significant difference in methylation levels in all three cell backgrounds.

In order to address the concern that the DNA-methylation profile we observed might be cell line–specific, we transferred the human M10 chromosome into mouse F9 cells and mouse ES cells, and compared the DNA-methylation level of two CpG islands, a CpG islet, and three CpG orphans with those of M10 in the Chinese hamster ovary (CHO) background. CpG islands 58 and 93 were hypomethylated in M10 in the CHO background and remained hypomethylated in the mouse F9 and ES somatic cell hybrid lines (Figure 4). CpG islet 31 and orphans 14, 17, and 21 were hypermethylated in M10 in CHO and remained hypermethylated in both mouse backgrounds. These data indicated that the observed DNA-methylation patterns were not due to cell-line differences.

### Non Strand-Specificity of DNA Methylation at the Neocentromere

**Figure 5:**
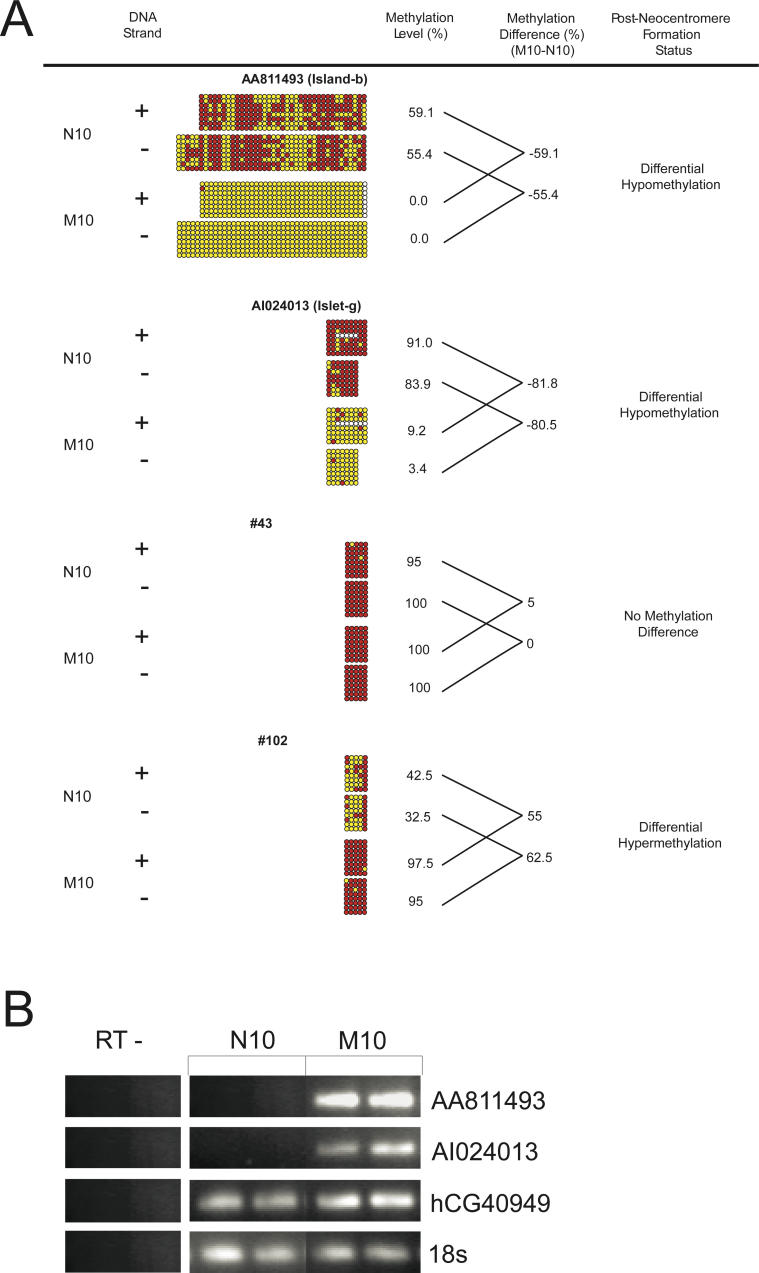
DNA Strand-Bias Analysis Four sequences were chosen for this analysis. (A) The EST-associated CpG island (AA811493; island b in Figure 2A and 2D), the EST-associated CpG islet (AI024013; islet g in Figure 2B and 2D), and two additional islets designated as numbers 43 and 102, respectively (see Tables S2, S3, and S5). Individual CpG dinucleotides are shown as columns. Red and yellow circles denote a methylated and unmethylated CpG dinucleotide, respectively. A very small number of undetermined sites are indicated by white circles. The aligned columns represent the same CpG dinucleotide from both strands. The methylation level was calculated as described in Materials and Methods. (B) RT-PCR analysis of an EST-associated CpG island (AA811493) and islet (AI024013). Positive controls were the 18S RNA standard and TRUB1 (corresponding to gene 3 in Figure 2A), which has previously been shown to be expressed in both CHO–N10 and CHO–M10 [32]. RT− indicates control lanes showing no DNA contamination from RNA prepared for this analysis.

As the nature of bisulfite PCR and sequencing is strand-specific, we tested the possibility of a strand-specific bias in DNA methylation. We assayed methylation of the complementary (−) DNA strand on one CpG island and of three CpG islets that represented either no methylation difference, or differential hyper- or hypomethylation between M10 and N10 (Figure 5). These sequences corresponded to the unspliced expressed sequence tags (ESTs) AA811493 and AI024013 (island b and islet g, respectively, Figure 2D) and two randomly chosen islets (listed as islets 43 and 102 in Tables S2, S3, and S5). The results indicated that, in all cases, the overall methylation level for the positive strand was closely comparable to that of the negative DNA strand (Figure 5A), suggesting the absence of any strand-specific bias in DNA-methylation profile at the 10q25 neocentromere.

### EST Expression of Differentially Hypomethylated CpG Islets

Of the nine CpG islands/islets that showed significant DNA hypomethylation, two corresponded to the unspliced ESTs AA811493 and AI024013 (island b and islet g, respectively, Figure 2D), where the DNA-methylation levels decreased from 59.1% in N10 to 0% in M10 for AA811493, and from 91% in N10 to 9.2% in M10 for AI024013 (Figure 5A). Analysis of the transcription status of these two ESTs before and after neocentromere formation using RT-PCR indicated no basal transcription from both loci in N10, but did indicate clear transcription in M10 (Figure 5B). The gene TRUB1 (gene 3 in Figure 2A, shown to be expressed in both N10 and M10 previously [32]), was used along with 18S as a positive-expression control. The detection of transcriptional activity was therefore in keeping with the demethylated state of these two EST-associated CpG island/islets in M10 (Figure 5A).

### Reduction of DNA-Methylation Level Using 5-aza-Deoxycytidine

**Figure 6:**
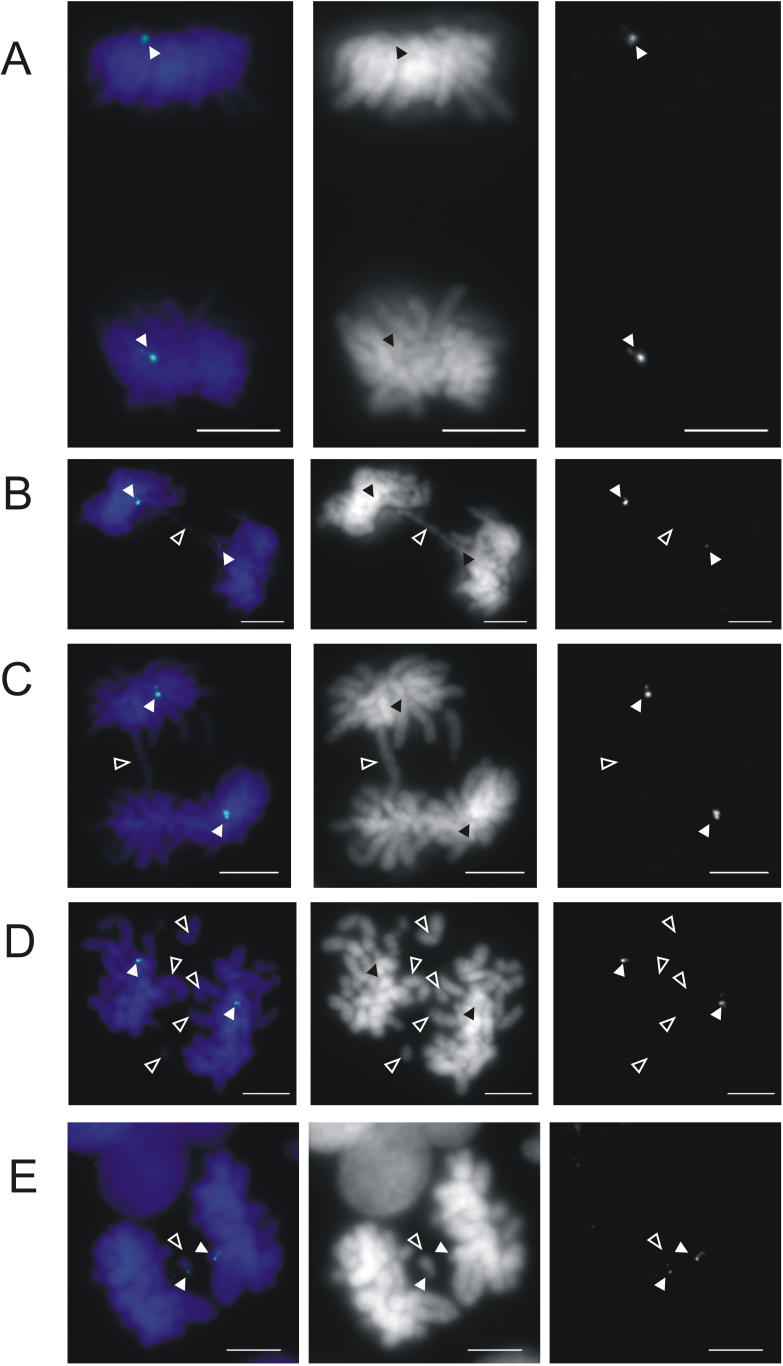
Anaphase Analysis of CHO–M10 Cells were treated with 20 μM 5-aza-dC for a period of 3 d. (A) An untreated control cell undergoing normal anaphase, hybridized with M10-specific FISH probe to show correctly segregated M10 neocentromere (blue-green, filled arrowheads). (B and C) 5-aza-dC–treated anaphase cells showing lagging or bridging chromosome (open arrowhead) and the location of the correctly segregated M10 neocentromeres (green, filled arrowheads). (D) Anaphase cell with multiple lagging chromosomes (open arrowheads) and correctly segregated M10 (green, filled arrowheads). (E) Anaphase cell with a lagging M10 chromosome (open arrowhead). The M10 neocentromere probe is shown in green (filled arrowhead). Scale bar denotes 5 μm.

**Figure 7:**
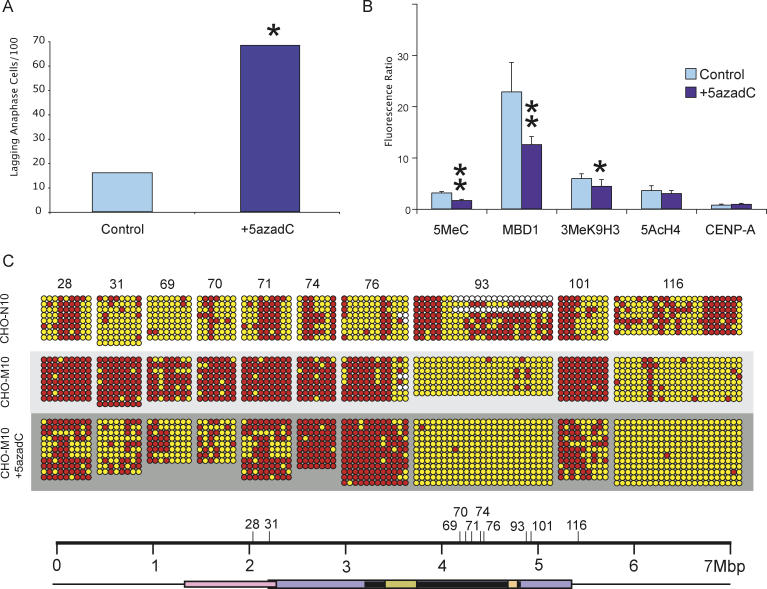
Effects of 20 μM 5-aza-dC Treatment on the 10q25 Neocentromere (A) Inhibition of DNA methylation with 5-aza-dC increases centromere instability as demonstrated by a significant increase in the proportion of anaphase cells containing lagging or bridging chromosomes when compared with non-drug-treated PBS control Asterisk indicates *p* < 0.001, χ^2^-test. (B) Fluorescence-intensity ratio of chromatin proteins at the M10 neocentromere before and after 5-aza-dC treatment. The amount of 5MeC and MBD1, indicators of DNA methylation, was significantly reduced at the 10q25 neocentromere after 5-aza-dC. The histone modification, 3MeK9H3, associated with methylated DNA, also showed a significant reduction after 5-aza-dC treatment. Penta-acetylated H4, a histone modification associated with euchromatin, and constitutive centromeric protein A (CENP-A) were not significantly altered after 5-aza-dC treatment (mean raw fluorescence values are presented in Table S6). Single asterisk denotes *p* = 0.014, and double asterisks denote *p* < 0.001 in a Fisher's Exact *t*-test, with error bars representing standard errors of the mean. (C) Determination of DNA-methylation level of eight CpG islets and two islands at the 10q25 neocentromere following 5-aza-dC treatment. Six CpG islets (28, 31, 69, 70, 71, and 101) showed an increase in DNA methylation upon the formation of the 10q25 neocentromere. However, following 5-aza-dC treatment, the methylation level of these CpG islets decreased significantly. The locations of the eight CpG islets and two CpG islands (numbers 93 and 116) with respect to the mapped centromere domains are depicted on the scale bar. The numbers for the CpG islands and islets correspond to those shown in Tables S2, S3, and S5.

To ascertain whether CpG methylation is important for the functional integrity of the neocentromere, we used a DNA methylation–inhibiting drug, 5-aza-deoxycytidine (5-aza-dC), to reduce methylation at the neocentromere. Centromere instability was measured by the appearance of lagging or bridging chromosomes at anaphase (Figure 6). The cells were treated with 20 μM 5-aza-dC over a period of 3 d, followed by synchronization with nocadazole and release in the presence of cytochalasin B (see Materials and Methods). This treatment inhibits cytokinesis and can enhance the retention of cells containing missegregated chromosomes. Following treatment, the proportion of anaphase cells containing bridging (Figure 6B) or lagging (Figure 6C and 6D) chromosomes increased from 19% in control PBS-treated cells to 69% in cells treated with 5-aza-dC (Figure 7A).

No lagging or bridging M10 chromosomes were identified amongst 150 PBS-treated anaphase cells examined. However, when treated with 5-aza-dC, lagging M10 chromosomes were found in 6% (4/65) of anaphase cells (Figure 6E), suggesting that disruption of DNA methylation at the M10 neocentromere perturbed centromere function, causing chromosomal instability and missegregation.

### Immunofluorescence Studies

To investigate whether 5-aza-dC affected chromatin protein binding, we measured the signal intensity of antibodies raised against three different heterochromatin markers: 5-methylated cytosine (5MeC), methylated DNA-binding domain (MBD) protein 1, and histone H3 (tri-methylated lysine 9), at the M10 neocentromere. In addition, we included antibodies against penta-acetyl histone H4 (a marker for euchromatin) and the constitutive centromeric histone H3 variant, CENP-A. Human CREST-3 antiserum [27] recognizes all human centromeres and was used to co-detect with the various antibodies.

Three independent experiments were performed using 20 μM 5-aza-dC. From each experiment, 20 metaphase chromosome spreads were digitally captured for fluorescence-intensity measurement at the M10 neocentromere (Table S6). The CREST-3 signal was found to be consistent on the M10 neocentromere before and after 5-aza-dC treatment within all three experiments (data not shown), and was used for signal normalization to calculate a fluorescence-intensity ratio. The fluorescence-intensity ratio was calculated for each test antibody to the CREST-3 signal at the neocentromere (Table S7). The mean fluorescence-intensity ratio was used to compare 5-aza-dC–treated cells to untreated cells. A significant decrease in fluorescence-signal ratio was observed for 5MeC after 5-aza-dC treatment (1.5; *p* < 0.00001; paired *t*-test) (Figure 7B), indicating that DNA methylation at the neocentromere was perturbed. In line with this, significant reductions in fluorescence-signal ratios were seen in the heterochromatin markers MBD1 (10.2; *p* = 0.0003; paired *t*-test) and 3MeK9H3 (1.5; *p* = 0.014; paired *t*-test). No significant change was observed with CENP-A (0.1; *p* = 0.21) and the euchromatin marker, penta-acetyl histone H4 (0.5; *p* = 0.14) (Figure 7B).

### CpG Methylation Following 5-aza-dC Treatment

We selected two CpG islands and eight CpG islets from Figure 2 for bisulfite PCR and sequencing after 5-aza-dC treatment. The CpG islets (numbered 28, 31, 69, 70, 71, 74, 76, and 101, Figure 7C) showed a significant increase in methylation in M10 when compared to N10 before 5-aza-dC treatment. Amongst these CpG islets, six (numbered 28, 31, 69, 70, 71, and 101) showed a significant reduction in methylation (with the reduction ranging between 14.5% and 68.1%) after 5-aza-dC treatment. In comparison, methylation of the remaining two CpG islets in this group (numbered 74 and 76, Figure 7C) was not altered significantly after 5-aza-dC treatment.

CpG islands 93 and 116 showed hypomethylation in M10 compared to N10 before 5-aza-dC treatment, and remained hypomethylated after addition of 5-aza-dC (Figure 7C).

In addition, we used bisulfite sequencing analysis to determine the methylation status of the CpG dinucleotides within the 25 sites containing CpG orphans following 5-aza-dC treatment (Figure 3). Of the ten sites (Op-4, Op-5, Op-7, Op-9, Op-10, Op-11, Op-12, Op-14, Op-15, and Op-18) that were hypermethylated in M10 before drug treatment, five (Op-4, Op-9, Op-11, Op-12, and Op-18) showed a significant reduction in methylation level in one or more CpG dinucleotides (Figure 3). No significant change was seen in M10 for the remaining orphans before and after drug treatment.

### Distribution of Putative MeCP2 Sites at the 10q25 Neocentromere

In the light of a recent study identifying the binding-sequence requirement of the MBD protein MeCP2 [36], we examined the distribution of putative MeCP2-binding sites in all the CpG islands, CpG islets, and CpG orphans analyzed in this study. A large proportion of CpG dinucleotides in the CpG-orphan regions (49.3% of CpGs analyzed) were found to be putative MeCP2-binding sites (Table 2 and Figure 3). In contrast, the proportions of putative MeCP2-binding sites associated with the CpG islands and CpG islets were significantly lower (4.7% and 10.7%, respectively, Table 2). These findings suggest that the CpG-orphan regions may provide a potentially very rich genomic repertoire for MeCP2 binding and its associated activities.

**Table 2:**
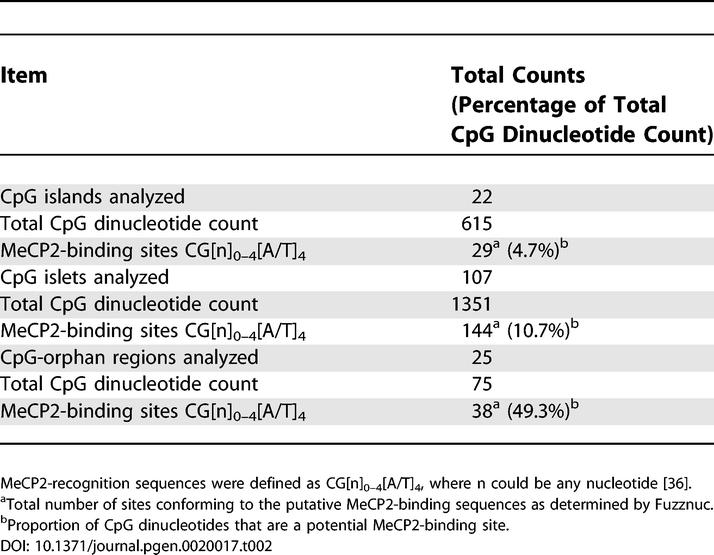
Relative Distribution of Potential MeCP2-Binding Sites Flanking the CpG Islands, CpG Islets, and CpG Orphans Analyzed in This Study

## Discussion

Bisulfite PCR and sequencing provides a sensitive technique to measure DNA methylation quantitatively at every CpG dinucleotide within a genomic region of interest [34]. Here, we used this technique to study DNA methylation over a 6.76-Mbp DNA region at the 10q25 locus of the normal human chromosome N10 and the M10 derivative.

As a control, we measured DNA methylation of seven sequences that are positioned at a considerable distance (>7 Mbp) from the neocentromere domain (Figure 1) to determine whether the process of generating the N10 and M10 CHO cell lines had affected DNA methylation. No significant difference was observed in the methylation of these sequences between N10 and M10, suggesting that the generation of these cell lines has not altered the pattern of DNA methylation. Furthermore, we directly compared the DNA-methylation levels of a number of CpG islands, islets, and orphans within the M10 neocentromere in CHO and two mouse cell lines. We found no significant differences, indicating that the DNA-methylation patterns we observed in this study were not caused by cell-line differences. We also investigated the phenomenon of strand-biased methylation previously reported in *Arabidopsis* centromeres [37]. Our results indicate no significant strand-biased methylation in any of the CpG island/islet examples assayed at the neocentromere. These results are consistent with the observed activity of the vertebrate housekeeping DNMT1 that converts hemi-methylated CpG dinucleotides to a fully methylated state [10]. A previous study in plants also demonstrated methylation of the cytosine residue of CpNpG trinucleotides by chromomethylase [38]. To date, such methyltransferases have not been identified in vertebrates [10], and this is consistent with the fact that no methylation of CpNpG trinucleotides (or any other non-CpG sites) was observed throughout our study.

In all, we analyzed the methylation status of 2,041 CpG dinucleotides distributed amongst CpG-island, -islet, and -orphan sequences within the 10q25 region. We found a substantial increase in the overall level of DNA methylation at the neocentromere compared to its pre-centromere state at normal 10q25. It is not possible to extrapolate from these results whether increased DNA methylation is directly responsible for the epigenetic formation of a functional neocentromere, as it is possible that the observed changes in DNA methylation occur subsequent to neocentromere seeding. However, the observed increase in DNA methylation at the neocentromere could provide the basis for the epigenetic identity and maintenance of the centromere [4]. Other studies have shown, for example, that while chromatin remodeling involving histone modifications precedes the silencing of genes during mammalian X-inactivation, the inactive status is imprinted and maintained through DNA methylation (reviewed in [39] and in references listed therein).

DNA hypermethylation at the neocentromere is shown to occur throughout the previously mapped functional chromatin domains of enhanced S/MAR, and CENP-H, CENP-A, and HP1α-binding [32]. Our data demonstrate that this hypermethylation is attributed primarily to the systemic methylation of the CpG islets and CpG orphans, but not the conventional CpG islands. Several studies have shown that CpG methylation promotes the recruitment of silencing or heterochromatin protein complexes and the assembly of a compacted chromatin state [40–42]. A number of such protein complexes, including the methylated CpG-binding proteins MBD1, MBD2, and MeCP2, plus DNMT1, DNMT3b, and heterochromatin protein HP1α, have been observed on the M10 neocentromere [5]. It is possible that the hypermethylation seen at the neocentromere serves to recruit these protein complexes to impart and maintain an overall compact centromeric chromatin state.

Our analysis showed that centromeric DNA is not uniformly hypermethylated. We identified at least two situations in which hypomethylation of pockets of centromeric DNA occurs. Firstly, we observed a distinct clustering of hypomethylated sites at the p′ and q′ outermost regions of the mapped S/MAR–enhanced and CENP-H–binding domains. Two discernible sites of hypomethylation also coincide with the HP1α-binding domain and the inner boundary of the CENP-H-binding (or S/MAR–enhanced) domain. It is possible that DNA hypomethylation marks the site of transition between two different centromeric chromatin domains, such as through the recruitment of insulator proteins to demarcate and protect a chromatin domain from the disruptive spreading of dissimilar neighboring chromatin states [43]. Our demonstration of transcriptional activity at two of the hypomethylated boundary sites (island b and islet g) further suggests that hypomethylation of the boundary regions may be important for the transcription of centromeric DNA in these regions (see below), and that CpG islets, like CpG islands, could play a role in the expression-control of genes and ESTs.

The second situation in which we observed subregional hypomethylation of centromeric DNA relates to the sites of transcriptional activity. We identified seven transcriptionally active sites within the 6.76-Mbp region studied. As discussed above, two of these sites correspond to the unspliced ESTs AA811493 and AI024013 that are found at the outer boundary of the centromeric chromatin (island b and islet g, respectively). The remaining five sites are associated with the expressed genes FLJ10188, KIAA1600, TRUB1, ATRNL1, and GFRA1 (genes 1–5 in Figure 2D) that are located within the S/MAR–enriched region, including a gene (gene 4) that spans across the CENP-A–binding domain. We showed that all seven expressed loci are associated with hypomethylated sites.

Given the prevalence of hypermethylation in the M10 over the N10 background, it could be intuitively expected that some of the genes embedded in the neocentric chromatin would have undergone silencing. This, however, is not the case, as we have previously demonstrated that all 51 known genes within or near the neocentric chromatin showed the same expression profile in both the M10 and N10 cell lines [32], regardless of whether the genes are expressed or silenced. However, in that study, we did not investigate the relative expression properties of the many annotated ESTs not characterized as genes. Whether some of these ESTs are differentially hypermethylated and therefore silenced following neocentromere formation remains unclear. In this regard, some of the expressed genes within this region [32] do not have a corresponding CpG island. Whether the transcriptional regulation of these genes occurs through the methylation of CpG islets or CpG orphans remains to be determined.

Previous studies have shown that ICF-syndrome patients with a mutation of the DNMT3b gene show centromeric and pericentric chromatin decondensation [26,44], and the possible re-enactment of this phenotype in cells treated with the methylation inhibitor, 5-aza-dC [45]. We investigated the functional significance of DNA hypermethylation at the neocentromere using 5-aza-dC and observed increased anaphase lagging or bridging of chromosomes, indicative of centromere instability caused by 5-aza-dC–induced inhibition of DNA methylation. Specifically, we observed anaphase defects involving the neocentromere-containing M10 chromosome, suggesting neocentromere stability is compromised by the perturbation of DNA methylation.

Immunofluorescence analysis on the 10q25 neocentromere showed a significant decrease in the level of 5MeC and in the levels of the methyl DNA-binding and heterochromatin markers MBD1 and tri-MeK9H3 after 5-aza-dC treatment, suggesting a direct correlation between DNA methylation and the binding of these proteins at the neocentromere. The levels of the constitutive centromeric protein CENP-A, an essential protein for active centromeres [46,47] and the euchromatin marker 5-AcH4 were unaffected by 5-aza-dC treatment. Importantly, we observed a significant reduction of neocentromere-associated methylation in the majority of the CpG islets and orphans selected for further analysis in the presence of 5-aza-dC. Taken together, the results obtained with 5-aza-dC treatment indicate that DNA hypermethylation is important for the functional integrity of the neocentromere. However, as 5-aza-dC is likely to affect the expression profile of many proteins, including those involved in the assembly and modification of the centromere, the contribution of such a generalized genomic effect to centromere instability following drug treatment remains unclear.

Klose et al. [36] recently described the DNA-binding motif for MeCP2 as one of CG[A/T]_4_, CGn[A/T]_4_, CGnn[A/T]_4_, CGnnn[A/T]_4_, or CGnnnn[A/T]_4_ (n denotes any base). We performed in silico analysis of the distribution of CG[n]_0–4_[A/T]_4_ motifs in relation to the 2,041 CpG dinucleotides studied here (Table 2). We found that a large proportion (49.3%) of the CpG-orphan sites analyzed in this study contained a potential MeCP2-binding site when compared to the CpG islets (10.7%) and CpG islands (4.7%). The finding of a relatively greater prevalence of CG[n]_0–4_[A/T]_4_ motifs amongst the CpG orphans compared to the islands is not unexpected as these sequences are, by definition, more AT-rich. Other studies have indicated that chromatin-modification proteins including MeCP2 [36,48], CXXC CGBP protein [49], and MBD1 [50] can bind to a single methylated CpG dinucleotide, supporting the possibility that orphaned CpG dinucleotides could play a vital role in chromatin remodeling and/or maintenance. In our system, we observed a number of differentially methylated sites upon neocentromere formation that are also potential MeCP2-binding sites that could reflect a differential binding of MeCP2 in both states (Figure 3). However, it remains to be empirically determined which and how many of the CG[n]_0–4_[A/T]_4_ motifs found in the CpG islands, islets, and orphans at the neocentromere are directly involved in MeCP2 binding.

We performed further in silico analysis on the relative distribution of individual CpG dinucleotides within the 6.76-Mbp 10q25 region under study. The results indicate that only 3.5% (2,496/70,502) are associated with CpG islands [15,51], with the remaining 14% (10,006/70,502) and 82.5% (58,000/70,502) being present as islets and orphans, respectively. Such a distribution pattern closely reflects that of a genome-wide calculation of CpG dinucleotide composition: CpG islands (8.32%), CpG islets (5.88%), and CpG orphans (85.79%) (Table 3). This analysis reveals that CpG islets and orphaned CpG dinucleotides together constitute by far the bulk of the human genomic CpG dinucleotide pool. Our study showed that changes to the methylation status of islets and orphans are associated with the gain or loss of heterochromatic or transcriptional state. These findings suggest that differential methylation of the genomically abundant and widely distributed repertoire of CpG islets and orphaned dinucleotides, in conjunction with the more widely studied CpG islands, may provide a more encompassing mechanism of genomic control of chromatin and transcriptional status than a mechanism based solely on the CpG islands.

**Table 3:**
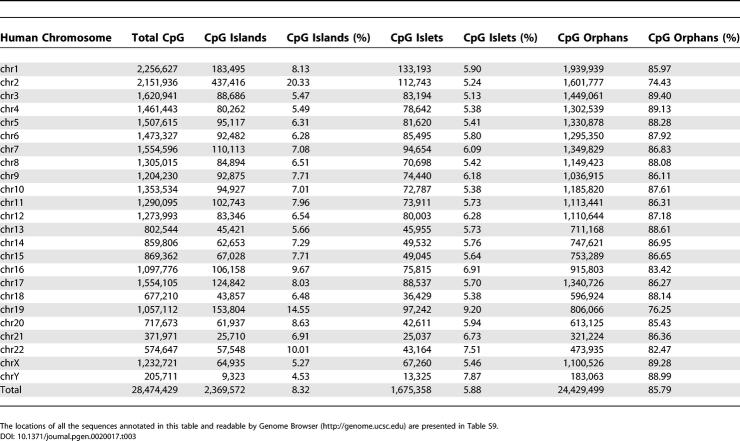
Genome-Wide Distribution of CG Dinucleotides within CpG Islands as Defined by [15], and within CpG Islets and CpG Orphans as Defined in This Study

The centromere has long been thought to be heterochromatic and transcriptionally silent. However, more recent studies in *Arabidopsis,* rice, maize, mice, and humans have demonstrated that transcription can occur within the centromere, suggesting that centromeres are not entirely heterochromatic [32,52–57]. Studies in humans, *Drosophila, Schizosaccharomyces pombe,* and rice have further shown that histone modifications that are associated with both euchromatin and heterochromatin have been found to be associated with the centromere [58–60]. DNA methylation provides an important marker for heterochromatin and is a mechanism for transcriptional regulation, but the detailed pattern of DNA methylation at the centromere has remained poorly understood, mainly reflecting the difficulty associated with the study of conventional, repetitive DNA-based centromeres. We overcame this difficulty using a neocentromere, the sequence of which is fully defined and does not contain tandemly repetitive DNA sequences. We showed that the DNA of a functional centromere is maintained in an overall hypermethylated state, consistent with the general heterochromatic and transcriptionally “silent” characteristics of this structure. Importantly, we demonstrated that pockets of hypomethylation within the centromere provide the necessary chromatin environment to allow transcription to take place without compromising the overall “heterochromatic” state and function of the centromere. In addition to transcription sanctioning, the observed clustering of hypomethylated pockets at the boundaries of a number of centromeric chromatin domains suggests that DNA hypomethylation may serve other important, as yet undefined, roles at these boundary regions.

## Materials and Methods

### Cell culture and genomic DNA isolation.

The two monochromosomal somatic cell hybrid lines utilized in the study, CHO–M10 and CHO–N10, carried M10 or the N10 from which M10 was derived, respectively [32]. Two additional cell lines, containing M10 and derived from mouse F9 and mouse ES cells, were also used in this study [32]. Genomic DNA from these cell lines was isolated using standard phenol/chloroform extraction and ethanol-precipitation procedures. DNA methylation was inhibited by using the methylation inhibitor 5-aza-dC. 5-aza-dC was dissolved in PBS and added to the cell lines at a final concentration of 20 μM. The cell lines were cultured in the presence of the drug over a period of three consecutive days with daily 5-aza-dC–containing media change.

### Sodium bisulfite sequencing.

The DNA sequence of the 10q25 locus was obtained from the UCSC Genome Browser (http://genome.ucsc.edu). CpG islands and CpG islets were selected using CpGReport (http://www.ebi.ac.uk/emboss/cpgplot) with previously described criteria [15]. CpG orphans were selected as regions of DNA with an average GC content of less than 40%. Genomic DNA isolated from CHO–N10 and CHO–M10 was treated with sodium bisulfite [34] or the MethylEasy Kit (Human Genetic Signatures, http://www.geneticsignatures.com). For each sequence assayed, hemi-nested primer sets (Tables S2–S4 and S8) were designed to selectively amplify the fully converted genomic DNA templates after two rounds of PCR. PCR of each methylation site was performed in triplicate and then pooled for cloning into pGEMT-Easy vector (Promega, Madison, Wisconsin, United States). PCR cycling conditions were as follows: denaturation at 95 °C for 3 min, followed by five cycles at 95 °C for 10 s, annealing at 2 °C lower than calculated primer T_m_ [61] for 10 s, and extension at 72 °C for 2 min, followed by 20 cycles at 5 °C for 10 s, annealing at 2 °C lower than calculated primer T_m_ for 10 s, and extension at 72 °C for 1.5 min. Six to eight clones from each genomic DNA template were sequenced to determine the methylation status. The level of methylation was measured as a percentage of methylated sites to the total number of sites assayed for each sequence. Bisulfite profiles for the DNA sequences assayed can be found in Tables S1–S3.

### RT-PCR analysis of ESTs.

Two independent RNA isolations were prepared from CHO–N10 and CHO–M10 cell lines using TriZol Reagent (Invitrogen, Carlsbad, California, United States). Isolated RNA was treated with Turbo RNase-Free DNase (Ambion, Austin, Texas, United States) to remove contaminating genomic DNA. cDNA was synthesized by PCR from 2 μg of total RNA using TaqMan Gold cDNA Synthesis Kit (Applied Biosystems, Foster City, California, United States) using primers to ESTs AA811493 (1F—TTCCCTTTTATTGGAGCTAGG, 1R—AGGTCGTTAAAGATTTCCCC) and AI024013 (1F—AAAGACAACGAAAGACTTGG, 1R—CTCTTTCCAATGTGACAAGG), with TRUB1 (1F—AGCCCGAGGAGTTCTGGTTGTT, 1R—TTTCCCCAGTTCTCCAATGGC) [32] and 18S RNA standards (Ambion) as positive-expression controls. PCR conditions were 40 cycles of denaturation at 95 °C for 10 s and annealing/extension at 55 °C for 10 s.

### Immunofluorescence.

Immunofluorescence was performed as previously described [62] using antibodies raised against tri-methyl lysine 9 modification of histone H3 [63], MBD1 [50], 5MeC (Fitzgerald Industries International, http://www.fitzgerald-fii.com), CENP-A [64], and penta-acetylated histone H4 (Serotec Laboratories, http://www.serotec.com). Texas Red–conjugated fluorescent secondary antibodies (Jackson Laboratories, Bar Harbor, Maine, United States) were used to visualize the primary antibodies. Human anti-serum from a CREST-syndrome patient [27] and an FITC-conjugated fluorescent secondary antibody were used to co-detect and identify the M10 neocentromere. Images were digitally captured using a Zeiss Axioplan II fluorescence microscope (http://www.zeiss.com) with a CCD camera connected to a computer running IP-Lab v3.6.5a software (Scanalytics, http://www.scanalytics.com). Exposure settings were determined qualitatively to ensure that fluorescent signals were not saturated, and were used to capture all images from three independent experiments. The fluorescence signal was measured on CHO–M10 cells treated with 20 μM 5-aza-dC and was compared with the signal from CHO–M10 cells, grown without 5-aza-dC, using IP-Lab v3.6.5a (Table S6). Fluorescent-signal intensity from each antibody was then expressed as a ratio to the fluorescent-signal intensity of the CREST-3 antiserum (Table S7).

### Anaphase analysis.

The M10 cell line was grown on poly-lysine slides immersed in HAM's media supplemented with 10% FCS. The cells were grown in the presence of 5-aza-dC over a period of 3 d, and were synchronized on the final day with 100nM nocodazole (Sigma-Aldrich, St. Louis, Missouri, United States) for 16 h. The cells were released from the mitotic block with three rinses in HAM's media supplemented with 10% FCS. Cells were then grown in HAM's media supplemented with 10% FCS and 10 μM cytochalasin B (Sigma-Aldrich) for 1 h before being fixed with ice-cold methanol and acetic acid (3:1). The slides were subjected to fluorescence in situ hybridization (FISH) using a biotin-labeled BAC probe RP11-153g5 for the M10 neocentromere. The DNA probe was visualized using FITC-conjugated avidin as outlined [62]. Slides were mounted in Vectorshield (Vector Laboratories, http://www.vectorlabs.com) and images were captured as described above.

### In silico analysis of 10q25 neocentromere region.

CpG islands, CpG islets, and CpG orphans analyzed in the current study were annotated in the appropriate format for the UCSC Genome Browser (http://genome.ucsc.edu). Sequence analysis was performed using EMBOSS (http://emboss.sourceforge.net). DNA-sequence data were retrieved from the May 2004 (hg17) reference sequence from the UCSC Genome Browser, and were uploaded as custom annotation tracks (Table S9).

## Supporting Information

Table S1 Table of Control CpG Islands and CpG Islets Used in This Study (381 KB DOC)

Table S2 Bisulfite DNA-Methylation Profiles of CpG Islands and CpG Islets Analyzed in This Study (7.7 MB PDF)

Table S3 Further Bisulfite DNA-Methylation Profiles of CpG Islands and CpG Islets Analyzed in This Study (6.7 MB PDF)

Table S4 Bisulfite PCR Primers Used to Study Methylation of Orphan CpG Dinucleotides (137 KB DOC)

Table S5 Bisulfite PCR Primers Used in This Study (642 KB DOC)

Table S6 Table of Mean Raw Fluorescence Intensity of Proteins Measured at the 10q25 Neocentromere (60 KB DOC)

Table S7 Table of Fluorescence-Intensity Ratios of Chromatin Proteins at the 10q25 Neocentromere before and after 5-aza-dC Treatment (32 KB DOC)

Table S8 Bisulfite PCR Primers Used for Control Regions in This Study (63 KB DOC)

Table S9 Genome-Wide CpG Islet Annotations in 100, 200, and 400-bp Window Sizes Formatted for upload as a custom annotation track into the May 2004 (hg17) reference sequence from the UCSC Genome Browser (http://genome.ucsc.edu).
(5.0 MB ZIP)

### Accession Numbers

The Swiss-Prot (http://www.ebi.ac.uk/swissprot) accession numbers for the proteins discussed in this paper are CENP-A (P49450), CENP-H (P45973), CXXC CGBP (Q9P0U4), DNMT1 (P26358), DNMT3b (Q9UBC3), MBD1 (Q9UIS9), MBD2 (Q9UBB5), MeCP2 (P51608), and penta-acetyl histone H4 (P62805).

The OMIM (http://www.ncbi.nlm.nih.gov/entrez/query.fcgi?db=OMIM) accession number for ICF syndrome is MIM242860.

The GenBank (http://www.ncbi.nlm.nih.gov/Genbank) accession number for the tri-methylated lysine 9 of histone H3 is AF531308.

The Celera (http://www.celera.com) accession numbers for the genes discussed in this paper are FLJ10188 (hCG1781461), KIAA1600 (hCG40995), TRUB1 (hCG40949), ATRNL1 (KIAA0534/hCG39837), and GFRA1 (hCG40963).

## Abbreviations

5-aza-dC: 5-aza-deoxycytidine
5MeC: 5-methylated cytosine
CHO: Chinese hamster ovary
DNMT: DNA methyltransferase
EST: expressed sequence tag
FISH: fluorescence in situ hybridization
ICF: immunodeficiency, chromosome instability, and facial abnormalities
LINE: long interspersed nuclear element
M10: neocentric marker chromosome mardel(10)
MBD: methylated DNA-binding domain
MIR: mammalian interspersed repeat
N10: normal paternal progenitor human Chromosome 10
NHMRC: National Health and Medical Research Council
Op: orphan-containing region
S/MAR: scaffold/matrix attachment regions
SINE: short interspersed nuclear element

## References

[pgen-0020017-b001] Choo KHA (2001). Domain organization at the centromere and neocentromere. Dev Cell.

[pgen-0020017-b002] Pidoux AL, Allshire RC (2004). Kinetochore and heterochromatin domains of the fission yeast centromere. Chromosome Res.

[pgen-0020017-b003] Amor DJ, Kalitsis P, Sumer H, Choo KHA (2004). Building the centromere: From foundation proteins to 3D organization. Trends Cell Biol.

[pgen-0020017-b004] Cleveland DW, Mao Y, Sullivan KF (2003). Centromeres and kinetochores: From epigenetics to mitotic checkpoint signaling. Cell.

[pgen-0020017-b005] Craig JM, Earle E, Canham P, Wong LH, Anderson M (2003). Analysis of mammalian proteins involved in chromatin modification reveals new metaphase centromeric proteins and distinct chromosomal distribution patterns. Hum Mol Genet.

[pgen-0020017-b006] Saffery R, Irvine DV, Griffiths B, Kalitsis P, Wordeman L (2000). Human centromeres and neocentromeres show identical distribution patterns of >20 functionally important kinetochore-associated proteins. Hum Mol Genet.

[pgen-0020017-b007] Karpen GH, Allshire RC (1997). The case for epigenetic effects on centromere identity and function. Trends Genet.

[pgen-0020017-b008] Malik HS, Henikoff S (2001). Adaptive evolution of Cid, a centromere-specific histone in *Drosophila*. Genetics.

[pgen-0020017-b009] Amor DJ, Choo KHA (2002). Neocentromeres: Role in human disease, evolution, and centromere study. Am J Hum Genet.

[pgen-0020017-b010] Chen T, Li E (2004). Structure and function of eukaryotic DNA methyltransferases. Curr Top Dev Biol.

[pgen-0020017-b011] Lander ES, Linton LM, Birren B, Nusbaum C, Zody MC (2001). Initial sequencing and analysis of the human genome. Nature.

[pgen-0020017-b012] Bird AP (1980). DNA methylation and the frequency of CpG in animal DNA. Nucleic Acids Res.

[pgen-0020017-b013] Jabbari K, Bernardi G (2004). Cytosine methylation and CpG, TpG (CpA) and TpA frequencies. Gene.

[pgen-0020017-b014] Bird AP (1986). CpG-rich islands and the function of DNA methylation. Nature.

[pgen-0020017-b015] Gardiner-Garden M, Frommer M (1987). CpG islands in vertebrate genomes. J Mol Biol.

[pgen-0020017-b016] Bird A (1999). DNA methylation de novo. Science.

[pgen-0020017-b017] Antequera F, Bird A (1993). Number of CpG islands and genes in human and mouse. Proc Natl Acad Sci U S A.

[pgen-0020017-b018] Cross SH, Bird AP (1995). CpG islands and genes. Curr Opin Genet Dev.

[pgen-0020017-b019] Wutz A, Smrzka OW, Schweifer N, Schellander K, Wagner EF (1997). Imprinted expression of the Igf2r gene depends on an intronic CpG island. Nature.

[pgen-0020017-b020] Lee JT (2003). Molecular links between X-inactivation and autosomal imprinting: X-inactivation as a driving force for the evolution of imprinting?. Curr Biol.

[pgen-0020017-b021] Feinberg AP, Tycko B (2004). The history of cancer epigenetics. Nat Rev Cancer.

[pgen-0020017-b022] Takai D, Jones PA (2002). Comprehensive analysis of CpG islands in human chromosomes 21 and 22. Proc Natl Acad Sci U S A.

[pgen-0020017-b023] Yoder JA, Walsh CP, Bestor TH (1997). Cytosine methylation and the ecology of intragenomic parasites. Trends Genet.

[pgen-0020017-b024] Xu GL, Bestor TH, Bourc'his D, Hsieh CL, Tommerup N (1999). Chromosome instability and immunodeficiency syndrome caused by mutations in a DNA methyltransferase gene. Nature.

[pgen-0020017-b025] Miniou P, Jeanpierre M, Bourc'his D, Coutinho Barbosa AC, Blanquet V (1997). Alpha-satellite DNA methylation in normal individuals and in ICF patients: Heterogeneous methylation of constitutive heterochromatin in adult and fetal tissues. Hum Genet.

[pgen-0020017-b026] Hansen RS, Wijmenga C, Luo P, Stanek AM, Canfield TK (1999). The DNMT3B DNA methyltransferase gene is mutated in the ICF immunodeficiency syndrome. Proc Natl Acad Sci U S A.

[pgen-0020017-b027] Voullaire LE, Slater HR, Petrovic V, Choo KHA (1993). A functional marker centromere with no detectable alpha-satellite, satellite III, or CENP-B protein: Activation of a latent centromere?. Am J Hum Genet.

[pgen-0020017-b028] Warburton PE (2004). Chromosomal dynamics of human neocentromere formation. Chromosome Res.

[pgen-0020017-b029] du Sart D, Cancilla MR, Earle E, Mao JI, Saffery R (1997). A functional neo-centromere formed through activation of a latent human centromere and consisting of non-alpha-satellite DNA. Nat Genet.

[pgen-0020017-b030] Lo AW, Craig JM, Saffery R, Kalitsis P, Irvine DV (2001). A 330 kb CENP-A binding domain and altered replication timing at a human neocentromere. EMBO J.

[pgen-0020017-b031] Alonso A, Mahmood R, Li S, Cheung F, Yoda K (2003). Genomic microarray analysis reveals distinct locations for the CENP-A binding domains in three human chromosome 13q32 neocentromeres. Hum Mol Genet.

[pgen-0020017-b032] Saffery R, Sumer H, Hassan S, Wong LH, Craig JM (2003). Transcription within a functional human centromere. Mol Cell.

[pgen-0020017-b033] Craig JM, Wong LH, Lo AW, Earle E, Choo KHA (2003). Centromeric chromatin pliability and memory at a human neocentromere. EMBO J.

[pgen-0020017-b034] Clark SJ, Harrison J, Paul CL, Frommer M (1994). High sensitivity mapping of methylated cytosines. Nucleic Acids Res.

[pgen-0020017-b035] Antequera F, Boyes J, Bird A (1990). High levels of de novo methylation and altered chromatin structure at CpG islands in cell lines. Cell.

[pgen-0020017-b036] Klose RJ, Sarraf SA, Schmiedeberg L, McDermott SM, Stancheva I (2005). DNA binding selectivity of MeCP2 due to a requirement for A/T sequences adjacent to methyl-CpG. Mol Cell.

[pgen-0020017-b037] Luo S, Preuss D (2003). Strand-biased DNA methylation associated with centromeric regions in *Arabidopsis*. Proc Natl Acad Sci U S A.

[pgen-0020017-b038] Bartee L, Malagnac F, Bender J (2001). *Arabidopsis* cmt3 chromomethylase mutations block non-CG methylation and silencing of an endogenous gene. Genes Dev.

[pgen-0020017-b039] Heard E (2004). Recent advances in X-chromosome inactivation. Curr Opin Cell Biol.

[pgen-0020017-b040] Bowen NJ, Palmer MB, Wade PA (2004). Chromosomal regulation by MeCP2: Structural and enzymatic considerations. Cell Mol Life Sci.

[pgen-0020017-b041] Stirzaker C, Song JZ, Davidson B, Clark SJ (2004). Transcriptional gene silencing promotes DNA hypermethylation through a sequential change in chromatin modifications in cancer cells. Cancer Res.

[pgen-0020017-b042] Lorincz MC, Dickerson DR, Schmitt M, Groudine M (2004). Intragenic DNA methylation alters chromatin structure and elongation efficiency in mammalian cells. Nat Sruct Mol Biol.

[pgen-0020017-b043] Lewis A, Murrell A (2004). Genomic imprinting: CTCF protects the boundaries. Curr Biol.

[pgen-0020017-b044] Gisselsson D, Shao C, Tuck-Muller CM, Sogorovic S, Palsson E (2005). Interphase chromosomal abnormalities and mitotic missegregation of hypomethylated sequences in ICF syndrome cells. Chromosoma.

[pgen-0020017-b045] Rodriguez MJ, Lopez MA, Garcia-Orad A, Vig BK (2001). Sequence of centromere separation: Effect of 5-azacytidine-induced epigenetic alteration. Mutagenesis.

[pgen-0020017-b046] Sullivan KF, Hechenberger M, Masri K (1994). Human CENP-A contains a histone H3 related histone fold domain that is required for targeting to the centromere. J Cell Biol.

[pgen-0020017-b047] Howman EV, Fowler KJ, Newson AJ, Redward S, MacDonald AC (2000). Early disruption of centromeric chromatin organization in centromere protein A (Cenpa) null mice. Proc Natl Acad Sci U S A.

[pgen-0020017-b048] Nan X, Tate P, Li E, Bird A (1996). DNA methylation specifies chromosomal localization of MeCP2. Mol Cell Biol.

[pgen-0020017-b049] Lee JH, Voo KS, Skalnik DG (2001). Identification and characterization of the DNA binding domain of CpG-binding protein. J Biol Chem.

[pgen-0020017-b050] Hendrich B, Bird A (1998). Identification and characterization of a family of mammalian methyl-CpG binding proteins. Mol Cell Biol.

[pgen-0020017-b051] Takai D, Jones PA (2003). The CpG island searcher: A new WWW resource. In Silico Biol.

[pgen-0020017-b052] Copenhaver GP, Nickel K, Kuromori T, Benito MI, Kaul S (1999). Genetic definition and sequence analysis of *Arabidopsis* centromeres. Science.

[pgen-0020017-b053] Fukagawa T, Nogami M, Yoshikawa M, Ikeno M, Okazaki T (2004). Dicer is essential for formation of the heterochromatin structure in vertebrate cells. Nat Cell Biol.

[pgen-0020017-b054] Nagaki K, Cheng Z, Ouyang S, Talbert PB, Kim M (2004). Sequencing of a rice centromere uncovers active genes. Nat Genet.

[pgen-0020017-b055] Lehnertz B, Ueda Y, Derijck AA, Braunschweig U, Perez-Burgos L (2003). Suv39h-mediated histone H3 lysine 9 methylation directs DNA methylation to major satellite repeats at pericentric heterochromatin. Curr Biol.

[pgen-0020017-b056] Topp CN, Zhong CX, Dawe RK (2004). Centromere-encoded RNAs are integral components of the maize kinetochore. Proc Natl Acad Sci U S A.

[pgen-0020017-b057] Lippman Z, Gendrel AV, Black M, Vaughn MW, Dedhia N (2004). Role of transposable elements in heterochromatin and epigenetic control. Nature.

[pgen-0020017-b058] Sullivan BA, Karpen GH (2004). Centromeric chromatin exhibits a histone modification pattern that is distinct from both euchromatin and heterochromatin. Nat Struct Mol Biol.

[pgen-0020017-b059] Volpe TA, Kidner C, Hall IM, Teng G, Grewal SI (2002). Regulation of heterochromatic silencing and histone H3 lysine-9 methylation by RNAi. Science.

[pgen-0020017-b060] Yan H, Jin W, Nagaki K, Tian S, Ouyang S (2005). Transcription and histone modifications in the recombination-free region spanning a rice centromere. Plant Cell.

[pgen-0020017-b061] Marshall OJ (2004). PerlPrimer: Cross-platform, graphical primer design for standard, bisulphite and real-time PCR. Bioinformatics.

[pgen-0020017-b062] Sumer H, Saffery R, Wong N, Craig JM, Choo KHA (2004). Effects of scaffold/matrix alteration on centromeric function and gene expression. J Biol Chem.

[pgen-0020017-b063] Lachner M, O'Carroll D, Rea S, Mechtler K, Jenuwein T (2001). Methylation of histone H3 lysine 9 creates a binding site for HP1 proteins. Nature.

[pgen-0020017-b064] Saffery R, Earle E, Irvine DV, Kalitsis P, Choo KH (1999). Conservation of centromere protein in vertebrates. Chromosome Res.

[pgen-0020017-b065] Smit AF (1999). Interspersed repeats and other mementos of transposable elements in mammalian genomes. Curr Opin Genet Dev.

