# Supplementary material for: Permissive Transcriptional Activity at the Centromere through Pockets of DNA Hypomethylation
Source: PLoS Genet. 2006 Feb 10;2(2):e17. doi: 10.1371/journal.pgen.0020017 (PMC1361766; doi:10.1371/journal.pgen.0020017)
Supplement: Supplementary file 1 [file pgen.0020017.st002.pdf]

Supplementary Table S2a. Bisulfite methylation profiles of CpG islands and CpG islets used in this study.

126 CpG islands and CpG islets were analysed. The “Notes” column includes repeat elements or 5’-gene associated with CpG island or CpG islet. The methylation pattern is expressed as two clusters. Methylation profile expressed as the percentage of methylated CpG dinucleotides to the total assayed. The upper cluster is the profile for N10 while the lower cluster is for M10. Each CpG dinucleotide is represented as a column while each clone sequenced is represented as a row. Methylated and unmethylated dinucleotides are expressed as red and yellow circles respectively. White circles represent sites at which the methylation status was undetermined.

Island/ Islet Number	CpG Report Length	Notes	Difference (M10-N10)	N10	M10	Bisulfite Methylation Pattern
1	70bp		34	32.1	55.1	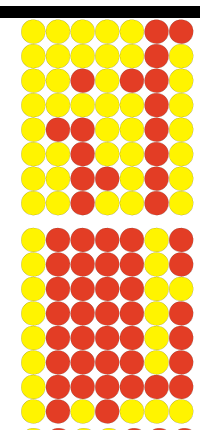
2	82bp		26.5	71.4	100	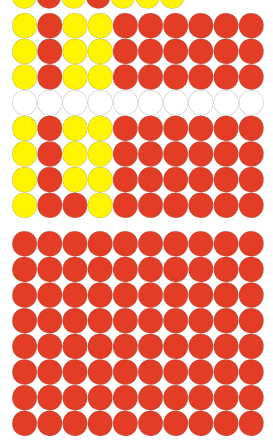

3	70bp	20.4	71.4	91.8
4	92bp	13.7	63.8	77.5
5	230bp	1.7	95	96.7
6	94bp	-8.8	31.3	22.5

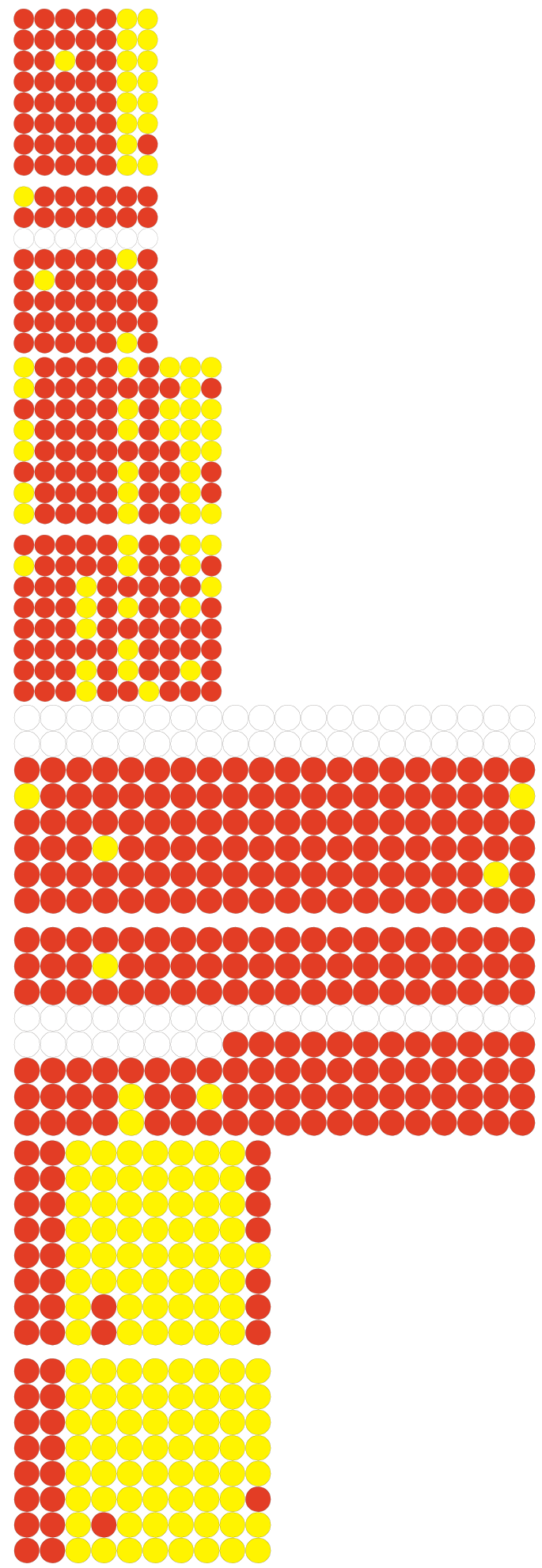

7	54bp	0.9	95.8	96.7
8	60bp	2.5	92.5	95
9	64bp	-8.4	91.7	83.3
10	174bp	-2	96.2	94.2

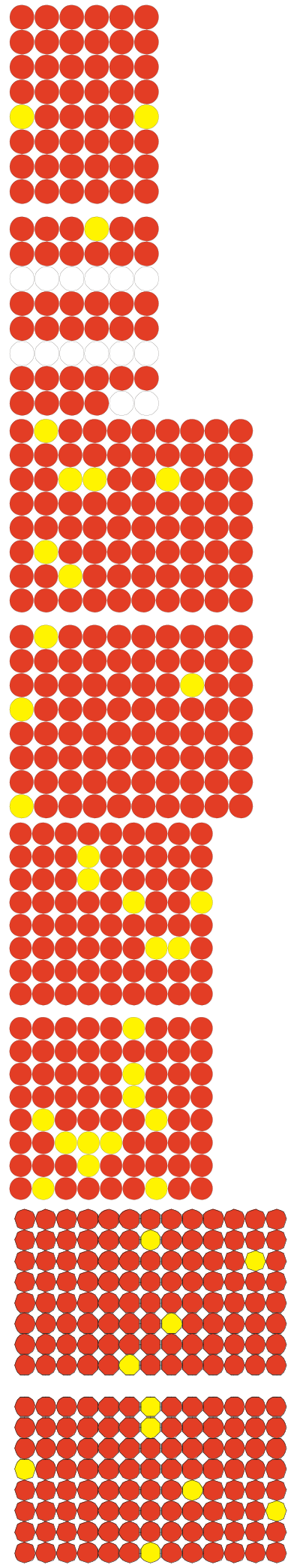

11	115bp	-0.6	97.7	98.1	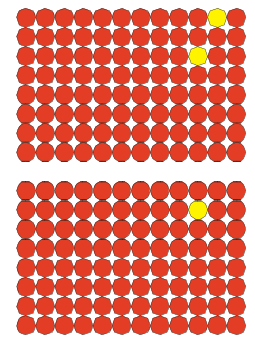
12	81bp	-3.7	99.3	94.9	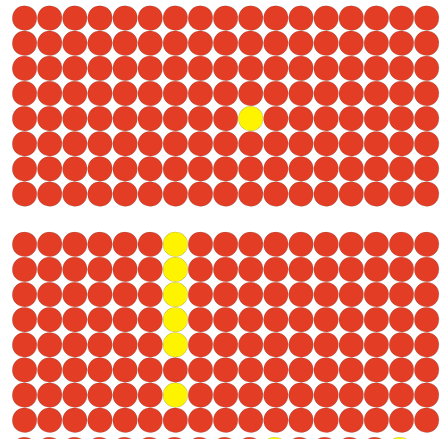
13	116bp	10.8	83.0	93.75	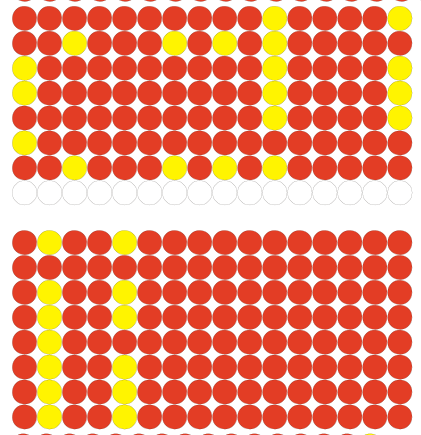
14	63bp	8.4	85.2	93.8	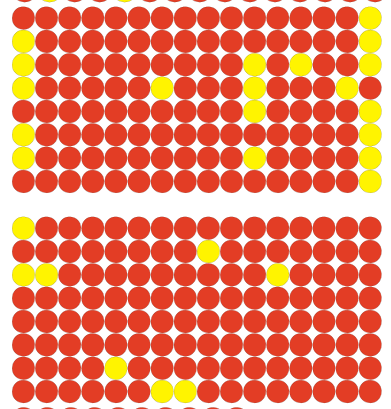
15	64bp	2.5	90	92.5	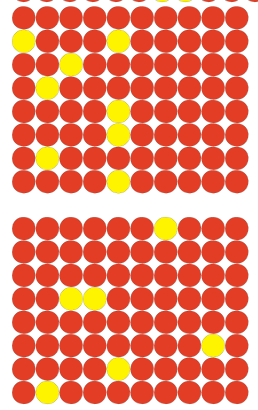

16	92bp		7.8	20.8	28.6	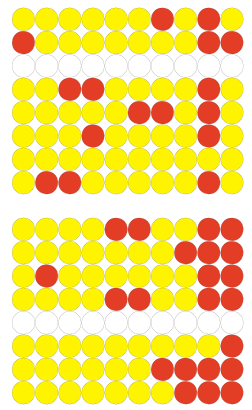
17	97bp	EST - AI024013	-81.8	91	9.2	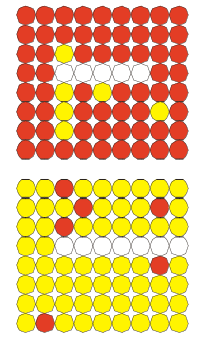
18	117bp		-30.9	73.3	42.3	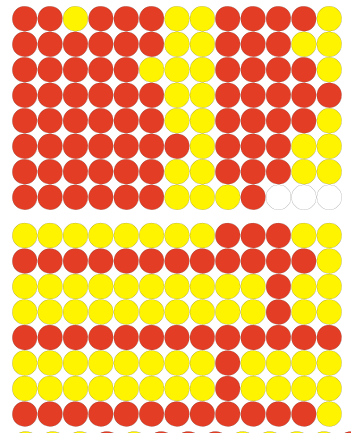
19	97bp		-2.9	35.6	38.5	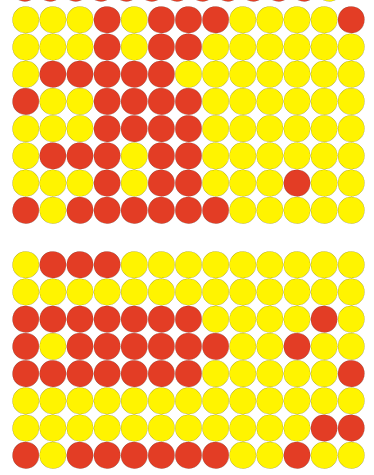
20	83bp		24	69.8	93.8	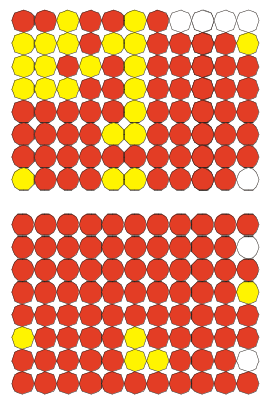

21	56bp	-37.5	75	37.5	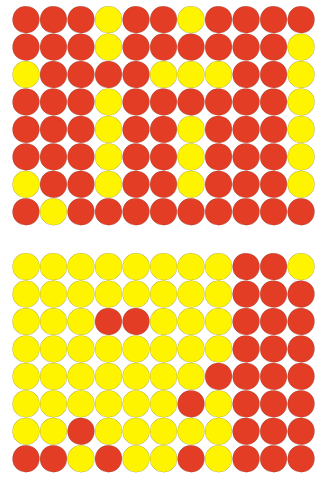
22	160bp	17	72.7	90.3	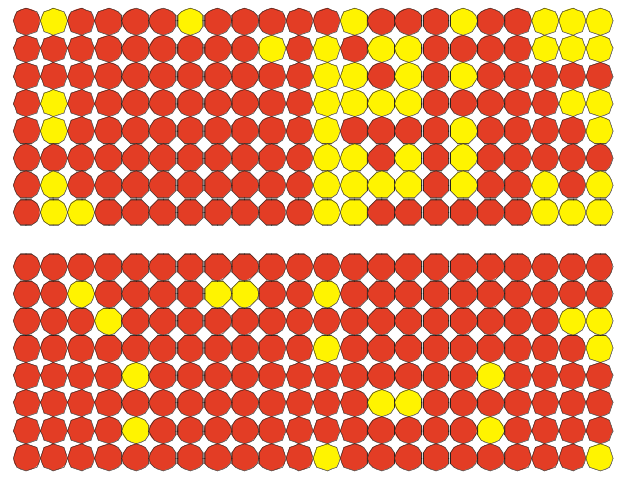
23	162bp	75	22.5	97.5	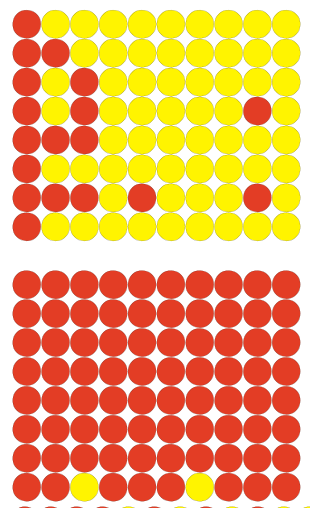
24	119bp	23	70.8	93.8	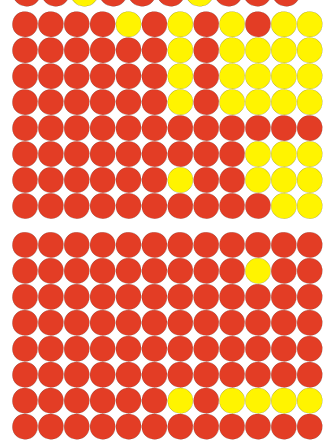

25	51bp		23.2	64.3	87.5	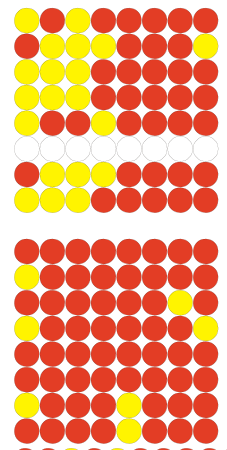
26	58bp	MIRb	15.9	73.9	89.8	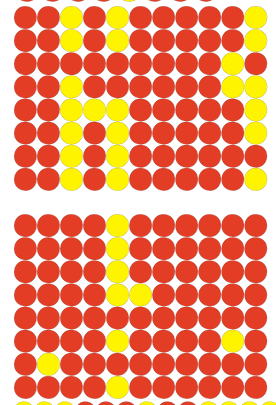
27	80bp		21.3	69.9	91.2	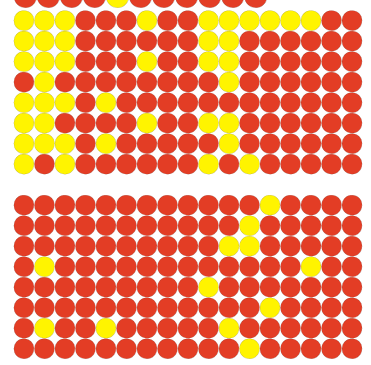
28	126bp		47	48.6	95.8	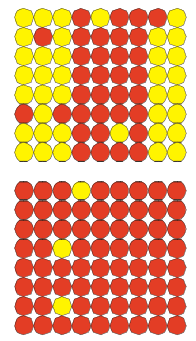
29	78bp		2.1	43	45.1	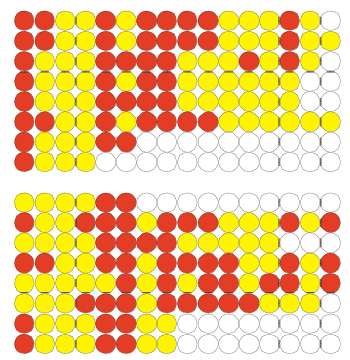

30	52bp	50	35.9	85.9	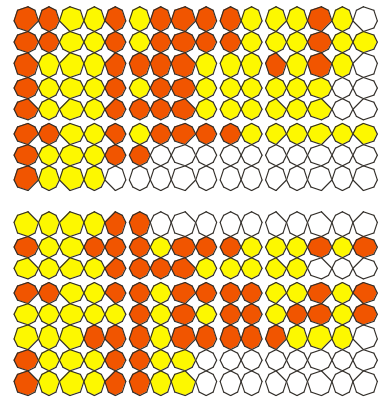
31	75bp	61.4	36.7	98.1	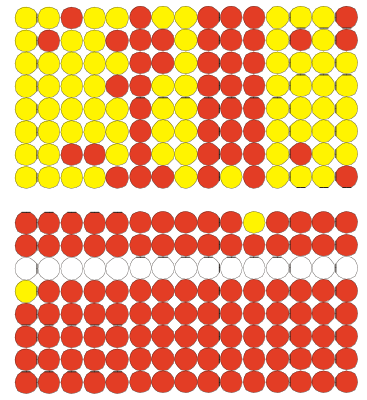
32	62bp	22.2	69.6	91.8	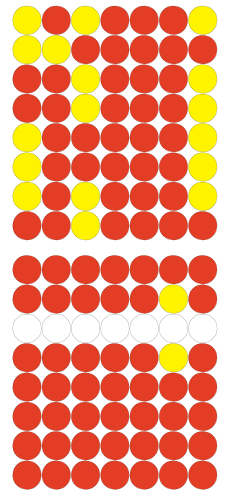
33	127bp	3.8	87.5	91.2	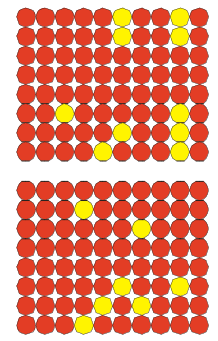
34	434bp	0	0	0	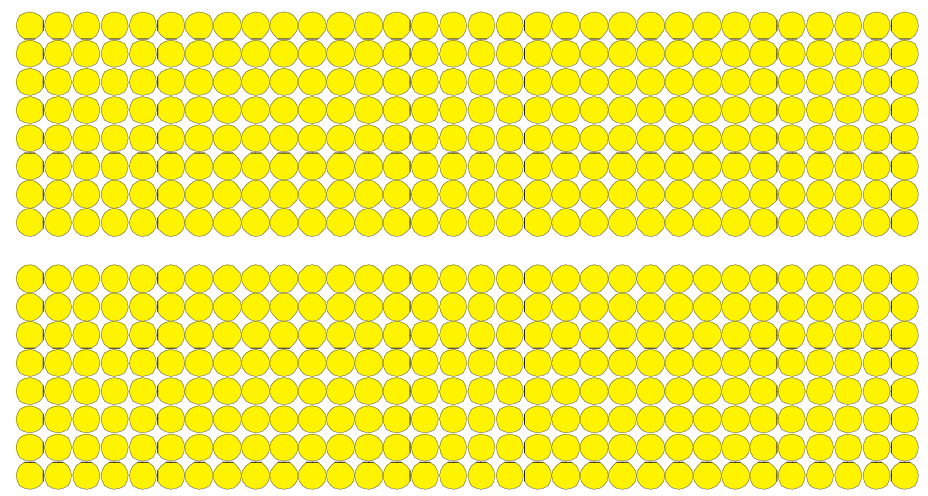

35	53bp	1.8	98.2	100	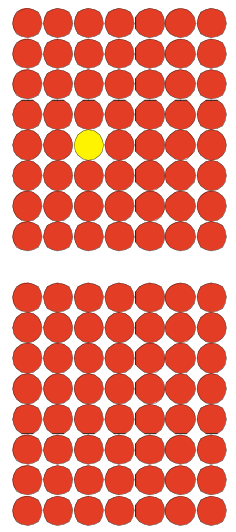
36	63bp	7.8	92.2	100	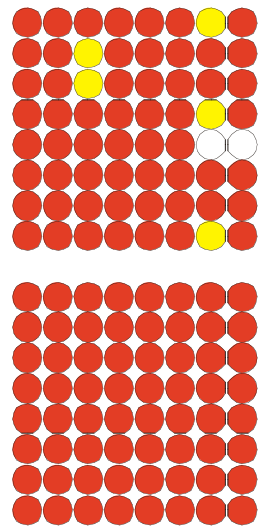
37	96bp	-4.8	90.4	85.6	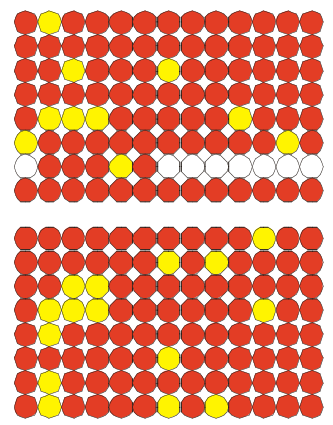
38	88bp	-23.7	59.9	36.2	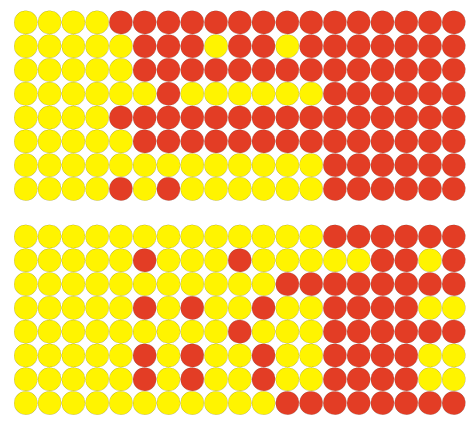

39	2428bp	Gene - ADRB1	7.6	63.4	71	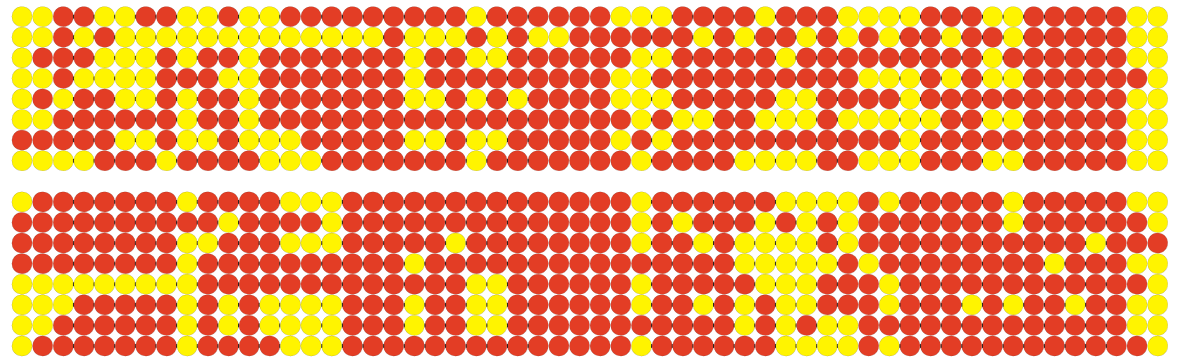
40	121bp		2.8	88.9	91.7	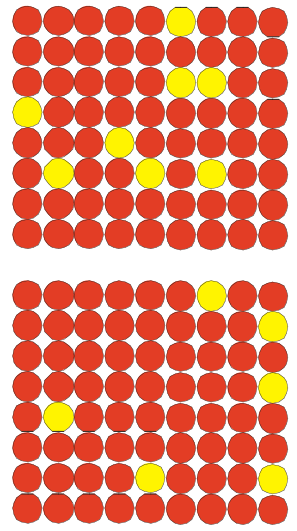
41	482bp		2.7	24.5	27.2	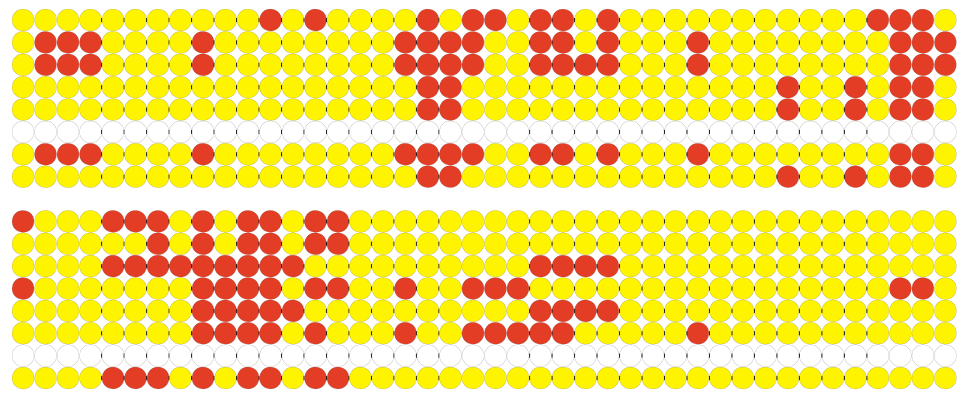
42	61bp		-9.7	90.3	80.6	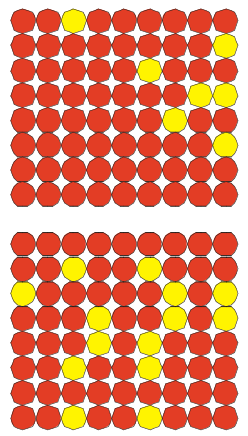

43	72bp		5	95	100	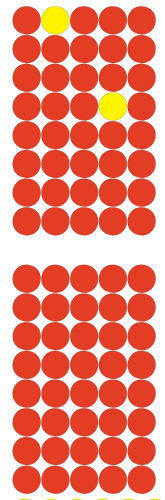
44	134bp		0	0.4	0.4	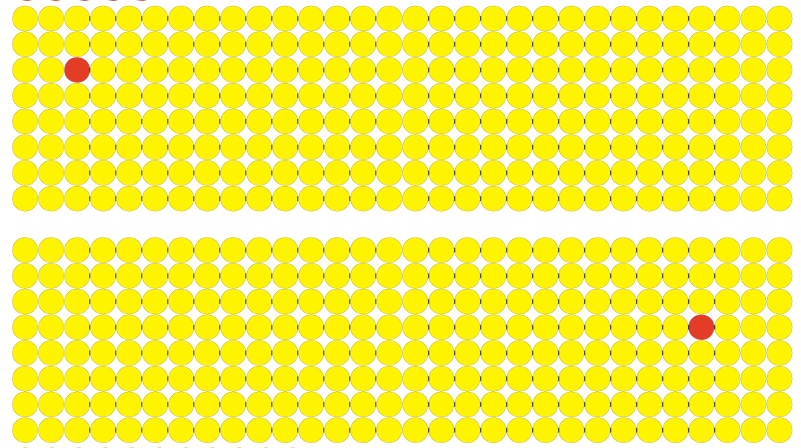
45	241bp	Gene - FLJ10188	-1.6	1.6	0	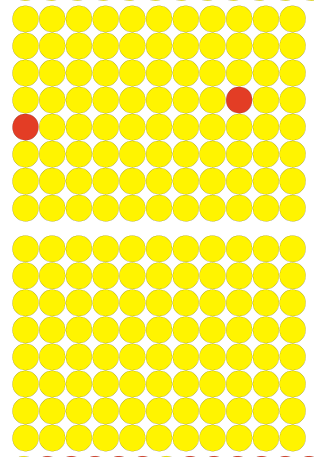
46	119bp		8.6	87.1	95.3	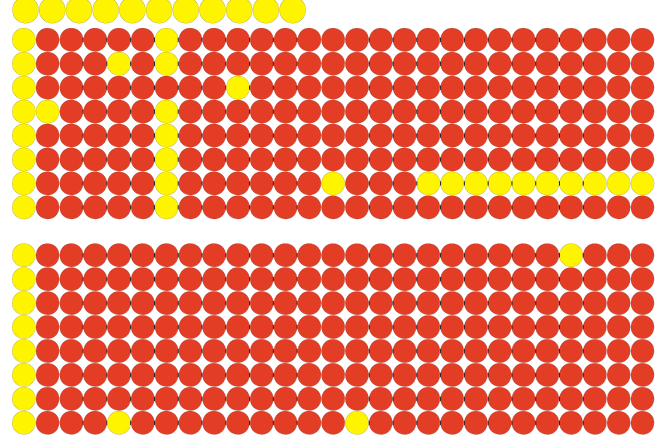
47	666bp	Gene - AMACO	15.1	65.1	80.2	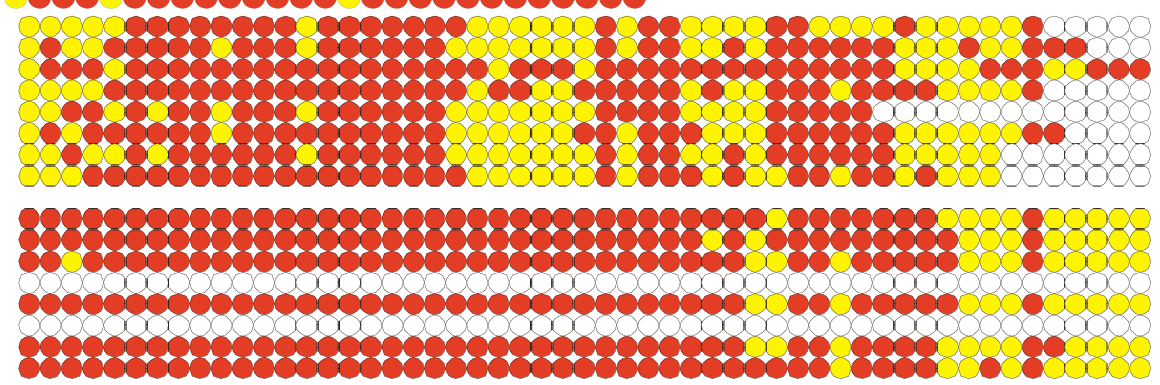

48	60bp	MIRb	39.3	44	83.3	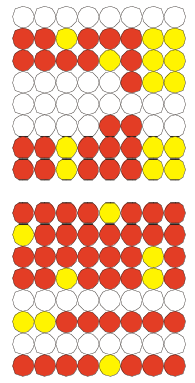
49	200bp		22.2	70.3	92.5	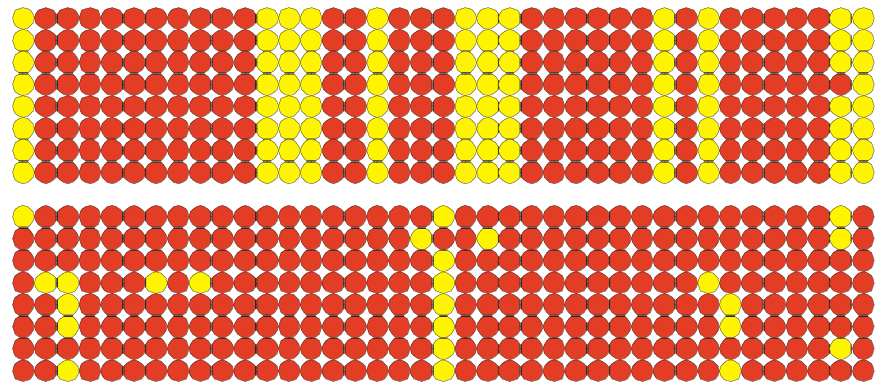
50	104bp		-8.3	10.4	2.1	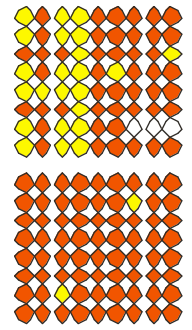
51	89bp		31	66.7	97.2	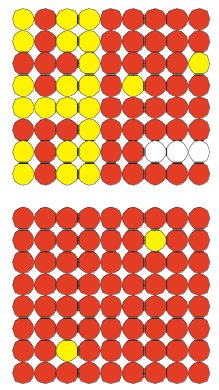
52	94bp		25.2	62.3	87.5	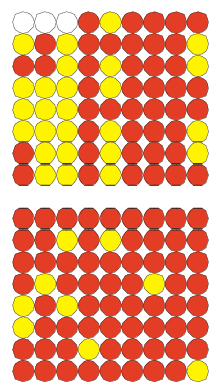

53	113bp		32.9	60.9	93.8	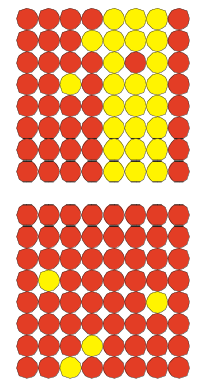
54	/label=73bp		31.6	61.3	92.9	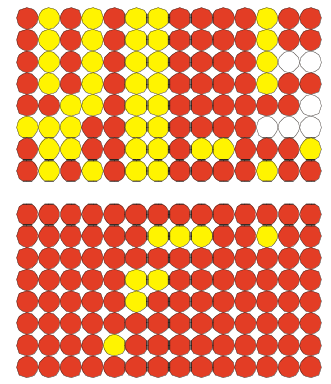
55	76bp		76.6	15.6	92.2	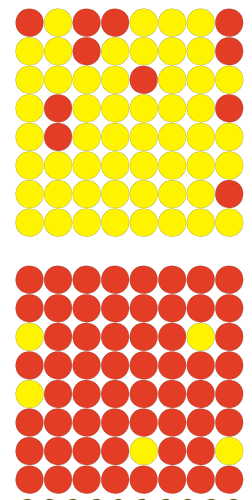
56	734bp		0	0	0	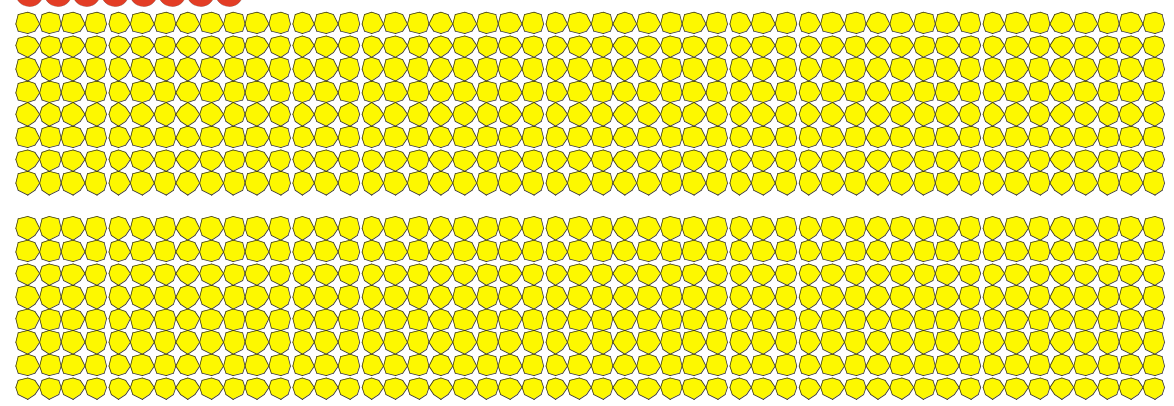
57	790bp	Gene - KIAA1600	0.1	0.8	0.9	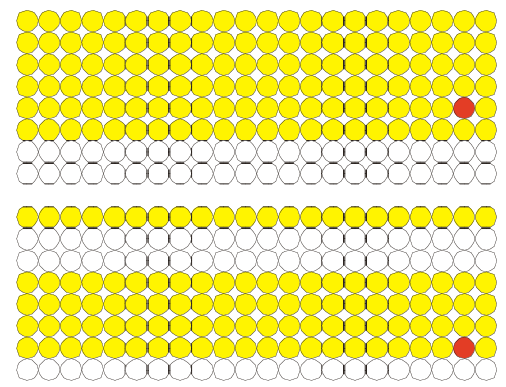

58	59bp	$(CA)_n$	-5	92.5	87.5	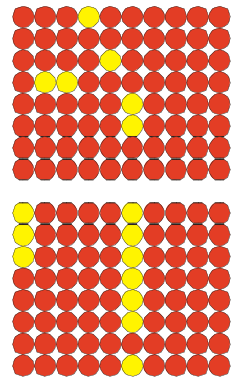
59	413bp	Gene - TRUB1	0	0	0	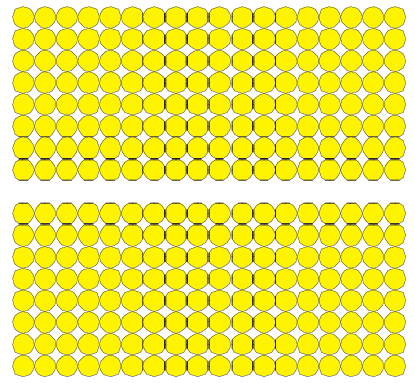
60	90bp	L3	5.3	94.6	100	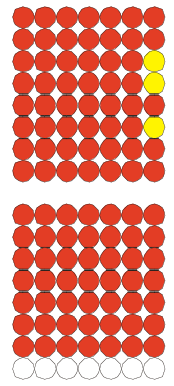
61	81bp	L1PA3	30	63.8	93.8	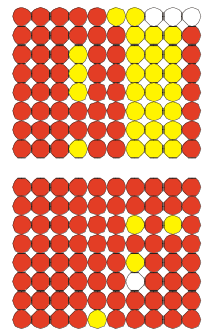
62	1190bp A	Gene - ATRNL1	0.6	2.4	3	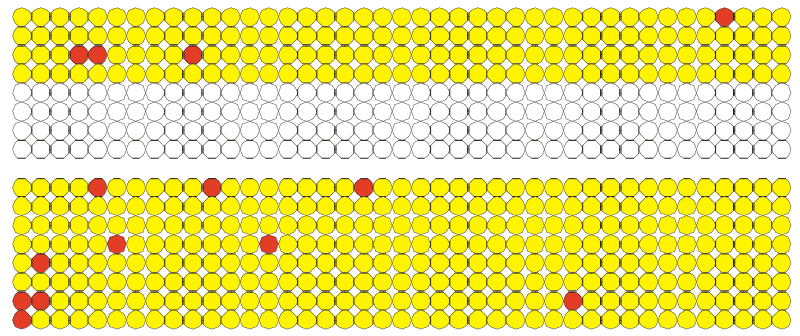
63	1190bp	Gene - ATRNL1	-0.5	0.5	0	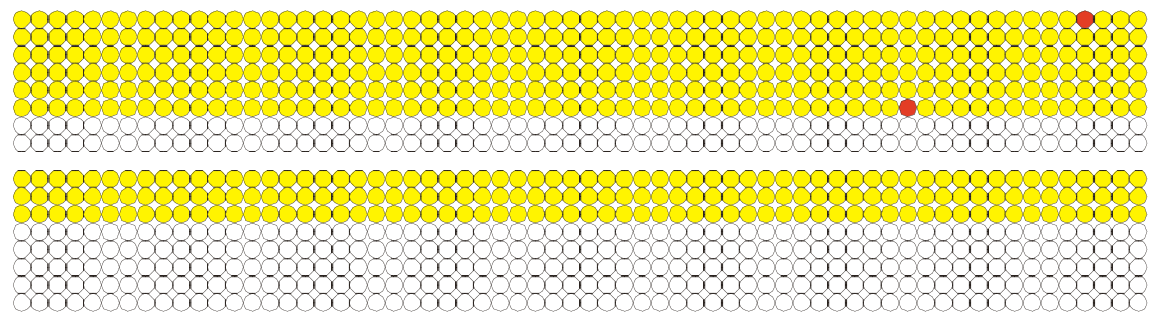

64	114bp		24	76	100	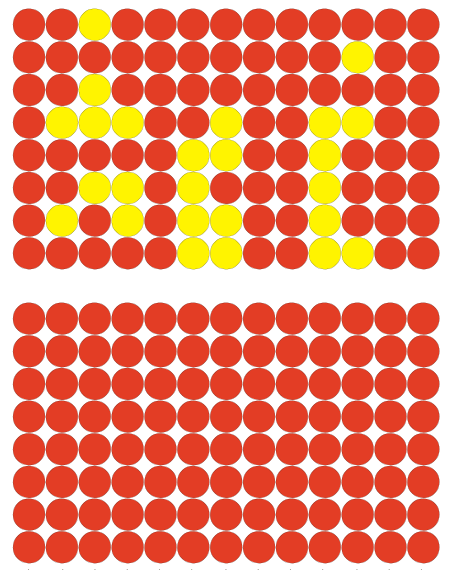
65	78bp	AluSg	9.4	79.7	89.1	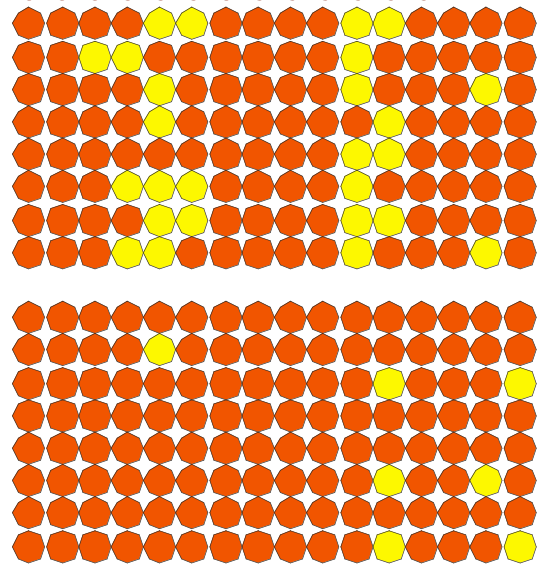
