# Supplementary material for: Permissive Transcriptional Activity at the Centromere through Pockets of DNA Hypomethylation
Source: PLoS Genet. 2006 Feb 10;2(2):e17. doi: 10.1371/journal.pgen.0020017 (PMC1361766; doi:10.1371/journal.pgen.0020017)
Supplement: Supplementary file 2 [file pgen.0020017.st003.pdf]

Supplementary Table S2b. Bisulfite methylation profiles of CpG islands and CpG islets used in this study.

Continuation of the table displaying the 126 CpG islands and CpG islets analysed. The “Notes” column includes repeat elements or 5’-gene associated with CpG island or CpG islet. The methylation pattern is expressed as two clusters. Methylation profile expressed as the percentage of methylated CpG dinucleotides to the total assayed. The upper cluster is the profile for N10 while the lower cluster is for M10. Each CpG dinucleotide is represented as a column while each clone sequenced is represented as a row. Methylated and unmethylated dinucleotides are expressed as red and yellow circles respectively. White circles represent sites at which the methylation status was undetermined.

Island/ Islet Number	CpG Report Length	Notes	Difference (M10-N10)	N10	M10	Bisulfite Methylation Pattern
66	109bp	AluY	34	58.9	92.9	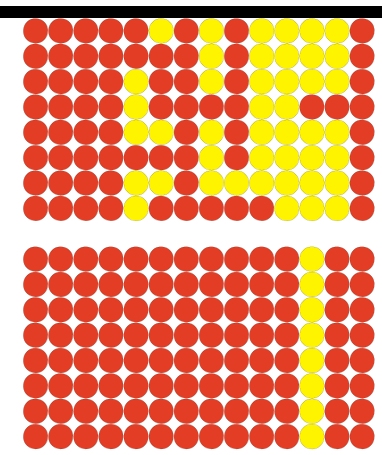
67	69bp	L1R22B/L1ME2	32.5	62.5	90	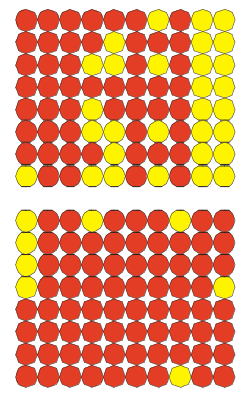

68	200bp	L1PA3	53.3	42.4	95.7	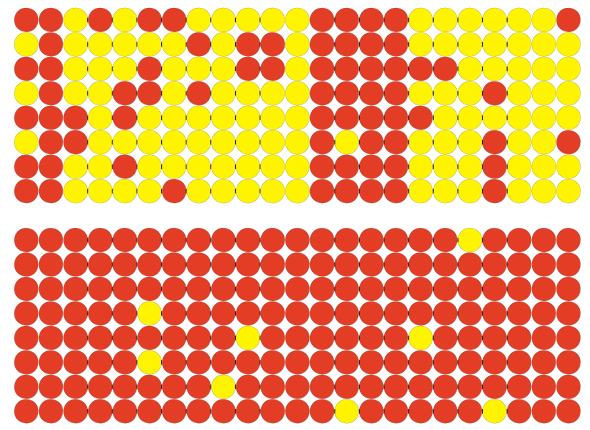
69	61bp		43.8	48.4	92.2	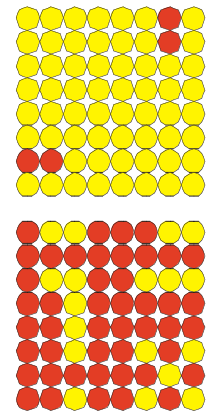
70	73bp		60.7	26.8	87.5	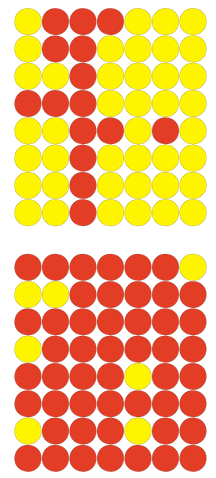
71	58bp	L1ME34	77.5	15	92.5	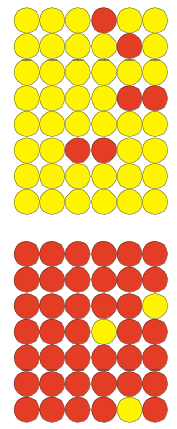

72	90bp		28.5	53.6	82.1	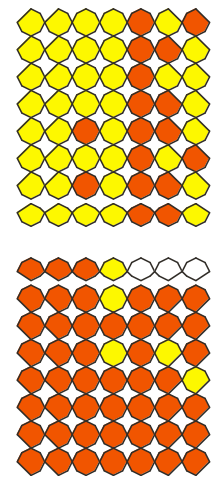
73	90bp		17.2	35.9	53.1	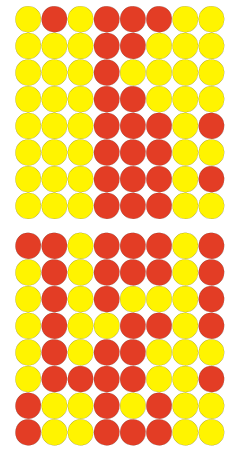
74	52bp	L2-MSTB	58.9	30.4	89.3	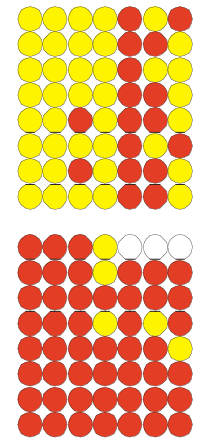
75	53bp		31.2	59.4	90.6	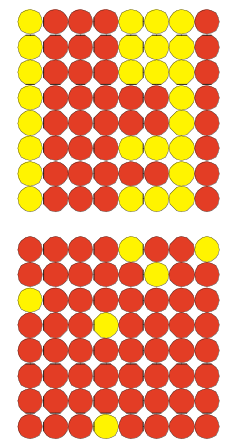

76	68bp		55.2	20.8	76	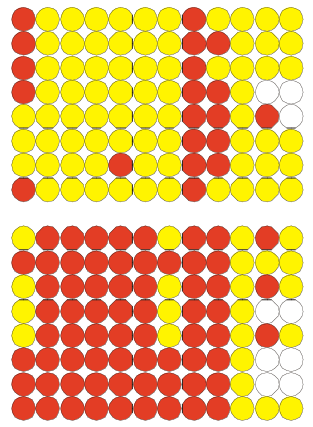
77	396bp	Gene - GFRA1	-0.2	2.6	2.8	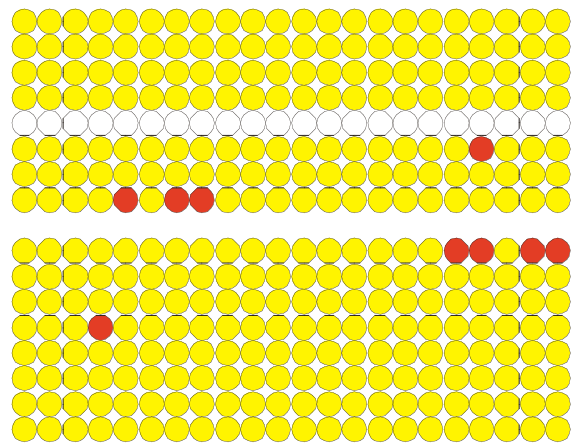
78	118bp		14.7	68.6	83.3	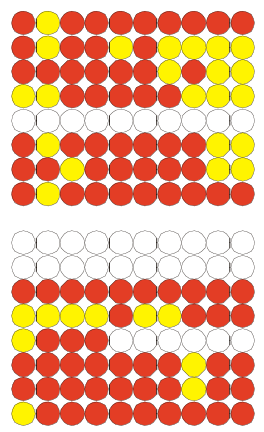
79	87bp		29.2	67.9	92.14	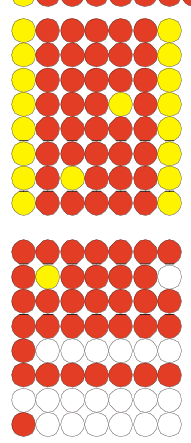
80	313bp	AluY	-6.3	100	93.7	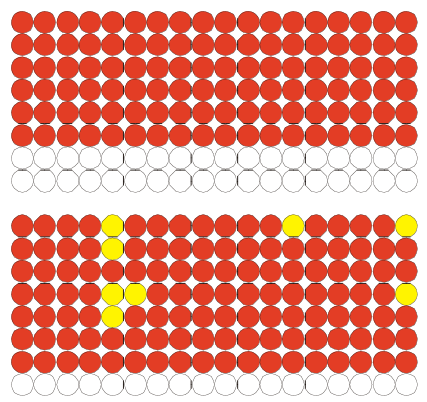

81	116bp	AluSy	49	45.8	94.8	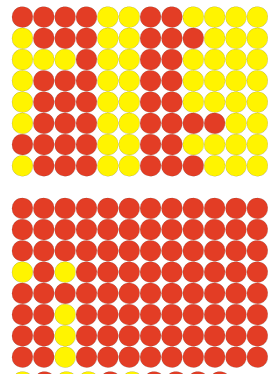
82	70bp	Tigger PLAMB	33.7	58.8	92.5	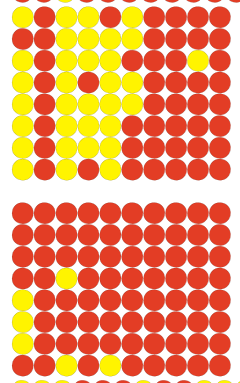
83	79bp	AluY	24.2	46.9	71.1	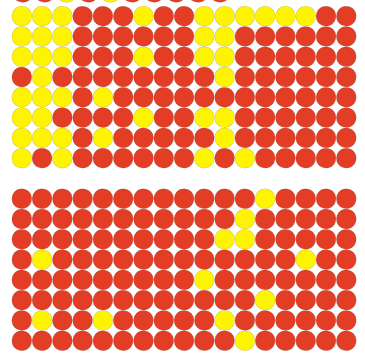
84	79bp	AluY	12.4	78.2	90.6	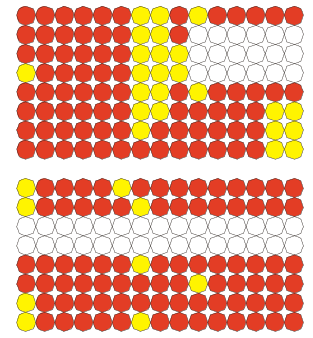
85	149bp	AluY	19.9	65.4	85.3	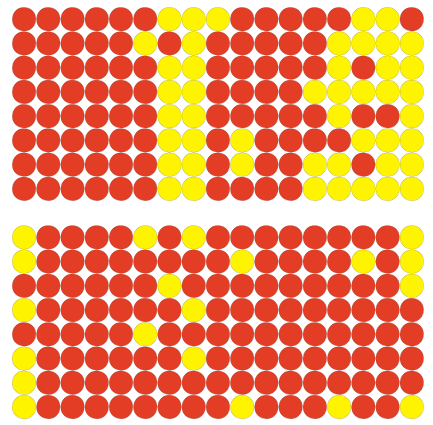
86	190bp	AluY	20.1	75	95.6	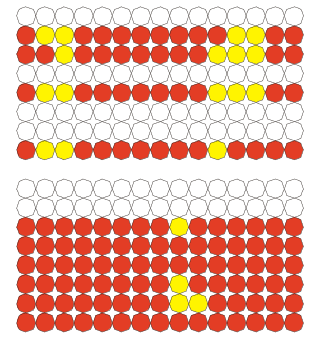

87	142bp	AluY Control	7.3	79.2	86.5	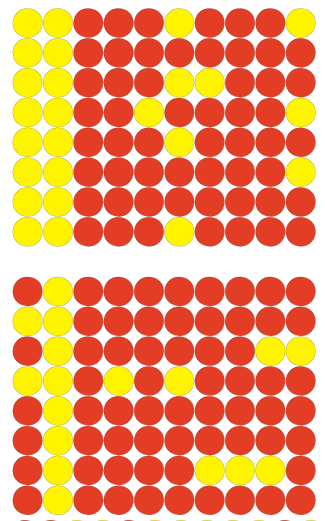
88	142bp	AluY	10	68.8	78.8	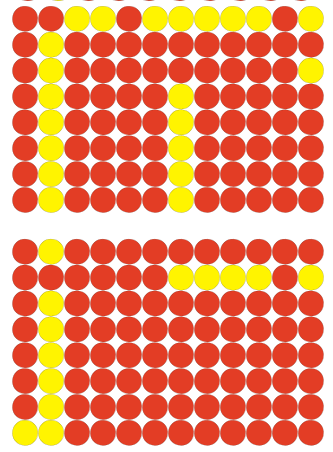
89	51bp		56.3	37.5	93.8	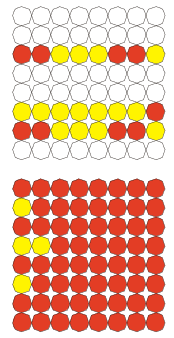
90	50bp	L1ME1	13.5	82.3	95.8	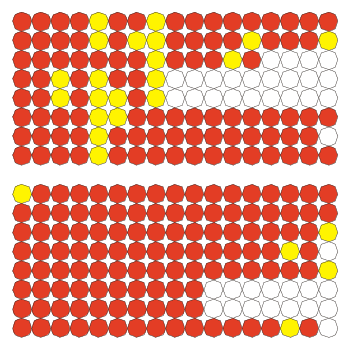
91	104bp	AluSx	21.4	78.6	100	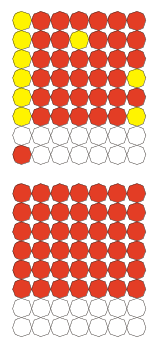

92	67bp		-13	19	6	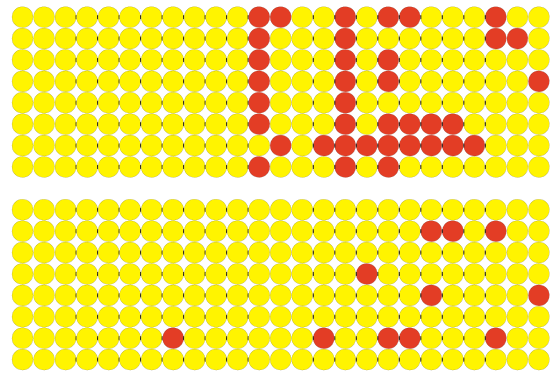
93	141bp		-58.5	65.2	6.7	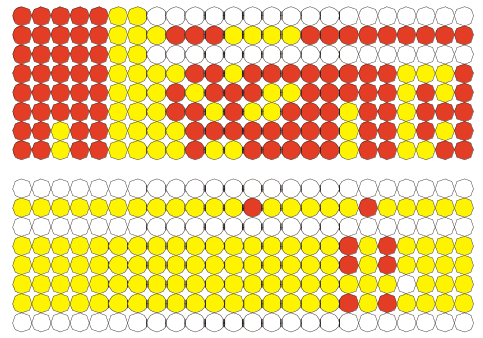
94	544bp		7.1	79.2	86.3	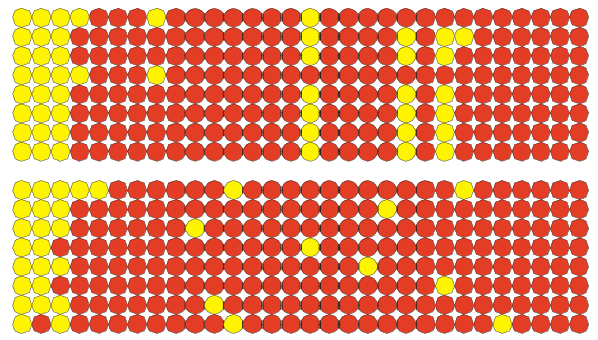
95	102bp	AluY	9.3	94.8	84.4	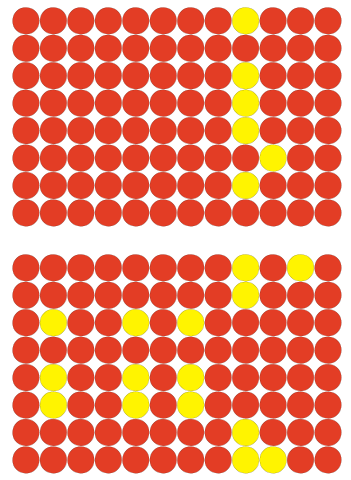
96	51bp		12.3	82.1	94.4	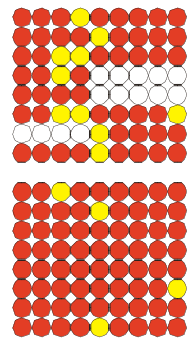
97	102bp	AluY/Charlie	18.3	74	92.3	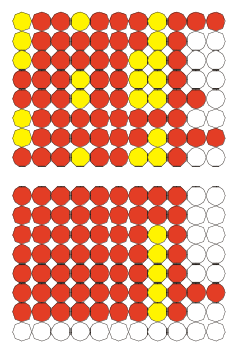

98	71bp	16	70.1	86.1	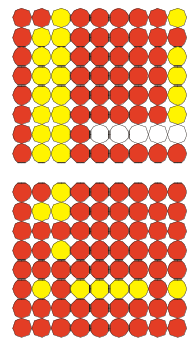
99	89bp	25	72.2	97.2	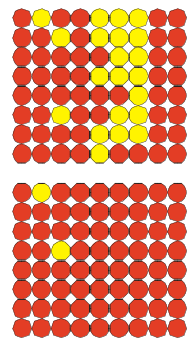
100	53bp	-7.1	100	92.9	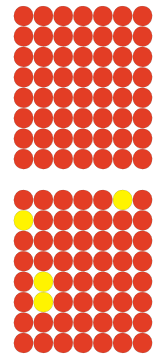
101	50bp	55	42.5	97.5	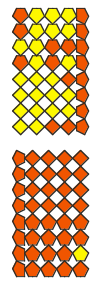
102	63bp	54.2	44.4	98.6	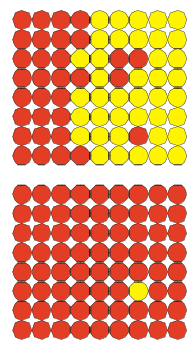
103	61bp	38.9	54.2	93.1	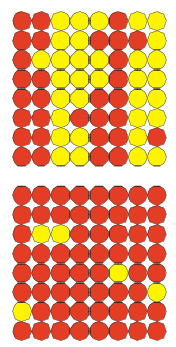

104	111bp		52.3	35.2	87.5	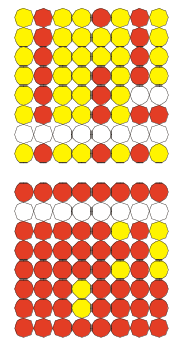
105	98bp		17	73.5	90.5	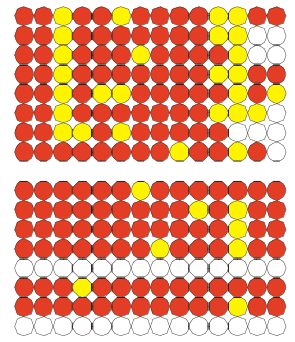
106	1143bp	Gene - HSP12A	1	0	1	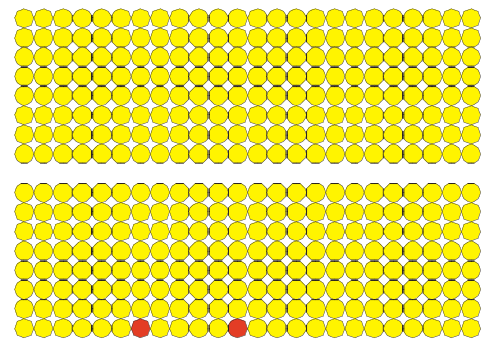
107	78bp	L1MC4a	8.3	45	53.3	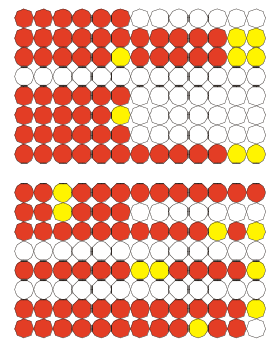
108	78bp		0	87.1	87.1	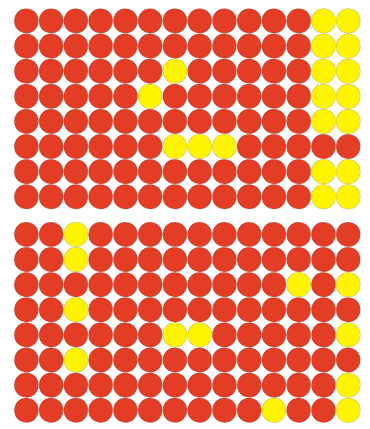
109	449bp	EST - AA811493	-59.1	59.1	0	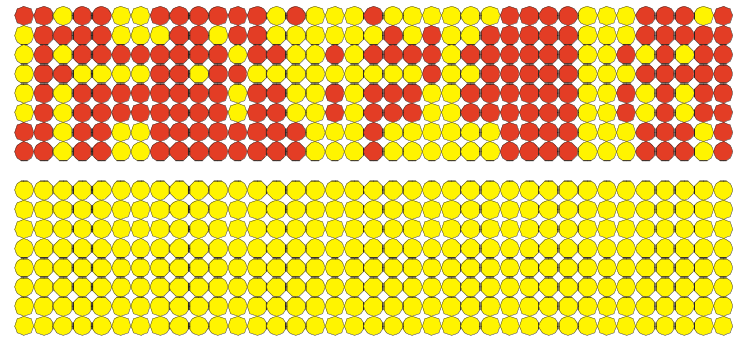

110	99bp	37.5	44.4	81.9	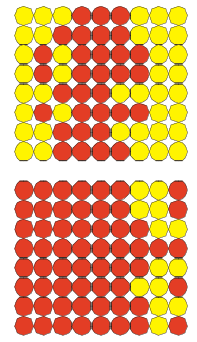
111	88bp	31.5	51.8	83.3	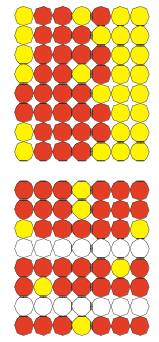
112	58bp	-1.8	92.9	91.1	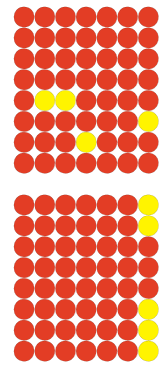
113	943bp	-64.9	65.2	0.3	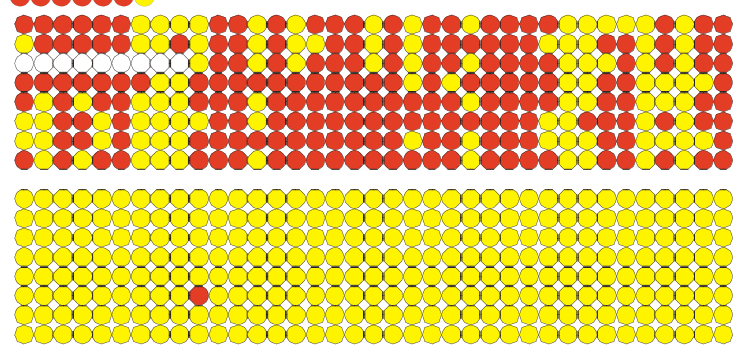
114	58bp	5.4	85.7	91.1	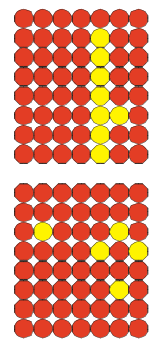
115	307bp	0	0	0	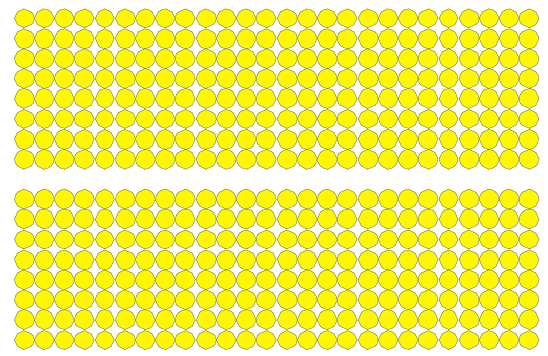

116	154bp	19	61.8	80.1	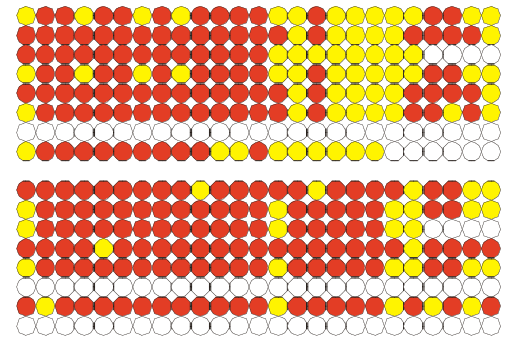
117	165bp	-24.6	25	0.4	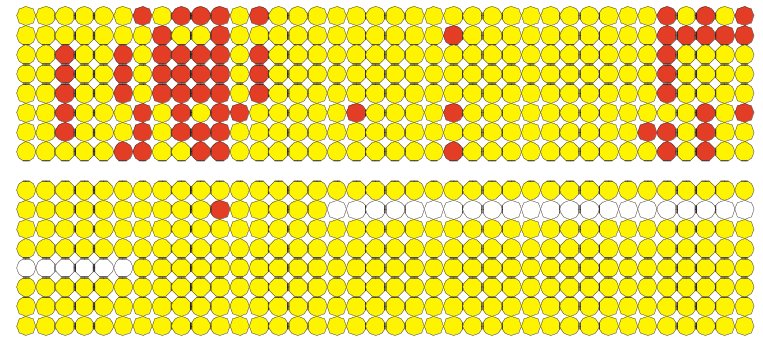
118	396bp	-42.2	51.3	9.1	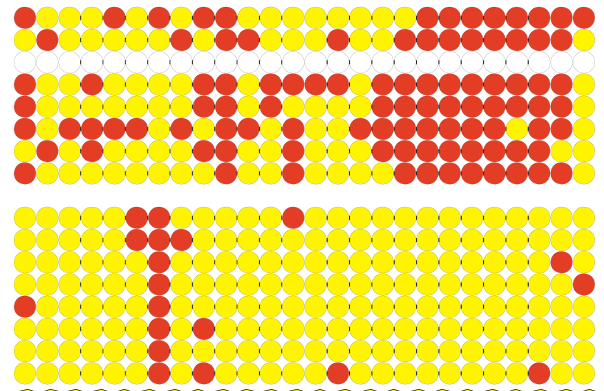
119	937bp	0	0	0	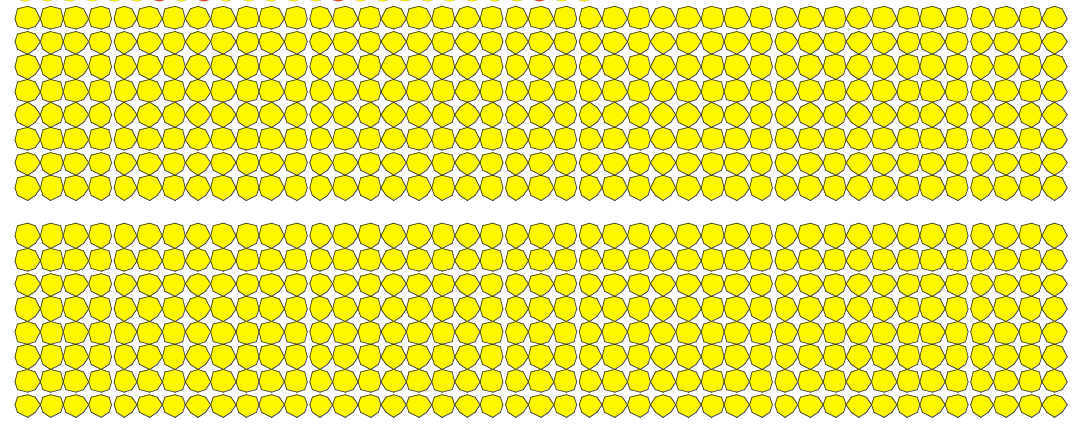
120	50bp	3.8	93.8	97.6	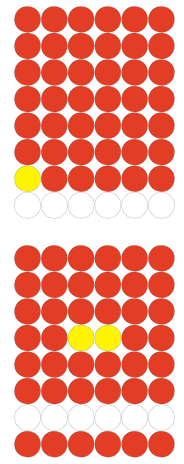

121	69bp	-0.4	0.8	0.4
122	165bp	10.6	11.8	22.4
123	84bp	22.8	63.2	86
124	71bp	34.2	59.8	94

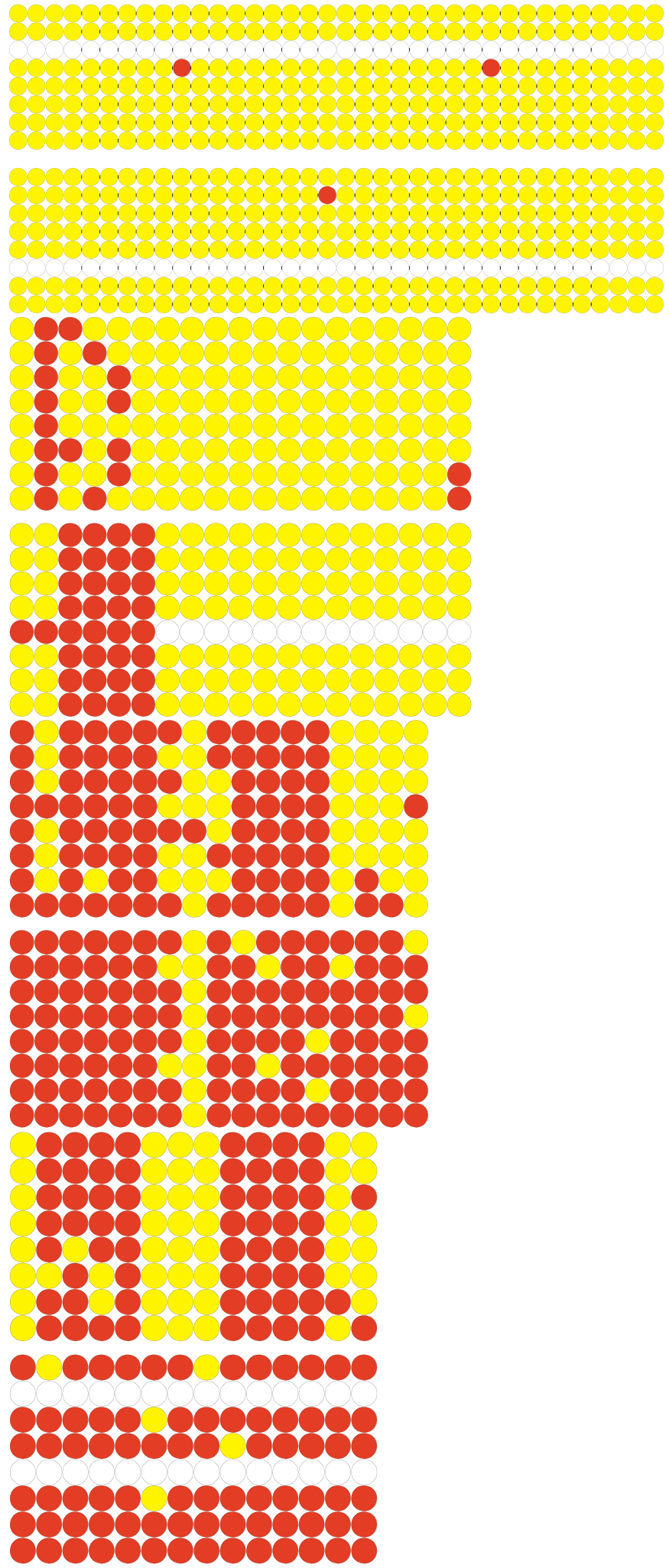

125	91bp	41.6	46.5	88.1	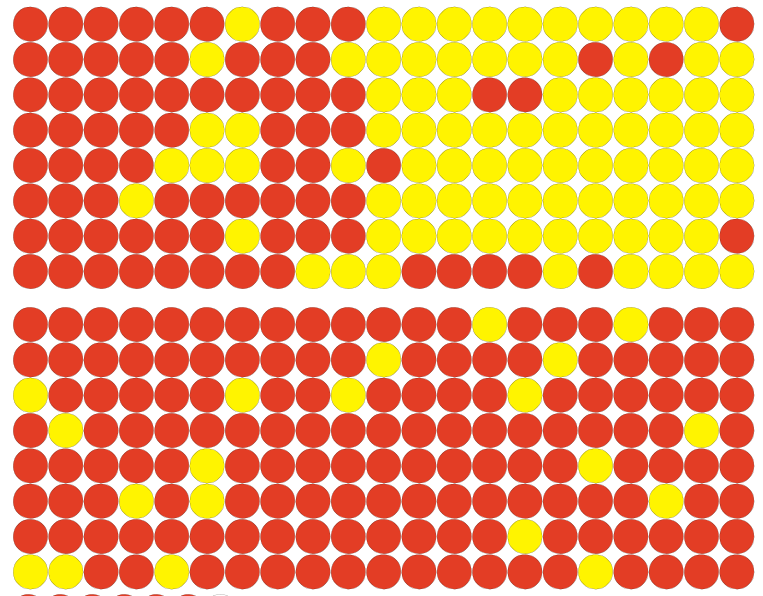
126	54bp	2.1	95.8	97.9	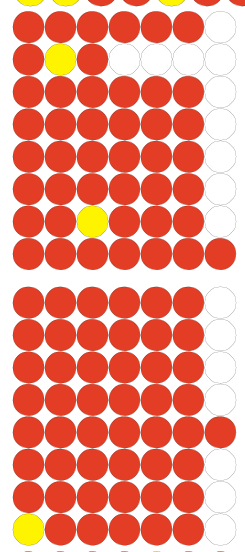
127	74bp	-2.5	65.6	63.1	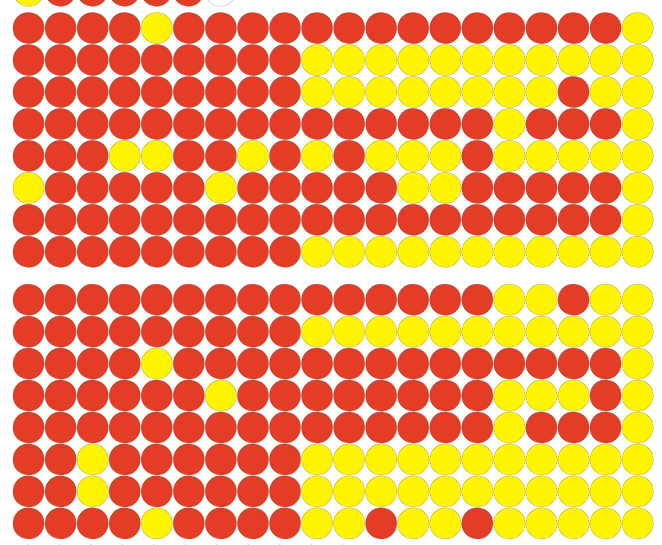
128	61bp	11.4	79.5	90.9	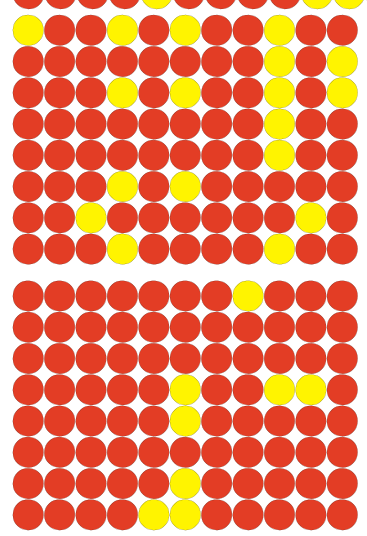
