# Supplementary material for: Permissive Transcriptional Activity at the Centromere through Pockets of DNA Hypomethylation
Source: PLoS Genet. 2006 Feb 10;2(2):e17. doi: 10.1371/journal.pgen.0020017 (PMC1361766; doi:10.1371/journal.pgen.0020017)
Supplement: Supplementary file 3 [file pgen.0020017.st001.doc]

**Supplementary Table 1.** **Table of Control CpG islands and CpG islets used in this study.**

The values are displayed in Figure 1 of the main text. The methylation pattern is display in two clusters. The upper cluster is the methylation pattern for N10 while the lower represents M10. Each column of circles represent one CpG dinucleotide assayed within the CpG islet or island. Each row of circles represents one clone sequenced. Red circles denote the CpG site is methylated while yellow circles denote an unmethylated CpG dinucleotide.

| CpG Island/islet | Size | Gene/ Element | Methylation  Difference (M10-N10) | Methylation Level  N10 | Methylation  Level  M10 | Methylation Pattern |
| --- | --- | --- | --- | --- | --- | --- |
| 10p14 – 1 | 211bp |  | 0 | 0 | 0 |  |
| 10p14 – 2 | 99bp |  | -0.3 | 85.8 | 85.2 |  |
| 10p14 – 3 | 1115bp | CUGBP2 | -0.2 | 0.4 | 0.2 |  |
| 10p14 – 4 | 59bp | MIR | -2.2 | 88.9 | 86.7 |  |
| 10q26 – 1 | 1112bp | FAM24B | 0 | 0 | 0 |  |
| 10q26 – 2 | 77bp | AluSx | 8.8 | 67.8 | 76.6 |  |
| 10q26 – 3 | 123bp |  | 0.5 | 94.8 | 94.3 |  |
| 10q26 – 4 | 62bp |  | -5 | 90 | 85 |  |
| 10q26 – 5 | 83bp |  | -3.2 | 88.2 | 85.0 |  |
