# Supplementary material for: Permissive Transcriptional Activity at the Centromere through Pockets of DNA Hypomethylation
Source: PLoS Genet. 2006 Feb 10;2(2):e17. doi: 10.1371/journal.pgen.0020017 (PMC1361766; doi:10.1371/journal.pgen.0020017)
Supplement: Supplementary file 4 [file pgen.0020017.st004.doc]

**Supplementary Table 3.** **Bisulfite PCR primers used to study methylation of Orphan CpG dinucleotides.**

The GC-content of the region amplified ranged from 0.28 to 0.41 as shown in this table. The primers used in this study are listed in the Bisulfite Sequence column. The unconverted sequence for each primer is also listed here. The Tm was calculated using PerlPrimer (http://perlprimer.sourceforge.net). The co-ordinates for each primer are given according to the hg17 May 2004 assembly of the human genome sequence.

|  |  | **Bisulfite sequence** | **Tm** | **Start** | **End** | **Original Primer Sequence** | **GC-Content** | **CG** |
| --- | --- | --- | --- | --- | --- | --- | --- | --- |
| Op-1 | 1F | ttaaagtttgtttagtaaggtgatttttt | 59.91 | 119639727 | 119639757 | ccaaagtttgttcagtaaggtgacttcccc | 0.42 | 4 |
| Op-1 | 2F | agggaaggtttttagagattttattgttat | 60.58 | 119639649 | 119639679 | agggaaggtttctagagattccactgtcac |  |  |
| Op-1 | 1R | cataaccttataaatatattcaaaactaacctaaa | 59.7 | 119639316 | 119639346 | ccttgtaaatatattcaagactggcctggg |  |  |
| Op-2 | 1F | taaagaatagtttaaattttaggttaagtt | 57.07 | 119401402 | 119401432 | taaagaacagcttaaaccccaggccaagcc | 0.33 | 4 |
| Op-2 | 1R | tattatcataaaataatcctccctaaaaaa | 57.89 | 119400894 | 119400924 | tgttatcctgaggtgatcctccctgaaagg |  |  |
| Op-2 | 2R | acttaacataaaaaccaatatttcataaa | 57.44 | 119400941 | 119400971 | cacttaacataggagccagtatttcatggg |  |  |
| Op-3 | 1F | ttttatttagtagttggttataagaatttt | 57.12 | 119112900 | 119112931 | tttctatttagtagctggctacaagaatctc | 0.32 | 3 |
| Op-3 | 2F | tttgtttttagatataatttttatggagatatt | 58 | 119112846 | 119112876 | gttctcagatacaactttcatggagacatc |  |  |
| Op-3 | 1R | cccataacaaaaatttaccattctaa | 58.54 | 119112414 | 119112439 | cccatggcaagagtttgccattctgg |  |  |
| Op-4 | 1F | aaattgtggataatgtaaaaatttaaattt | 57.33 | 118090958 | 118090988 | aaactgtggacaatgcaaaaacctaaactc | 0.33 | 2 |
| Op-4 | 1R | tattaacaaataatactactcaaatacaaa | 56.97 | 118090447 | 118090478 | atattagcaggtagtactgctcagatacagg |  |  |
| Op-4 | 2R | atttaaattactcaaacattaaaaccttaa | 57.93 | 118090602 | 118090632 | atttgaattgctcaagcattgagaccttgg |  |  |
| Op-5 | 1F | agttatttttgagtttgattttatgtatttat | 58.42 | 117920759 | 117920789 | ttatttctgagcctgatctcatgtacctac | 0.38 | 3 |
| Op-5 | 1R | ctataacctcctatctaaaaatcctaataa | 58.65 | 117920372 | 117920402 | ctgtaacctcctgtctaaaaatcctggtag |  |  |
| Op-5 | 2R | aatcaccctcacaatatttacaaa | 58.79 | 117920475 | 117920498 | agtcaccctcacagtatttgcagg |  |  |
| Op-6 | 1F | gttaaatattagtggagttatagtttgtatttt | 59.55 | 117885819 | 117885852 | gctaaatattagtggagctacagtttgtacccc | 0.35 | 2 |
| Op-6 | 2F | aaaagtaagggttgttttttgtgtt | 60.85 | 117885677 | 117885701 | aaaagcaagggctgctctctgtgcc |  |  |
| Op-6 | 1R | taaatttcaatttaataataatcaatctaaaacaaa | 58.38 | 117885323 | 117885353 | tcaatttagtaatagtcagtctaagacagg |  |  |
| Op-7 | 1F | gttattttattttgtgagtaatttagattgttatatt | 59.75 | 117670534 | 117670562 | attctgtgagcaacttagactgtcatatc | 0.30 | 3 |
| Op-7 | 1R | aaactcatttactaaaaatccccaaaa | 60.35 | 117670074 | 117670101 | gagactcatttgctaaaggtccccaggg |  |  |
| Op-7 | 2R | aataaccttacctaattaaccaaaatctaaa | 59.71 | 117670161 | 117670191 | aatgaccttgcctggttaaccaaaatctgg |  |  |
| Op-8 | 1F | gtaaggtatttgaatgtgtgatatgttaat | 60.7 | 117458795 | 117458821 | gcaaggtatctgaatgtgtgacatgccttc | 0.30 | 4 |
| Op-8 | 1R | ccttaccatatctaatacatcattatctaaaaa | 60.09 | 117458116 | 117458149 | ccttgccatatctgatacatcattgtctgaagg |  |  |
| Op-8 | 2R | aactcttactaattctatatatttactaatatctataata | 58.13 | 117458279 | 117458309 | ggttctgtgtatttgctaatgtctgtggtg |  |  |
| Op-9 | 1F | ttgaagaattaaagttgtaagtatttataaattaatt | 59.31 | 117196494 | 117196524 | atcaaagctgtaagtatctataaatcaacc | 0.32 | 3 |
| Op-9 | 2F | ttaaagattgagtttagaggttagaattat | 59.31 | 117196457 | 117196485 | ttaagactgagcccagaggttagaaccac |  |  |
| Op-9 | 1R | tttaaaacctaaaatataaataattttatattctccta | 58.6 | 117196202 | 117196231 | ctgaagtatgaatggttttgtattctcctg |  |  |
| Op-10 | 1F | tgtttttattataagggtttattggatatt | 58.5 | 116992176 | 116992206 | tgttttcactacaagggtctactggatacc | 0.28 | 3 |
| Op-10 | 1R | aaaatacaaaaactaaatttaaatatccttttaaaa | 59.19 | 116991724 | 116991753 | aaaagctagatttggatgtcctttttgaag |  |  |
| Op-10 | 2R | tttccctttataaacactatatcaatttattata | 58.71 | 116991770 | 116991800 | cctttgtgaacactatatcagtttattgtg |  |  |
| Op-11 | 1F | tattagatttgttttaagaatatttggttt | 57.57 | 116895188 | 116895217 | tactagatctgttccaagaatacttggctc | 0.32 | 1 |
| Op-11 | 1R | ctatttatacttaactaactaataatacaataacaa | 58.01 | 116894552 | 116894582 | atgcttgactggctgataatacagtagcag |  |  |
| Op-11 | 2R | atacatacaccaatctaataaaataaacaa | 58.52 | 116894733 | 116894763 | atacatgcaccagtctaataaaatgggcag |  |  |
| Op-12 | 1F | tagttggtttaatatgtaagttgttattat | 58.07 | 116813472 | 116813502 | cagctggttcaatatgcaagctgccatcac | 0.41 | 4 |
| Op-12 | 2F | taaaaggtatgatttatagtaggtttttat | 57.65 | 116813393 | 116813423 | taaaaggcatgacttatagcaggtcctcac |  |  |
| Op-12 | 1R | ataaaatcattctaccaaaattctatctaa | 58.11 | 116813063 | 116813093 | gtgaagtcattctgccagaattctatctgg |  |  |
| Op-13 | 1F | atgttaatttgtagtttttagaattaaggt | 59.01 | 116664866 | 116664896 | atgttaatttgcagcttctagaatcaaggc | 0.32 | 3 |
| Op-13 | 1R | ctaactttcaatcaatctaataaattttaactaaa | 59.24 | 116664248 | 116664279 | ctttcagtcaatctaataggttttggctgag |  |  |
| Op-13 | 2R | cctaatcatcctaattatactcctatattaaa | 58.92 | 116664308 | 116664337 | tggtcatcctgattgtactcctgtgttgag |  |  |
| Op-14 | 1F | ttagaagtattgtttgaataatgggattttt | 60.97 | 116374564 | 116374595 | ccagaagtactgtctgaataatgggatttcc | 0.40 | 3 |
| Op-14 | 1R | aattctctaactctttaatactaactcaaa | 59.26 | 116373956 | 116373986 | agttctctggctctttaatgctggctcagg |  |  |
| Op-14 | 2R | cctctttcttaacccctaaaactata | 60.02 | 116374034 | 116374064 | ggatcctctttcttgacccctagggctgtg |  |  |
| Op-15 | 1F | ggttttgagtggagttattttattttt | 59.47 | 116345331 | 116345361 | cagggttctgagtggagctattctatcttc | 0.35 | 2 |
| Op-15 | 1R | accaaacaatactaatttatcctttattaaaaa | 59.82 | 116345046 | 116345077 | cagacagtgctaatttgtcctttgttagaag |  |  |
| Op-15 | 2R | aataaaaataaatacttcccttttattttcaaa | 59 | 116345129 | 116345159 | agagatgaatgcttcccttttgttttcagg |  |  |
| Op-16 | 1F | gtagaatttttgtttggtaattttatatattgtt | 59.57 | 115798890 | 115798924 | gcagaatttctgtctggcaattttatatactgcc | 0.38 | 5 |
| Op-16 | 1R | acataaattcaaaaacaaaccatatcaata | 59.71 | 115798270 | 115798300 | acatggattcagaaacagaccatgtcagtg |  |  |
| Op-16 | 2R | atatcaataattccaaatccccataaaa | 59.74 | 115798290 | 115798319 | ccatgtcagtgattccaaatccccatgaag |  |  |
| Op-17 | 1F | cctttgaggaataataaaatggtttatagt | 60.06 | 115671053 | 115671082 | ggtttgaggaacaacaaaatggcccacagc | 0.39 | 2 |
| Op-17 | 1R | attctttactaaatttttttttcataacatctaa | 59.08 | 115670716 | 115670747 | ctttactgagtttttttttcatgacatctgg |  |  |
| Op-17 | 2R | actcatttcttatcttaccaactactaaaa | 60.95 | 115670777 | 115670806 | gctcatttcttatcttgccagctgctaagg |  |  |
| Op-18 | 1F | aagattttaataattatgagattatgatttgatt | 58.37 | 114863733 | 114863767 | aagattttaacaatcatgagactatgatttgacc | 0.39 | 2 |
| Op-18 | 1R | taaaacatcctcataaaataaactacctaa | 59.51 | 114863188 | 114863218 | taggacatcctcataggatgagctgcctgg |  |  |
| Op-18 | 2R | ctaaccaaattaataacttctcttaaaaaaa | 58.48 | 114863228 | 114863259 | ctggccaggttgatgacttctcttgaaaggg |  |  |
| Op-19 | 1F | agaaagttgattaatttttagtaatgattt | 57.78 | 114705451 | 114705480 | agaaagctgaccaacttccagcaatgactc | 0.36 | 6 |
| Op-19 | 2F | aaattaaaattaaggtgaagaatttttagt | 58.12 | 114705399 | 114705429 | aaattaaaatcaaggtgaagaactcctagc |  |  |
| Op-19 | 1R | aaaaacaaatattcttacccttctttaa | 58.57 | 114704989 | 114705017 | aaagaacaggtgttcttacccttctttgg |  |  |
| Op-20 | 1F | gagaaatataaatatgtttaaagagttatt | 55.66 | 114389180 | 114389210 | gagaaatacaaacatgttcaaagagctacc | 0.30 | 2 |
| Op-20 | 2F | tttatgtgatttattaaaagaataagtatt | 55.53 | 114389094 | 114389124 | tctatgtgattcatcaaaagaataagcatc |  |  |
| Op-20 | 1R | tctactcataaacaacaaacaa | 55.89 | 114388830 | 114388860 | acttttgctctgctcatgagcaacagacag |  |  |
| Op-21 | 1F | atgtaatgtttaggaaagttaaagataaat | 58.66 | 114336761 | 114336791 | atgcaatgcctaggaaagtcaaagacaaac | 0.38 | 4 |
| Op-21 | 2F | aaatatgtttgttgtttaaaattattggtt | 58.97 | 114336627 | 114336657 | aaacatgtttgctgctcaaaaccactggcc |  |  |
| Op-21 | 1R | aaaaacccactaatttttcaactaa | 58.18 | 114336255 | 114336281 | cagagacccactagtttttcaactgg |  |  |
| Op-22 | 1F | gtttatttttttataatgagtattataaagagattttt | 59.15 | 114192382 | 114192412 | ctttataatgagcattataaagagaccctc | 0.36 | 3 |
| Op-22 | 2F | tttttttagaagttgttagaaagtttgttt | 60.26 | 114192302 | 114192332 | tttcctcagaagctgttagaaagcctgccc |  |  |
| Op-22 | 1R | cttaaatattaataactaccatttaatcttacataaata | 58.96 | 114191876 | 114191906 | tgataactaccatttaatcttgcatggatg |  |  |
| Op-23 | 1F | atgtggttaaaattataattaattttgttatgt | 58.92 | 113946351 | 113946385 | gatgtggttaaaaccataaccaatcctgctatgc | 0.30 | 1 |
| Op-23 | 2F | ttaaagatagttatatagagtatgtttagaatattt | 58.36 | 113946208 | 113946244 | ccaaagacagttacacagagtatgtttagaacaccc |  |  |
| Op-23 | 1R | ccctcaaaatcaatctttatcaataaa | 58.7 | 113945941 | 113945968 | ccctcagaatcagtctttgtcaatagg |  |  |
| Op-24 | 1F | tgttatgtgataagaaaattggtttttttt | 60.4 | 113361481 | 113361510 | tgtcatgtgataagaaaattggtttccctc | 0.31 | 2 |
| Op-24 | 1R | aatataaatcctttacccttataaaaccttaa | 59.86 | 113360980 | 113361015 | aagtataaattccttttacccttgtaggaccttgg |  |  |
| Op-24 | 2R | aaatacaaaaatttctacctatattaaataacttaataa | 59.14 | 113361091 | 113361123 | gagatttctgcctgtgttagatgacttaatgg |  |  |
| Op-25 | 1F | gtaagttagaagggataaattttagtattttatat | 59.36 | 113106880 | 113106915 | gcaagccagaagggataaatttcagcatcctacac | 0.37 | 4 |
| Op-25 | 2F | tggttaattttggttaatttggttatttt | 59.96 | 113106842 | 113106870 | tggtcaatcctggctaatctggttatctc |  |  |
| Op-25 | 1R | acaaatacttcccttccaaaaataaaa | 60.35 | 113106365 | 113106393 | gacaaatacttcccttccaaagataagg |  |  |
