# Supplementary material for: Permissive Transcriptional Activity at the Centromere through Pockets of DNA Hypomethylation
Source: PLoS Genet. 2006 Feb 10;2(2):e17. doi: 10.1371/journal.pgen.0020017 (PMC1361766; doi:10.1371/journal.pgen.0020017)
Supplement: Supplementary file 5 [file pgen.0020017.st005.doc]

**Supplementary Table S4.** **Bisulfite Primers used in this study.**

Sequences listed here include primers used for bisulfite PCR and their corresponding sequence in the genome (Orignal Primer sequence) with Start and End co-ordinates of each primer for Human Chromosome 10 on the UCSC Genome Browser (http://genome.ucsc.edu). For each Island/Islet three primers were selected for PCR. Hemi-nested primer is denoted with a 2 prefix and used in the second round of PCR.

|  | **Size of Island/Islet** |  | **Bisulfite Sequence** | **Tm** | **Start** | **End** | **Original Primer sequence** | **Number of CG's Assayed** |
| --- | --- | --- | --- | --- | --- | --- | --- | --- |
| 1 | 70bp | 1R | ctaaactctcactacatatcaaattatactaa | 59.59 | 113126907 | 113126876 | ctgaactctcactgcatgtcaggttgtgctgg | 7 |
|  | 70bp | 2F | ggtgatattaggatttttatatggtttagt | 59.91 | 113127408 | 113127379 | ggtgacactaggattcctacatggctcagc | |
|  | 70bp | 1F | ttatattgagaagttttaggaattgttttt | 59.21 | 113127440 | 113127411 | ttatattgagaagctccaggaattgttccc |  |
| 2 | 82bp | 2R | ctatatacacaaactcccttaataacaaaaa | 60.63 | 113667828 | 113667798 | gccaaaatcactcttgtcatcaagggag | 10 |
|  | 82bp | 1R | accaaaatcactcttatcatcaaaaaaa | 60.86 | 113668002 | 113667975 | ctgtgtgcacagactcccttgatgacaagag | |
|  | 82bp | 1F | aaaaagtaaatagaattttttatagtgtaagatttt | 59.37 | 113668495 | 113668460 | aaaaagtaaatagaacccttcatagtgcaagattcc | |
| 3 | 70bp | 2F | actccttccttacaaaaacaaaa | 60.18 | 113801731 | 113801701 | gctccttccttgcagaaacagag | 7 |
|  | 70bp | 1R | catttaatcaaatcataaaataccaaacata | 59.54 | 113801782 | 113801760 | catttggtcaggtcatgggatgccaagcatg | |
|  | 70bp | 1F | tatttttttttaatataggaagattttgtgtat | 59.15 | 113802303 | 113802271 | tatttttttctaacacaggaagattctgtgcac | |
| 4 | 92bp | 2R | caatctctattaaaaataaaattccaacataata | 59.7 | 113885391 | 113885359 | cagtctctgttaggagtgaagttccaacatggtg | 10 |
|  | 92bp | 1R | taaatacaactaacacaatctctattaaaaata | 59.75 | 113885407 | 113885374 | taggtgcagctggcacagtctctgttaggagtg | |
|  | 92bp | 1F | gtgtttgatagttttagtgtagtatagtat | 60.32 | 113885968 | 113885939 | gtgtttgacagccctagtgcagcacagcac | |
| 5 | 230bp | 1R | ttttaaaaaaataactcatctctattaactaaaa | 59.31 | 114046831 | 114046798 | ttttaaaaaggtaactcatctctgttgactgggg | 19 |
|  | 230bp | 2F | tttaaatttttgattttaagtgatttgtttatt | 59.35 | 114047425 | 114047393 | ctcaaactcctgacctcaagtgatctgcccacc | |
|  | 230bp | 1F | ggttaggttggttttaaatttttgatt | 60.91 | 114047437 | 114047411 | ggtcaggctggtctcaaactcctgacc |  |
| 6 | 94bp | 1R | cccacttctttaatctaaacatacaaa | 61.02 | 114062306 | 114062280 | cccacttctttggtctagacatgcaag | 10 |
|  | 94bp | 2F | ggagaagtttttgtttattaaagtgttt | 60.87 | 114062786 | 114062759 | ggagaagcttctgtccaccaaagtgccc |  |
|  | 94bp | 1F | agtaagttggaagtgtatgttaattatt | 60.34 | 114062869 | 114062842 | agcaagctggaagtgtatgccaattacc |  |
| 7 | 54bp | 2R | aaaaataaaatttcaccatattaatcaaactaa | 59.87 | 114168333 | 114168306 | agagatggggtttcaccatgttggtcaggctgg | 12 |
|  | 54bp | 1R | actaaaattacaaacatataccaccaaa | 60.98 | 114168391 | 114168359 | gctggaattacaggcatgtgccaccagg |  |
|  | 54bp | 1F | tttatttaagataaagtgtgagttggtt | 60.93 | 114169019 | 114168992 | cttactcaagataaagtgtgagctggcc |  |
| 8 | 60bp | 2R | aattataaccaataaatttccattaaaaaa | 57.07 | 114274392 | 114274363 | gattgtagccaataggtttccattaggggg | 10 |
|  | 60bp | 1R | ctttacacaactttaatatacaaattataa | 56.92 | 114274414 | 114274385 | ctttacacaactttggtgtgcagattgtag |  |
|  | 60bp | 1F | gttgattaagattttagttttgtattaaat | 57.91 | 114274739 | 114274710 | gttgattaagactccagctttgcattaaac |  |
| 9 | 64bp | 1R | tcatactatctcctaacaaactcaata | 60.42 | 114361406 | 114361380 | tcatgctatctcctgacaggctcagtg | 9 |
|  | 64bp | 2F | tttaaagaaaaattgttttttaagatttaagatt | 59.4 | 114361870 | 114361837 | cctaaagaaaaattgcttcttaagactcaagacc | |
|  | 64bp | 1F | atatttgataatgttagatgtttgatgttt | 60.07 | 114361916 | 114361887 | acatttgataatgccagatgcctgatgctc |  |
| 10 | 174bp | 2R | ctaaaactttcaaaacaacactaaaaataa | 59.35 | 114529876 | 114529848 | ctggggctttcaaagcagcactgagagtgg | 13 |
|  | 174bp | 1R | ctatatatccaaaaccatttcactaacta | 59.62 | 114529920 | 114529891 | ctgtgtgtccagggccatttcactggctg |  |
|  | 174bp | 1F | ttgttaattataagttgttttttagttagattttat | 59.39 | 114530171 | 114530136 | ttgtcaactacaagctgctctttagccagacttcac | |
| 11 | 115bp | 2R | ctcctaaactcccaatataccataaaa | 61.64 | 114568640 | 114568620 | ctcctgggctcccagtgtgccatgggg | 22 |
|  | 115bp | 1R | cccaatcaaacacccctataa | 61.06 | 114568673 | 114568647 | cccagtcaaacacccctgtgg |  |
|  | 115bp | 1F | ggtttgggtttttggtagaatattt | 61.46 | 114569192 | 114569168 | ggcctgggtccttggcagaacattc |  |
| 12 | 81bp | 2R | cctacttccaaaactaacctaaaaa | 59.32 | 114570834 | 114570805 | cctacttccaggactggcctaggag | 17 |
|  | 81bp | 1R | aataaaactaacacttacctacaataaa | 58.77 | 114570880 | 114570856 | gagtagggctgacacttgcctgcaggtggg | |
|  | 81bp | 1F | tttatygttaaggtggttagtatagagttt | 58.71 | 114571463 | 114571434 | cccatcgccaaggtggctagcactgagctc | |
| 13 | 63bp | 1F | tcaaaatatactttaaaattccatcttaaaa | 59.82 | 114592584 | 114592552 | gctcaaaatgtactttggagttccatcttgggg | 16 |
|  | 63bp | 2F | gtgttgtttttaggatttagttaattagt | 59.2 | 114592958 | 114592930 | gtgctgcccccaggattcagccaatcagc | |
|  | 63bp | 1F | tttagttatttgtgttaatttggaatagtt | 59.22 | 114592999 | 114592970 | tccagccacctgtgccaacctggaacagcc | |
| 14 | 116bp | 1F | agttaggtattaatgaagtttaggtagttt | 60.64 | 114592972 | 114592950 | agccaggcattaatgaagtccaggcagccc | 16 |
|  | 116bp | 2F | gttaatgggagaatgtgttgttt | 60.94 | 114592958 | 114592930 | gccaatgggagaatgtgctgccc |  |
|  | 116bp | 1R | ctcaaaatatactttaaaattccatcttaaaa | 59.5 | 114592584 | 114592553 | ctcaaaatgtactttggagttccatcttgggg | |
| 15 | 64bp | 2R | tccatttaaatctcataatattaaacaaaactata | 59.48 | 114725393 | 114725359 | gtggagcttggagaaaagagacatac | 10 |
|  | 64bp | 1R | ataaataaacccaattataaaacaaacctataa | 59.5 | 114725674 | 114725645 | atggataaacccaattatggggcagacctgtag | |
|  | 64bp | 1F | ggttttttgttataaggtttagtagagttt | 60.9 | 114725765 | 114725736 | ggcctcctgttacaaggcttagcagagccc | |
| 16 | 92bp | 2R | actatatttccttccccaaataaaattatt | 60.93 | 114775309 | 114775281 | actgtatttccttccccaaatgaggttatt | 10 |
|  | 92bp | 1R | atatcccaatatttcacaaaaaactttaa | 60.12 | 114775350 | 114775321 | atatcccagtgtttcacaggagactttgg |  |
|  | 92bp | 1F | tttttatttgggaggtttgttagattattt | 61.82 | 114775820 | 114775791 | ccttcatctgggaggtctgctagaccatcc |  |
| 17 | 97bp | 2R | taactaaaacaacaaaacttacatacaa | 59.71 | 114775990 | 114775958 | tggctgaggcagcaagacttgcatgcag | 9 |
|  | 97bp | 1R | attacaacaaaataatacttaaataacaaacta | 59.11 | 114776022 | 114775995 | gttacagcaagatgatacttgagtgacaggctg | |
|  | 97bp | 1F | tatggttgggagtttttaaggt | 60.87 | 114776488 | 114776467 | catggctgggagctctcaaggc |  |
| 18 | 117bp | 1R | ccataactcaaacaatttatcaaacaataa | 61.05 | 114806819 | 114806790 | ccataactcagacaatttgtcaggcagtgg | 13 |
|  | 117bp | 2F | gttttagtttttagattgttaatttttatt | 56.78 | 114807335 | 114807306 | gtcccagttctcagactgtcaatttccact |  |
|  | 117bp | 1F | gtttagtgaataagatttttagggattgat | 61.56 | 114807377 | 114807348 | gtccagtgaacaagactcccagggactgac | |
| 19 | 97bp | 1R | aaaacaataactaaacttacttcttcaaa | 59.97 | 114808126 | 114808097 | aaaagcagtgactgagcttacttcttcagg | 13 |
|  | 97bp | 2F | ggttttgaggtagttaggatttt | 59.85 | 114808626 | 114808604 | ggttctgaggcagccaggactcc |  |
|  | 97bp | 1F | tttattatttattagttttgaagtttttaggtt | 59.42 | 114808699 | 114808667 | cttactatccaccagccctgaagtcctcaggcc | |
| 20 | 83bp | 2R | tacctcaataaaatcatattcccaatataa | 60.17 | 114843247 | 114843224 | tgcctcagtgaggtcatattcccagtgtag | 11 |
|  | 83bp | 1R | taaacactacaatacctccctaaa | 60.17 | 114843286 | 114843257 | tgggcactgcagtgcctccctggg |  |
|  | 83bp | 1F | ggggatgatatgttatatattaaaggttt | 60.44 | 114843723 | 114843695 | ggggatgacatgtcacacaccaaaggccc | |
| 21 | 56bp | 1R | actttacaacacttaatcacctttta | 60.38 | 114875326 | 114875301 | actttgcagcacttgatcaccttttg | 11 |
|  | 56bp | 2F | tgagtttttattttgataagtaaaaagtttt | 59.63 | 114875818 | 114875788 | tgagcctccactttgacaagtaaaaagcccc | |
|  | 56bp | 1F | gttattttttttatatggtggtaataaaagt | 59.44 | 114875851 | 114875821 | gccattccctttacatggtggtaacaaaagc | |
| 22 | 160bp | 2R | cctcttcaaataaaaacttactaa | 55.39 | 114913270 | 114913241 | gcccaccctcttcagatggaagcttactag | 22 |
|  | 160bp | 1R | crttcrctacatacaaaataaaaacaacta | 54.52 | 114913305 | 114913276 | cgttcgctacatacaaggtgaaggcagctg | |
|  | 160bp | 1F | taaattattaattaatgtagttatatggtat | 54.82 | 114913780 | 114913751 | taaacatcaactaatgtagccacatggcac | |
| 23 | 162bp | 1R | ccttaatatttattaaaacataaactaaacacta | 59.27 | 114973936 | 114973903 | ccttaatgtttattggaacatgggctgagcactg | 10 |
|  | 162bp | 2F | atttttatggttgttagttgttaatatttt | 59.07 | 114974505 | 114974476 | acttccatggttgttagctgccaatatccc |  |
|  | 162bp | 1F | gtttgtttgtatttgaattatttgaagat | 59.24 | 114974596 | 114974568 | gcttgcctgtacctgaattatctgaagac |  |
| 24 | 119bp | 2R | ctaacactataaaaaataaaacaattttctaataaa | 59.36 | 115046953 | 115046921 | ctggcactgtgggaaatggggcagttttctagtgag | 12 |
|  | 119bp | 1R | cctacaaaaaatacraactacaaaataatctaa | 59.05 | 115046985 | 115046950 | cctgcaaggagtgcgggctgcagggtgatctgg | |
|  | 119bp | 1F | ttgttattttgttgtttagtgttaagttt | 60.19 | 115047334 | 115047306 | ttgtcattctgctgtttagtgccaagtcc |  |
| 25 | 51bp | 2R | caaactaatactaaactatctattatctaaaataca | 59.09 | 115069430 | 115069394 | caggctggtgctgagctatctgttgtctgggatacg | 8 |
|  | 51bp | 1R | ttaaaaaacttatactattttcaatataaataccaaa | 59.14 | 115069462 | 115069427 | ttgggaggcttatactgttttcagtatggataccagg | |
|  | 51bp | 1F | gtggagtttggagaaaagagatatat | 60.59 | 115069749 | 115069724 | ttgggaggcttatactgttttcagtatggataccagg | |
| 26 | 58bp | 2R | aaacacccataaaaaacatattctaaataaaa | 60.14 | 115101941 | 115101914 | gaacacccatagaaagcatattctgaatgaag | 11 |
|  | 58bp | 1R | cctaaatcacttaacccccaaatatata | 60.71 | 115102025 | 115101994 | cctaagtcacttggcccccaagtgtgtg |  |
|  | 58bp | 1F | agtggaagaataaggttggattttt | 61.1 | 115102449 | 115102425 | agtggaagaacaaggctggatttcc |  |
| 27 | 80bp | 2R | acttaaatttaacaccaaaatatcttataataaaaa | 59.25 | 115105242 | 115105215 | acttgggtttggcaccaaaatgtcttgtggtaaagg | 17 |
|  | 80bp | 1R | ctattccctttatcacaatcactaaaaa | 60.49 | 115105307 | 115105272 | ctgttccctttatcacagtcactggagg |  |
|  | 80bp | 1F | tattttggttgtgtatagttttaggtttat | 60.11 | 115105985 | 115105956 | caccttggttgtgcacagccccaggcccac | |
| 28 | 126bp | 2R | aaaaataaaaattcaaaacaaccatcaaaa | 60.13 | 115148741 | 115148714 | ggaagtgagaattcaggacagccgtcaggg | 9 |
|  | 126bp | 1R | caaaaataaaaaaccccaaaaatcaaaa | 60.09 | 115148794 | 115148765 | cagaaatagagagccccaggaatcaaag | |
|  | 126bp | 1F | gggttattttatgttgaaggtatagt | 59.36 | 115148932 | 115148907 | gggccattttatgctgaaggcacagc |  |
| 29 | 78bp | 2R | cccccaatttaattactacaaaacaaa | 60.9 | 115243682 | 115243653 | cccccaatttgattgctacagagcagg | 15 |
|  | 78bp | 1R | aaacaaatttctattacaaaacaacactta | 59.96 | 115243714 | 115243688 | gaacaggtttctattgcaaagcaacacttg |  |
|  | 78bp | 1F | ttttttttttatggtggaggtgataatt | 60.87 | 115244267 | 115244240 | cccccttcttatggtggaggtgacaacc |  |
| 30 | 52bp | 2R | ccctactaactctaaacaacaacata | 60.48 | 115316956 | 115316925 | agctccctgctggctctagacaacagcatg | 14 |
|  | 52bp | 1R | cttaacctctcacaaaaattatactttaataa | 59.45 | 115316994 | 115316965 | cttaacctctcacaggggttgtgctttgatgg | |
|  | 52bp | 1F | gggttttttagaaaatgtttagagtttt | 59.91 | 115317515 | 115317488 | gggtctctcagaaaatgctcagagttcc |  |
| 31 | 75bp | 1R | aaaatcaacccaccaaaactacta | 60.77 | 115342155 | 115342131 | ggaggtcagcccaccagagctgctg | 16 |
|  | 75bp | 2F | ggaaagatgttgtaatggtagtttatt | 60.07 | 115342730 | 115342704 | ggaaagatgctgcaatggtagcccatc |  |
|  | 75bp | 1F | ttattggagatagagtggttttattttatt | 59.76 | 115342769 | 115342740 | tcactggagatagagtggctctatctcacc |  |
| 32 | 62bp | 2R | aaaaaaaactaaatccaacctatttaaaaaa | 59.14 | 115373413 | 115373384 | gggaaagctgggtccagcctatttagaggg | 7 |
|  | 62bp | 1R | acctaatatccaattccaaaaacaacctaa | 59.74 | 115373450 | 115373421 | gcctggtgtccagttccagaaacagcctgg | |
|  | 62bp | 1F | aatttaattttaatgttgtggggtttt | 59.48 | 115373704 | 115373677 | caacccaatcctaatgctgtggggtccc |  |
| 33 | 127bp | 2R | atatttctactacaactaacaccaa | 58.14 | 115468816 | 115468785 | gtgtttctgctgcagctggcaccag | 10 |
|  | 127bp | 1R | attcatttaaattataaccttaaaaaacatt | 57.28 | 115468853 | 115468829 | gttcatttaaattgtggccttggaaggcattg | |
|  | 127bp | 1F | tttggtataagagtagttattatagataatt | 57.25 | 115469047 | 115469017 | cttggcacaagagcagtcattatagacaatc | |
| 34 | 434bp | 2R | aaaaaataaacractaaaaacaaaacttatcaa | 57.95 | 115602293 | 115602260 | agaaggtggacggctgggagcaggacttgtcag | 32 |
|  | 434bp | 1R | aaaaacaacctaatctaccaatatctacaaaaaa | 62.8 | 115602320 | 115602288 | aaggacagcctggtctaccagtatctgcagaagg | |
|  | 434bp | 1F | tagaaagatgattagaagatattggggaatt | 62.39 | 115602706 | 115602676 | cagaaagatgaccagaagacactggggaacc | |
| 35 | 55bp | 2R | actatatctccttcaaccttctaaaaa | 60.02 | 115685411 | 115685381 | actgtgtctccttcaaccttctggagg | 7 |
|  | 55bp | 1R | cccttaactaaattattaccaactttaaaa | 59.72 | 115685443 | 115685417 | cccttaactgggttattgccagctttagagg |  |
|  | 55bp | 1F | gagaaaaaaaggtagggatagatttt | 59.51 | 115685790 | 115685765 | gagaaaaaaaggtagggacagatccc |  |
| 36 | 63bp | 1R | aactcaaaaaacttcacactaactttaa | 60.7 | 115707402 | 115707372 | agctcaggaggcttcacactggctttgg | 8 |
|  | 63bp | 1R | cctccaccaatataaaataataaatcataaaata | 59.73 | 115707454 | 115707427 | ccaccagtgtagaatggtgggtcataaggtg | |
|  | 63bp | 1F | aggggatagggttttaataggagttttt | 60.19 | 115707761 | 115707735 | agggacagggctccaacaggagccttc |  |
| 37 | 96bp | 2R | ctaattaaaccccacctaacaaaaa | 60.11 | 115725931 | 115725898 | tcttgctggttgggccccacctggcagggg | 13 |
|  | 96bp | 1R | actaactactaaatttctactcttactaattaaa | 59.71 | 115725947 | 115725918 | gctgactactaagtttctactcttgctggttggg | |
|  | 96bp | 1F | aatggttttgtatgttttatgtggtt | 60.85 | 115726343 | 115726318 | aatggctttgcatgtcccatgtggcc |  |
| 38 | 88bp | 2R | rtcaacataaactaacaatacctttttattta | 60.14 | 115789939 | 115789904 | gtcagcataaactgacagtacctttttgtttg | 19 |
|  | 88bp | 1R | actaatctcaaaattttaaaaatctaaaaacttaaa | 59.83 | 115790024 | 115789993 | actaatctcagagttttgagaatctgggaacttggg | |
|  | 88bp | 1F | tagtttagtagggtgtgtgtaaaattt | 60.84 | 115790526 | 115790500 | cagcctagcagggtgtgtgtaaaaccc |  |
| 39 | 2428bp | 2R | crcaaaaccttccaacractactctacta | 59.69 | 115792462 | 115792438 | cgcaaggccttccagcgactgctctgctg | 53 |
|  | 2428bp | 1R | ctctactaactacccttcttcctaa | 60.5 | 115792613 | 115792585 | ctctgctggctgcccttcttcctgg |  |
|  | 2428bp | 1F | gtttatttgttatyggttttaagtaaatatagattttt | 58.63 | 115792955 | 115792918 | gttcacctgctatcggtcttaagtaaacacagatctcc | |
| 40 | 121bp | 1R | aacttctacaaatcttataactctatataaataaatacaa | 59.05 | 115814221 | 115814181 | aacttctgtcaagtcttgtggctctgtataaatgggtgcag | 9 |
|  | 121bp | 2F | gtttttttggtttgtatttaagttttgttt | 60.52 | 115814641 | 115814612 | gcctccctggcctgcattcaagctctgccc | |
|  | 121bp | 1F | tgtggtttggtagaaagagtatt | 60 | 115814681 | 115814659 | tgtggtttggcagaaagagcatc |  |
| 41 | 482bp | 1R | tccRtaaaaaaaattccctatcaaaccaa | 60.31 | 115849917 | 115849889 | tccgtgagaaaaattccctatcaagccag | 42 |
|  | 482bp | 2F | gtggttatgtggttatatggttttt | 59.97 | 115850635 | 115850608 | gtggtggccatgtggccacatggctttc |  |
|  | 482bp | 1F | gatgagttattggtttttgttgttagt | 60.89 | 115850707 | 115850681 | gatgagccactggcctttgctgtcagc |  |
| 42 | 61bp | 2R | ctctatcaaaaaaaaaaccctaaacaa | 59.42 | 115850713 | 115850686 | ctctgtcagaggggagaccctgagcag | 9 |
|  | 61bp | 1R | caacaaaaaccaataactcatcaataaa | 60.02 | 115850750 | 115850724 | cagcaaaggccagtggctcatcagtgag |  |
|  | 61bp | 1F | taaggtttttggtttaagaattaagttttt | 60.81 | 115851041 | 115851012 | caaggcccctggtttaagaaccaagtcccc | |
| 43 | 72bp | 2R | aatctatttcttaaaacccaaata | 54.52 | 115879669 | 115879642 | ggtctgtttcttgaaacccaggtg | 5 |
|  | 72bp | 1R | aattcaacaatcaattattatatctaaa | 54.73 | 115879711 | 115879688 | agttcagcagtcaattgttatgtctggg |  |
|  | 72bp | 1F | attagataaatagtagttttgatattttt | 54.91 | 115879849 | 115879820 | atcagtataaatagcagttctgatatcttc |  |
| 44 | 134bp | 1F | gggtgaggaggagtaatagttaat | 61.09 | 115923168 | 115923139 | gggtgaggaggagtaatagccaac |  |
|  | 134bp | 2R | cataacraaatccaaccaaccaaaaataaa | 60.47 | 115923176 | 115923146 | catagcggggtccaaccagccaagagtggg | 30 |
|  | 134bp | 1R | taacaaaactactaaaactctaactcaaaaa | 60.58 | 115923216 | 115923187 | taacaaggctgctggagctctggctcagggg | |
| 45 | 241bp | 2R | cctacraaactaacacatcaacaaaaataa | 59.58 | 115923307 | 115923278 | cctgcggagctgacacatcaacaaagatgg | 11 |
|  | 241bp | 1R | cacrccctaacaaaactactaaaactctaa | 60.68 | 115923580 | 115923554 | cacgccctaacaaggctgctggagctctgg | |
|  | 241bp | 1F | aagaatttgattaaagggtaggagatt | 60.74 | 115923658 | 115923635 | aagaacccgaccaaagggcaggagacc | |
| 46 | 119bp | 1R | aacctataactaaaacacaaacta | 56.06 | 115928301 | 115928278 | agcctgtaactagagcacaagctg | 27 |
|  | 119bp | 2F | ggaagygttatttttaaatttagagaattt | 55.36 | 115928730 | 115928701 | ggaagcgctatttccaaactcagagaatcc | |
|  | 119bp | 1F | tgatttatatttaagtaaatgttttagtat | 55.12 | 115928916 | 115928887 | tgacccacatctaagtaaatgccctagcac | |
| 47 | 666bp | 2R | cctctcccaaaaaaaacaaacaaata | 59.16 | 115988428 | 115988398 | cgccctctcccaggagagacaaacaggtg | 53 |
|  | 666bp | 1R | rcrtaaaaataaaatacttttttatttacaaacctaaa | 59.09 | 115988540 | 115988512 | agtgaagtacttttttatttgcagacctggg |  |
|  | 666bp | 1F | ggagggtagtttgggtttaatatatgtttttt | 60.45 | 115989032 | 115989003 | agggcagcttgggtttaatacatgccctcc |  |
| 48 | 60bp | 1R | ctacactcctctattccaaaaaactata | 60.83 | 116019461 | 116019434 | ctggactcctctattccagagagctgtg | 8 |
|  | 60bp | 2F | gtaatgggtttattaaagaaaaatagagtatatt | 59.84 | 116019959 | 116019926 | gcaatgggcttatcaaagaaaaacagagcatacc | |
|  | 60bp | 1F | taagtaatgtaatttgtgttttggagt | 60.15 | 116020008 | 116019982 | taagcaatgtaacctgtgttctggagc |  |
| 49 | 200bp | 1r | accatatctaaccttatctctaactctata | 60.58 | 116034910 | 116034881 | gccatgtctggccttgtctctaactctgtg | 38 |
|  | 200bp | 2F | gtttagggatttggttgaatagattt | 60.27 | 116035593 | 116035568 | gctcagggatttggttgaacagatcc |  |
|  | 200bp | 1F | tttgggttaggtattgggattttatt | 60.71 | 116035651 | 116035626 | cctgggtcaggcactgggaccttacc |  |
| 50 | 104bp | 1R | ctcttatccaccatattcctatactaaa | 60.09 | 116053140 | 116053113 | ctcttgtccaccatattcctgtgctgag | 12 |
|  | 104bp | 2F | tattagttgggagagtttttaggttt | 60.62 | 116053523 | 116053498 | caccagctgggagagcctctaggccc |  |
|  | 104bp | 1F | gttttttgagttattggaaaagttgt | 60.05 | 116053585 | 116053560 | gttctctgagctactggaaaagctgc |  |
| 51 | 89bp | 1R | aaaccttcacttattccaaaaaata | 58.02 | 116103150 | 116103124 | aaagaccttcacttattccagggagtg | 8 |
|  | 89bp | 2R | taaaaactacaactcttaactcataaaa | 58.13 | 116103235 | 116103208 | tggaagctgcaactcttggctcatggag |  |
|  | 89bp | 1F | ttagataaaggaattatatataggttgttt | 57.71 | 116103569 | 116103540 | tcagataaaggaattacacacaggctgtcc | |
| 52 | 94bp | 1R | cttaaacacaaccccaaaacata | 60 | 116183838 | 116183816 | cttaagcacagccccaaagcatg | 9 |
|  | 94bp | 2R | ataataaaataacttaccctcctaaatataaaa | 58.6 | 116183871 | 116183839 | ataataaagtggcttaccctcctgggtgtggag | |
|  | 94bp | 1F | ttgtgttgtagtttttaaagatattgtt | 59.25 | 116184186 | 116184157 | tgctgtgttgcagcttctaaagacattgtc |  |
| 53 | 113bp | 1R | aaacttactttatctaacacaaacatttaa | 59.39 | 116236935 | 116236906 | aggcttactttgtctaacacaagcatttgg | 8 |
|  | 113bp | 2F | tttagtagtgtattttgtttttttaggtttt | 60.17 | 116237211 | 116237181 | cttagtagtgcattctgtcttctcaggctcc |  |
|  | 113bp | 1F | gttatttttagaagtgttgtttagaggat | 60.75 | 116237266 | 116237237 | gccattctcagaagtgctgtctcagaggac | |
| 54 | 73bp | 1R | aaaaaatcccctcttatttatttctaaaaa | 59.43 | 116294117 | 116294088 | aaagggtcccctcttgtttgtttctgaggg | 14 |
|  | 73bp | 2F | ttaggttttttgttagttagttttgagat | 60.6 | 116294556 | 116294528 | ccaggctttctgccagttagctctgagac |  |
|  | 73bp | 1F | tgggtttagtgggtttagtatttt | 60.56 | 116294615 | 116294592 | tgggctcagtgggctcagtactcc |  |
| 55 | 76bp | 1R | tttaaaaaacacattacaaaaaaataatctaaataa | 58.97 | 116407015 | 116406980 | tttgaagaacacattacagaagaatgatctggatag | 8 |
|  | 76bp | 2F | aatagagagaattgtggggtagt | 60.69 | 116407408 | 116407379 | ccaggggaacagagagaattgtggggcagc | |
|  | 76bp | 1F | ggtgttttaagtttttgagggtaattat | 60.91 | 116407495 | 116407468 | ggtgccctaagctcttgagggtaatcac |  |
| 56 | 734bp | 1F | ttagagagttggagagttattyggagtattt | 60 | 116518210 | 116518240 | ccagagagctggagagccattcggagcaccc | 50 |
|  | 734bp | 2F | tagatttttygtttttyggggatagagttt | 59.8 | 116518132 | 116518522 | cagacccctcgttcctcggggacagagccc |  |
|  | 734bp | 1R | actcacctctaacraacataatacaaacaa | 60.1 | 116517804 | 116517833 | actcacctctggcggacatggtgcaggcag |  |
| 57 | 790bp | 1R | tccaaatcrtcccraaaaaaactactacaa | 59.87 | 116571188 | 116571159 | tccaggtcgtcccgggagaggctgctgcag | 22 |
|  | 790bp | 2F | gtttagggygttygtttggggttttatttt | 60.61 | 116571395 | 116571366 | gcccagggcgcccgcctggggcctcacccc | |
|  | 790bp | 1F | gagggtagggattgtyggtttggggtagtt | 69.68 | 116571490 | 116571461 | gagggcagggactgccggcctggggcagcc | |
| 58 | 59bp | 1R | caaaacctaaccattaaatcatacttatattta | 59.8 | 116631047 | 116631015 | caagacctggccattaaatcatacttgtgtttg | 10 |
|  | 59bp | 2F | gagtaatagttattatttagttgaggttgt | 60.03 | 116631306 | 116631277 | gagcaacagttaccacccagctgaggctgc | |
|  | 59bp | 1F | gttttaggtggttgttgaagtttt | 60.97 | 116631346 | 116631323 | gccttaggtggctgctgaagctcc |  |
| 59 | 413bp | 1R | accataaatcttcacaaacctcaa | 60.83 | 116687088 | 116687065 | gccatggatcttcacaagcctcag | 18 |
|  | 413bp | 2F | ggttgtagtatttgttagtagtttttttt | 59.56 | 116687600 | 116687572 | ggctgcagtacctgccagcagcttctcc |  |
|  | 413bp | 1F | ttgagaattataattgttaagtaggttata | 57.46 | 116687801 | 116687772 | ttgagaactacaactgccaagtaggctac |  |
| 60 | 90bp | 1R | aataaatttaactcaatataaaaataaactccaa | 58.96 | 116743717 | 116743684 | ggtggatttagctcagtgtaaggatgaactccag | 7 |
|  | 90bp | 2F | ggaatgatatttagttgtgttttagaat | 59.04 | 116743930 | 116743903 | ggaatgacatccagttgtgcttcagaac |  |
|  | 90bp | 1F | gggtataagggatggagtttt | 59.5 | 116743976 | 116743956 | gggcacaagggatggagtccc |  |
| 61 | 81bp | 1R | cccrtaaaactacatattcttcaatataatata | 56.78 | 116839913 | 116839881 | cccgtggggctgcatgttcttcagtgtaatgtg | 10 |
|  | 81bp | 2R | ttattatactttaaattctaaaatacatatacaa | 56.27 | 116840003 | 116839974 | tatactttaagttctgggatacatgtgcag |  |
|  | 81bp | 1F | ttatttttttttatttgttaagttaaagttagt | 57.47 | 116840412 | 116840383 | attcttctccatctgccagccaaagtcagc |  |
| 62 | 1190bp A | 1R | accacacctaaaaaacctatctccaatcaa | 66.44 | 116842030 | 116842001 | ctcaaaatgtactttggagttccatcttgggg | 41 |
|  | 1190bp A | 2F | ttgygttyggygtttttgaggggatttgat | 60.34 | 116842452 | 116842423 | ctgcgcccggcgctcctgaggggacctgac | |
|  | 1190bp | 1R | rgctaaaaacaaaatcraaaaactaaataa | 56.69 | 116842543 | 116842514 | cgctaaggacaaggtcgggagactgggtgg | 64 |
| 63 | 1190bp A | 1F | gtatygttatttagtttttygattttgttt | 56.69 | 116842549 | 116842520 | gcatcgccacccagtctcccgaccttgtcc | |
|  | 1190bp | 2F | tatagtagttagttgttttygtttagtagtt | 57.43 | 116842885 | 116842855 | cacagcagccagctgttcccgtccagcagcc | |
|  | 1190bp | 1F | ggaagttatagtatagtagttagttgtttt | 59.37 | 116842896 | 116842867 | ggaagccatagcacagcagccagctgttcc | |
| 64 | 114bp | 2R | atttatcctacaacrtatccaaatatatatacaaaaa | 59.21 | 116877373 | 116877338 | atttatcctacaacgtatccaagtatgtatgcaaggg | 13 |
|  | 114bp | 1R | aaaataataaaaaatactttcacaaaaataactaaaa | 59.19 | 116877525 | 116877489 | aagtaatgagagatgctttcacaggaatggctagag | |
|  | 114bp | 1F | ttggttgtataatataaaagagtatgttattattt | 59.31 | 116877932 | 116877898 | ttggttgtataatataaaagagcatgctatcaccc | |
| 65 | 78bp | 1R | tcccaaataactaaaactacaaacatata | 59.44 | 117052426 | 117052398 | tcccaagtagctgggactacaggcatgtg | 6 |
|  | 78bp | 2F | aagaaaaaaaaatttaaggagttttgtttt | 59.62 | 117052765 | 117052738 | gaaaaaaaaatccaaggagtcttgctcc |  |
|  | 78bp | 1F | gtaattatagtttttttggaatttggattaaatat | 59.66 | 117052863 | 117052829 | gtaactacagtttctttggaacctggatcaaacac | |
| 66 | 254bp | 1R | tctctattacccaacctaaaa | 55.67 | 117072878 | 117072858 | tctctgttgcccagcctggag | 16 |
|  | 254bp | 2F | tatygattttggtttgttaaattttgat | 55.56 | 117073281 | 117073254 | taccgaccttggtttgccaaaccctgac |  |
|  | 254bp | 1F | gataaaatattgtttaygtaaatttgtagtatatt | 55.64 | 117073326 | 117073292 | gataaaacattgtttacgtaaatctgcagcacacc | |
| 67 | 103bp | 1R | tttattccctacaactctctaccttata | 60.82 | 117128776 | 117128749 | tttattccctgcagctctctgccttgtg | 14 |
|  | 103bp | 2F | gtattagaaaggtttttaagaatttgtttttttat | 60.21 | 117129253 | 117129219 | gtactagaaaggtttctaagaacctgtttttctac | |
|  | 103bp | 1F | gaaatattgtagaatattaagttttttgttaattgt | 60 | 117129368 | 117129333 | gaaatactgtagaacactaagtttcttgctaattgc | |
| 68 | 69bp | 1R | attaccaaactttcttaattactataattttaaaa | 58.99 | 117135025 | 117134991 | attaccaaactttcttaattgctatggttttaaag | 10 |
|  | 69bp | 2R | acacatcttaaaattatatcttttaaatcttcta | 59.4 | 117135058 | 117135025 | gcacatcttgaaattatgtcttttaagtcttctg | |
|  | 69bp | 1F | tttttaattggattggtaaagtagaatatt | 59.08 | 117135506 | 117135477 | ccctcaactggattggcaaagcagaatatc | |
| 69 | 200bp | 1R | ctttatttacctaaacaaacctaaacaataa | 59.72 | 117285370 | 117285340 | ctttgtttacctaagcaagcctgggcaatgg | 23 |
|  | 200bp | 2F | gtagagattttgtgattaaattaggtttatt | 59.43 | 117285931 | 117285901 | gtagagatcctgtgactaaattaggttcacc | |
|  | 200bp | 1F | agatttttttgtatttgtaattttttatagtgttt | 60 | 117286022 | 117285988 | agacttttttgtatttgtaattcttcacagtgtcc | |
| 70 | 61bp | 1R | aaaattttacaactttacaacacaaaa | 58.58 | 117579990 | 117579964 | aaagttttacagctttacaacacagag | 8 |
|  | 61bp | 2R | ccttacctttttatcccttaactataaa | 59.45 | 117580022 | 117579995 | ccttgcctttttatcccttggctgtgag |  |
|  | 61bp | 1F | ttatttataatgtatttatttatgagatgtttatttttt | 58.31 | 117580508 | 117580470 | ctcatccatatgcactcatctatgagatgctcattcctc | |
| 71 | 73bp | 2R | acaaaaaatacacaatccaaaataa | 56.77 | 117644270 | 117644241 | acaagaggtacacagtccaaggtgg | 7 |
|  | 73bp | 1R | acactaataatcaaatatttacataatcta | 56.06 | 117644302 | 117644278 | gcactaataatcagatatttgcatgatctg |  |
|  | 73bp | 1F | tttttataaagttggaaataatgtatttat | 55.92 | 117644503 | 117644474 | cttccacaaagctggaaataatgcattcac | |
| 72 | 58bp | 1R | aacaacaaaaccattatcaatata | 55.4 | 117717066 | 117717044 | acagcaaaaccattatcagtgtg | 6 |
|  | 58bp | 2R | atatccattctattattaataaactctatta | 55.68 | 117717096 | 117717066 | gtatccattctgttgttaatgggctctgttg |  |
|  | 58bp | 1F | ggtattttttaatttatatttagtaagtt | 54.3 | 117717257 | 117717229 | ggcactttttaatccatacttagcaagcc |  |
| 73 | 90bp | 1R | ccaatcaaccttatcaactacataaaaa | 60.78 | 117756073 | 117756046 | ccagtcagccttatcaactgcatagaag | 7 |
|  | 90bp | 2R | ttcaaacaaactcctaacttaaattaataa | 59.19 | 117756170 | 117756141 | ttcagacaagctcctgacttgaattggtgg |  |
|  | 90bp | 1F | aatattatgagtttaagagttgtttgattatt | 59.46 | 117756506 | 117756475 | aacaccatgagtctaagagctgcttgatcacc | |
| 74 | 90bp | 1R | ctacataaataaatataaactaaatattatcactcaaa | 59.07 | 117869834 | 117869797 | ctgcataggtgagtataaactaggtgttgtcactcagg | 8 |
|  | 90bp | 2R | aaatacaacaactctactacacatacta | 60.3 | 117869869 | 117869842 | ggatgcagcagctctgctgcacgtgctg |  |
|  | 90bp | 1F | gagygttggtttaggtgttataaatatgtttt | 60 | 117870185 | 117870154 | gagcgctggctcaggtgccacaaatatgttcc | |
| 75 | 52bp | 1R | aaattaactcttctactccactttaaaaaa | 59.33 | 117900957 | 117900928 | gagttgactcttctactccactttagggag | 7 |
|  | 52bp | 2F | tagatggatatagtgtgtatgtgttttt | 60.84 | 117901398 | 117901371 | cagatggatacagtgtgcatgtgctttc |  |
|  | 52bp | 1F | aagttttttgagtttagttttaggtatttt | 59.93 | 117901446 | 117901417 | aagcttcctgagcccagcctcaggtacttc | |
| 76 | 53bp | 1R | aattacraaaacacaaacaatcccttatta | 59.33 | 117975107 | 117975077 | gattacggaggcacaaacagtcccttgtttg | 8 |
|  | 53bp | 2R | ctctaccaactaaatccttaaactaaaa | 59.83 | 117975140 | 117975113 | ctctgccaactggatccttgaactaggg |  |
|  | 53bp | 1F | gaagtaaaagtttttttgaattatatagagtttat | 59.42 | 117975442 | 117975408 | gaagcaaaagcccctctgaaccatacagagctcac | |
| 77 | 68bp | 1R | cttatttacttctatatctcraacttttctaaaaaaa | 60.17 | 118014516 | 118014480 | cctgtttacttctgtgtctcgaacttttctgaggaag | 12 |
|  | 68bp | 2F | gtggagggttgttttttagaaatt | 60.55 | 118014859 | 118014836 | gtggagggctgctttccagaaacc |  |
|  | 68bp | 1F | aattaagtagttaagttaggagagaattgt | 60.98 | 118014898 | 118014869 | aaccaagtagccaagtcaggagagaactgc | |
| 78 | 396bp | 2R | actacatttaaacccraccaaaacctaa | 61.32 | 118022727 | 118022698 | tgcatttaaacccggccagacctgg | 22 |
|  | 396bp | 1R | ccccacrtcactaataactataattcaaaacttaata | 62.4 | 118022924 | 118022900 | tcactagtggctgtgattcaagacttggtg |  |
|  | 396bp | 1F | ygattaaaatgagttatttttgtttgttttttt | 60.41 | 118023316 | 118023287 | atcaaaatgagccattcttgtctgtcctcc |  |
| 79 | 118bp | 1R | aaattaaaaacaaatctatctaaaaataattctaaaaa | 59.14 | 118073811 | 118073774 | gggttggagacaggtctatctgggaatggttctagaag | 10 |
|  | 118bp | 2R | tattctactttccaaaatcctcaaaattaa | 60.53 | 118073854 | 118073825 | tattctgctttccaggatcctcagagttgg |  |
|  | 118bp | 1F | gttttttttttagtatggtatgtaagtttttt | 59.89 | 118074112 | 118074081 | gtctttccctcagcatggcatgcaagctcccc | |
| 80 | 87bp | 2R | caaaaaaattccaaaaattcaaaa | 55 | 118228045 | 118228016 | caaaggaattccaaaagttcaagg | 7 |
|  | 87bp | 1R | ataataatacataaactatctaaacaaaaa | 55.12 | 118228063 | 118228040 | ataatagtgcatgaactatctaggcaaagg | |
|  | 87bp | 1F | taggatagtgttgattataatatatattatatt | 55.54 | 118228549 | 118228517 | caggacagtgctgatcacaatatacatcatatc | |
| 81 | 313bp | 2R | ccrcctataatcccaacactttaaataa | 58.4 | 118273012 | 118272983 | ccgcctgtaatcccagcactttgggagg | 18 |
|  | 313bp | 1R | aaataatttcacttcaaaaaataacaaaaa | 57.85 | 118273061 | 118273033 | aggtgatttcacttcagaaaatggcagggg | |
|  | 313bp | 1F | ggtttatgtatgggatgagatttttttgtt | 63.11 | 118273358 | 118273329 | ggtctatgtatgggatgagactcccctgcc |  |
| 82 | 116bp | 1R | caaaaaaaataatcctttcttcaaaaatatcaaa | 60.56 | 118298143 | 118298110 | caaagggagtagtcctttcttcagaggtgtcaag | 12 |
|  | 116bp | 2F | aaattgtatgggtttaggaggatt | 60.15 | 118298488 | 118298465 | aaactgtatgggcttaggaggacc |  |
|  | 116bp | 1F | gttgatttattttgtttttgttttgagtt | 59.92 | 118298530 | 118298502 | gttgactcattctgcccttgctttgagcc |  |
| 83 | 70bp | 1R | acccaaataaataataaactataccatctaaa | 60.93 | 118302550 | 118302519 | gcccaggtaggtagtaggctatgccatctagg | 9 |
|  | 70bp | 2F | aaggtaaaaygtagggttgtattaatttttatt | 59.39 | 118303015 | 118302982 | aaggtaaaacgtagggttgcaccaaatttccatc | |
|  | 70bp | 1F | ttttagaatagygatttttgattatggggttt | 59.94 | 118303064 | 118303033 | cctcagaacagcgatttctgatcatggggccc | |
| 84 | 79bp | 1R | aaaaattccraaatctaaatccttcaaaactta | 60.08 | 118306898 | 118306866 | gaaaattccgggatctgaatccttcagggcttg | 13 |
|  | 79bp | 2F | taattttagtattttgggaggttaaggt | 60.93 | 118307426 | 118307399 | taatcccagcactttgggaggccaaggc |  |
|  | 79bp | 1F | ttggttgtgtatggtggtttatatt | 60.92 | 118307452 | 118307429 | tggctgtgcatggtggctcacacc |  |
| 85 | 149bp | 2R | aataaaaccccaactatatttacatcaaaa | 60.56 | 118321038 | 118321009 | gatagaaccccaactgtgtttgcatcaggg | 17 |
|  | 149bp | 1R | tttctaacacacaaaacacaaaaaaaaata | 60.89 | 118321172 | 118321143 | tttctggcacacaaggcacaaggaagagtg | |
|  | 79bp | 2R | aataatcaactaaatacrtcatattcctaataaaatatta | 58.57 | 118321245 | 118321210 | ggtggtcagctaaatgcgtcatgttcctaataaaatgttgaaag | 14 |
| 86 | 79bp | 1R | attctaaatctcaaaaaataacractaaataatcaa | 59 | 118321280 | 118321237 | attctaaatctcagagaataacggctgggtggtcag | |
|  | 149bp | 1F | tagaatttttgttttttgagttgaggt | 60.73 | 118321649 | 118321623 | cagaacttttgtttcctgagctgaggc |  |
|  | 79bp | 1F | tagaatttttgttttttgagttgaggt | 60.97 | 118321649 | 118321623 | cagaacttttgtttcctgagctgaggc |  |
| 87 | 190bp | 1R | ccataaatataattaataacaaaaccattttcaa | 59.8 | 118332040 | 118332007 | ccataggtatgattaatgacaaagccattttcag | 15 |
|  | 190bp | 2F | gatttaataaagtttattgatgatgtttaaagt | 59.35 | 118332401 | 118332369 | gacccaacaaagcttattgatgatgcccaaagc | |
|  | 190bp | 1F | tttagttgtttgaattaaaataaataaataagtagt | 59.39 | 118332448 | 118332411 | cctagttgcttgaatcaaaacaaacaaacaagcagcac | |
| 88 | 142bpp | 2R | aaactaaatataaattcaatcaaaatattccaatta | 59.51 | 118337465 | 118337432 | aaactggatgtaagttcaatcaaaatattccagttg | 13 |
|  | 142bpp | 1R | tttatatacctataaatccttacttataacccta | 60.1 | 118337485 | 118337452 | tttgtatgcctatgaatccttacttgtgaccctg | |
|  | 142bpp | 1F | aaagatttttgggtgagttttttaaataat | 60.43 | 118337499 | 118337466 | aaagatttctgggtgagtctttcaaacaac |  |
| 89 | 142bp | 2R | aatccttacttataaccctaaaactaaatataaa | 60 | 118337521 | 118337486 | aatccttacttgtgaccctgaaactggatgtaag | 10 |
|  | 142bp | 1R | ttcaaaaaaaaatccacaaatttatatacctata | 60.09 | 118337802 | 118337776 | ttcaaaaagagatccacagatttgtatgcctatg | |
|  | 142bp | 1F | gatggagttttgttttgttatttaggt | 60.89 | 118337893 | 118337864 | gatggagtcttgctctgtcacccaggc |  |
| 90 | 51bp | 1R | ctatcatatttccaaacttataaaaaacactt | 60.11 | 118339792 | 118339761 | ctgtcatatttccagacttgtgggaagacttg | 8 |
|  | 51bp | 2F | ggttgagagtttaagtttaagtttttttt | 60.54 | 118340213 | 118340186 | ggctgagagcttaagtttaagcccttcc |  |
|  | 51bp | 1F | tgggagttttgttatagttaaagttttt | 60.57 | 118340250 | 118340223 | tgggagccctgccacagttaaagctttc |  |
| 91 | 50bp | 2R | ccataaaaaataacccaataaaaacaaaaa | 59.83 | 118350771 | 118350750 | ccataggaggtgacccagtaaagacagagg | 18 |
|  | 50bp | 1R | tataccaacctaccccataacaa | 60.98 | 118350803 | 118350774 | gtaccaacctgccccatgacag |  |
|  | 50bp | 1F | ttgttgaaatattggtaagtattttatgtatataat | 59.54 | 118351390 | 118351351 | ttgttgaaatattggcaagtatttcatgcatcacaacatc | |
| 92 | 104bp | 1R | tccataaaaccttatttaatttataaattaacaaataa | 59.43 | 118352577 | 118352540 | tccataaggccttgtttgatttgtagattaacaaatag | 7 |
|  | 104bp | 2F | tttttttattttgaggtaataaagtataagaaattt | 59.72 | 118352940 | 118352905 | ttctcttactctgaggcaacaaagcataagaaattc | |
|  | 104bp | 1F | tataggagaggaagagtttaatttatttatat | 59.47 | 118353008 | 118352978 | cacaggagatggaagagttcaatttattcac | |
| 93 | 67bp | 2R | aaatacatctatatactctaaatctatctccta | 59.56 | 118358062 | 118358031 | ggtgcatctgtatgctctgggtctatctcctg | 24 |
|  | 67bp | 1R | ccaaatacaacttctataacraaaacacaata | 59.97 | 118358177 | 118358146 | ccaggtacaacttctgtagcgaagacacagtg | |
|  | 67bp | 1F | aatttatttttttagagaggttggtttttt | 60.38 | 118358583 | 118358554 | aatctacctcctcagagaggctggtcctcc | |
| 94 | 141bp | 2R | cccctcrttaaccctaactcttaaaa | 60.41 | 118358587 | 118358556 | cccctcgttggccctggctcttgggg | 24 |
|  | 141bp | 1R | aaaccaacctctctaaaaaaaataaatttaaa | 60.63 | 118358623 | 118358598 | aggaccagcctctctgaggaggtagatttgag | |
|  | 141bp | 1F | gggagtggtgatttgggatat | 62 | 118358916 | 118358896 | gggagtggtgacttgggacac |  |
| 95 | 544bp | 2R | cctcaacctcccraataactaaaactacaa | 63.22 | 118359049 | 118359020 | cctcagcctcccgagtagctgggactacag | 30 |
|  | 544bp | 1R | crctctatcrcccaaactaaaatacaataa | 55.78 | 118359133 | 118359104 | cgctctgtcgcccaggctggagtgcagtgg | |
|  | 544bp | 1F | gttattatttaaggggtgggtgtttttggt | 66.01 | 118359525 | 118359496 | gccaccacccaaggggtgggtgtccctggc | |
| 96 | 102bp | 1R | caaatcaaaatacaatctatacaactcaa | 59.53 | 118369022 | 118368994 | caggtcaaggtacagtctgtgcagctcag | 11 |
|  | 102bp | 2F | ttattttttttattagagagtttagatagaggat | 60.15 | 118369467 | 118369434 | ctaccttccctattagagagtctagacagaggac | |
|  | 102bp | 1F | gaattgattgtttttgaagatagatattttatttat | 59.93 | 118369527 | 118369492 | gaactgactgtttttgaagacagacaccttacttac | |
| 97 | 51bp | 1R | atatccaaaaaaattttattccaaaaatactata | 59 | 118369645 | 118369616 | ccaaagggattttgttccagagatgctatg | 9 |
|  | 51bp | 2F | gggttttattgtggtggtttttt | 61.02 | 118370072 | 118370044 | gactcaggggctccactgtggtggcttcc |  |
|  | 51bp | 1F | taggagttagatagtagtatattaggattt | 59.97 | 118370104 | 118370075 | caggagttagacagcagcacactaggaccc | |
| 98 | 102bp | 1R | aatttaaaaatacattccaaaatctcacaa | 60.28 | 118373959 | 118373930 | aatttagaagtgcattccaaagtctcacag | 14 |
|  | 102bp | 2R | atttactcccacacttaaacttctaa | 60.62 | 118373999 | 118373967 | gaactgaatttgctcccacacttaagcttctgg | |
|  | 102bp | 1F | agttttaatgtttataaagttgggttagat | 60.53 | 118374844 | 118374815 | agcttcaatgcccacaaagctgggtcagac | |
| 99 | 71bp | 1R | gttatttatcttccccaaaaaatatttaaaataa | 59.58 | 118375862 | 118375827 | cccttgtttgtcttccccagaaaatgtttgaagtgg | 9 |
|  | 71bp | 2F | ttttttttgattgtaatttgggttattagt | 60.23 | 118376124 | 118376095 | cccttcttgattgcaatctgggctactagc |  |
|  | 71bp | 1F | taaagaggaaggttgtagagttttatt | 60.65 | 118376161 | 118376135 | caaagaggaaggttgcagagcttcatc |  |
| 100 | 89bp | 1R | ccaataataaacaccaatataaacacaaaa | 60.38 | 118381972 | 118381943 | ccagtgatgagcaccagtgtgggcacaggg | 9 |
|  | 89bp | 2F | ggagaaatgtatagtgattttggtatatt | 60.88 | 118382139 | 118382110 | tggagaaatgcacagtgaccctggcacacc | |
|  | 89bp | 1F | tttttttggttatttggtagaaaatttttt | 59.68 | 118382170 | 118382141 | tttctctggctacttggtagaaaacttccc |  |
| 101 | 53bp | 2R | aaaatcaaaaaacctaattaataaaaaaaaccta | 59.93 | 118382250 | 118382218 | gaatcaaaaagcctggttgatgggg | 6 |
|  | 53bp | 1R | aaaatacaataaaaaaataaccaaaaaccataa | 59.93 | 118382325 | 118382301 | aaaatgcagtaagagagtggccagaggccatgg | |
|  | 53bp | 1F | gtttagttaggtgttatatgtttaaagtttt | 59.93 | 118382547 | 118382517 | gtctagccaggtgtcacatgttcaaagcccc | |
| 102 | 50bp | 1F | ggttttagatgtgtttttttggttt | 59.74 | 118414271 | 118414295 | ggtttcagatgtgccttcttggccc | 5 |
|  | 50bp | 2R | atacaaatatataaaaaaacctctccaaaaa | 59.33 | 118414417 | 118414447 | atgcaaatatgtaaagaaacctctccaggag |  |
|  | 50bp | 1R | aacattttttaattactttctctataccataata | 59.20 | 118414460 | 118414493 | aacattttttagttgctttctctgtgccatggtg |  |
| 103 | 63bp | 1R | acctttacatttaatactaacccctaaa | 60.74 | 118414613 | 118414583 | ggagcctttgcatttggtgctgacccctggg | 9 |
|  | 63bp | 2F | ttgtgtgaaaattttttaggagaaaatat | 59.78 | 118414884 | 118414856 | ctgtgtgaaaaccttccaggagaaaacac | |
|  | 63bp | 1F | gaatgtttaaggaggatgatatgaatt | 59.99 | 118414920 | 118414886 | ctgccaaggaatgtccaaggaggatgacatgaacc | |
| 104 | 61bp | 2R | taacacctctcataaccttaaa | 59.82 | 118434038 | 118434009 | caggtccctggcacctctcatggccttgag | 9 |
|  | 61bp | 1R | aaaaatactcaaaaaaacaacataactaaa | 58.68 | 118434076 | 118434047 | gaggatgctcagagaaacagcatggctggg | |
|  | 61bp | 1F | tagtaattggattttttaagtttttttgtt | 58.68 | 118434408 | 118434379 | cagtaattggacctcccaagcccctctgcc | |
| 105 | 111bp | 1R | aaaaacaaaaattttcaaaaacaacaataa | 58.98 | 118448132 | 118448106 | gagcaaaggtttcaggggcaacagtag | 8 |
|  | 111bp | 2F | gaaagtagtgatgttgggtttt | 59.11 | 118448390 | 118448369 | gaaagcagtgatgctgggctcc |  |
|  | 111bp | 1F | tattttttttttgggattatatttagtagt | 59.91 | 118448444 | 118448415 | catcctctctctgggaccacactcagcagc | |
| 106 | 98bp | 1R | acatttctaacccctacaaataaa | 58.22 | 118457045 | 118457022 | gcatttctagcccctgcagatggg | 14 |
|  | 98bp | 2F | tgttttggaaaattatttatttttttttagt | 57.95 | 118457444 | 118457414 | tgccttggaaaattacccacttcttcctagc |  |
|  | 98bp | 1F | gatattttaaattttttggtttttttgattat | 57.43 | 118457482 | 118457451 | gacacctcaaaccttctggtttccttgaccac | |
| 107 | 1143bp | 2R | taaaaatcaccraataaccaaacctaaaa | 61.98 | 118491890 | 118491864 | tggaagtcaccgaatggccaggcctggag | 24 |
|  | 1143bp | 1R | rcraaaaactacctaactccraaacta | 62.24 | 118492087 | 118492059 | gcggggaactgcctggctccggagctg |  |
|  | 1143bp | 1F | gygatttagttttyggggtaagtatttttt | 64.82 | 118492353 | 118492324 | gcgactcagcctccggggcaagcacctccc | |
| 108 | 78bp | 1R | ataaatttaaaaactttcaaaattaaaaattaaaaa | 57.32 | 118514826 | 118514791 | gtaggtttgagaactttcaggattaaaagttgaagg | 8 |
|  | 78bp | 2F | agggtagttatttatgattatgtatttagtat | 59.01 | 118515316 | 118515287 | agggcagctactcatgatcacactcagcac | |
|  | 78bp | 1F | tttagtgttagggtagttatttatgattat | 58.95 | 118515325 | 118515296 | cccagtgtcagggcagctactcatgatcac | |
| 109 | 78bp | 2R | taaaccccactatatcctaaaaacaaa | 60.68 | 118549520 | 118549492 | tagaccccactgtgtcctgagagcagg | 12 |
|  | 78bp | 1R | aacaaatcacaaatacctaccataaaaaa | 60.55 | 118549584 | 118549558 | agcaagtcacaggtgcctaccatgaaagg | |
|  | 78bp | 1F | tagataaaaagatagagaattagagtaaattgt | 59.55 | 118550135 | 118550103 | cagacaaaaagacagagaatcagagcaaattgc | |
| 110 | 449bp | 1R | ccccaacaaaataaacctacctaa | 60.9 | 118598204 | 118598181 | ccccaacagagtgggcctgcctgg | 38 |
|  | 449bp | 2F | ttttttttatggtaggagatttaaggttat | 60.11 | 118598694 | 118598665 | cttcctccatggtaggagatttaaggctac |  |
|  | 449bp | 1F | gtttttgtagttttttagtggttttatagt | 60.01 | 118598739 | 118598710 | gcttctgcagctccctagtggtcccacagc | |
| 111 | 99bp | 2R | actaaattcatttaatttcctctttctaaaaa | 59.28 | 118605516 | 118605487 | gctggattcatttaatttcctctttctgggag | 10 |
|  | 99bp | 1R | atattctcattatccccttaaacttaataa | 59 | 118605570 | 118605539 | atattctcattgtccccttgaaattggtgg |  |
|  | 99bp | 1F | ttagagggtgttattttttggagt | 60.28 | 118605773 | 118605750 | tcagagggtgccatcccctggagc |  |
| 112 | 88bp | 2R | acactataacaatttcctttatctttaaaaaatta | 60.25 | 118628860 | 118628833 | acactgtgacagtttcctttgtctttaaagaattg | 7 |
|  | 88bp | 1R | ttacctttcaacactctcaacttataaa | 61.07 | 118628947 | 118628913 | ttgcctttcagcactctcagcttgtagg |  |
|  | 88bp | 1F | tatggttaagtggaataaagtaagaaagt | 60.94 | 118629163 | 118629134 | catggctaagtggaaacaaagcaagaaagc | |
| 113 | 58bp | 2R | cccaaccaacttcracttaactatacta | 58.47 | 118645575 | 118645539 | cccaaccagcttcggcttgactgtactg | 6 |
|  | 58bp | 1R | aaaatatataaatataaaactaatcaacctaactaaa | 58.37 | 118645624 | 118645597 | agaatgtgtaaatgtgggactagtcaacctagctggg | |
|  | 58bp | 1F | gagaaaataaattgggtagttttttttatt | 58.83 | 118645947 | 118645918 | gagaaaataaattgggcagtctttcccacc | |
| 114 | 943bp | 2R | ccrcttctccaacaaaaaaaataacactata | 60.53 | 118753677 | 118753643 | cccgcttctccggcaaggaaggtggcactgtg | 37 |
|  | 943bp | 1R | aacaaatatataaaaataaaaccaaactaactaaa | 59.33 | 118753740 | 118753709 | aacaaatgtgtaagggtggggccaagctagctgag | |
|  | 943bp | 1F | ttgaaggagtaaggtaggtagt | 60.58 | 118754063 | 118754042 | ctgaaggagcaaggtaggcagc |  |
| 115 | 58bp | 1R | acataatcatatacctcaaaatcaaaaaaaa | 59.77 | 118843920 | 118843890 | acataatcatgtgcctcaaaatcaagggagg | 7 |
|  | 58bp | 2F | tagtagtatagtggatgtattttgttaagt | 60.68 | 118844201 | 118844172 | tagtagcatagtggatgcaccctgctaagc | |
|  | 58bp | 1F | tattgataggttattttgttggttattgtt | 60.55 | 118844235 | 118844206 | cattgacaggttattttgttggtcactgcc |  |
| 116 | 307bp | 1F | gaygttgaggtgaggttgtttagt | 60.55 | 118913088 | 118913111 | gacgttgaggtgaggttgcccagc | 26 |
|  | 307bp | 1R | ctacaaaaatactaaaaaacaaccaacta | 60.01 | 118912090 | 118912119 | ctgcagaggtgctaagaaaacagccagctg |  |
|  | 307bp | 2R | acacacraatttaaacaatactcacattttaaa | 60.47 | 118912218 | 118912250 | gcacacgggtttgggcagtgctcacattttggg |  |
| 117 | 154bp | 2R | ataccrctacttccctcaatacaaaaaa | 59.97 | 118874939 | 118874910 | cccatgccgctgcttccctcagtgcaagaag | 25 |
|  | 154bp | 1R | tcaataaaataaactaataacctaactttctaa | 59.07 | 118875084 | 118875054 | gtaaaatgggctgatgacctgactttctgg |  |
|  | 154bp | 1F | gagttttttttttgtgagtttggatt | 60.5 | 118875434 | 118875405 | cctggagctctcctcctgtgagcctggacc | |
| 118 | 165bp | 2R | cccctctacaaaataacaaattcaaaaa | 61.57 | 118924949 | 118924919 | gtcccctctgcaaggtggcagattcagaag | 38 |
|  | 165bp | 1R | aaaaaaatccttaaccctttcaaaaataaaa | 60.99 | 118924992 | 118924963 | ggaaagatccttggccctttcagagatgggg | |
|  | 165bp | 1F | tgtttgygtggtgatatgagtatttgtt | 60.94 | 118925546 | 118925519 | tgcctgcgtggtgacatgagcacctgtc |  |
| 119 | 376bp | 2R | ccccacaacctccctacaaa | 60.92 | 118966863 | 118966834 | ccccacagcctccctgcggg | 26 |
|  | 376bp | 1R | rcaacatacccactaacttcaccctaacta | 60.61 | 118966895 | 118966876 | gcagcatgcccactagcttcaccctggctg | |
|  | 376bp | 1F | tgttagtgttttagaaggataggttatatt | 60.61 | 118967388 | 118967359 | tgccagtgccccagaaggacaggccacacc | |
| 120 | 937bp | 1F | gggttttagyggatgttatagtttttgatt | 59.40 | 118991521 | 118991550 | gggtcttatcggatgtcacagtttttgacc | 43 |
|  | 937bp | 2F | tgtyggggtaggggtgttt | 59.95 | 118991436 | 188991455 | tgccggggcaggggtgccc |  |
|  | 937bp | 1R | ataaaaattctaaattaaatctccaaattaaa | 59.10 | 118990940 | 118990970 | tgggggttctaggttgggtctccagattggg |  |
| 121 | 50bp | 1R | cctcacacctactttataatttatctataaaaa | 60 | 119039935 | 119039903 | cctcacacctactttgtagtttatctgtaaagg | 6 |
|  | 50bp | 2F | taaaaggaaatttataaagtgttggatttatat | 59.5 | 119040117 | 119040085 | taaaaggaaatttacaaagtgttggacttacac | |
|  | 50bp | 1F | gtatttagttatggtgttttttgtatataatagtt | 59.65 | 119040232 | 119040199 | gcatcagccatggtgctctctgcacataacagtc | |
| 122 | 69bp | 1R | ctccaactcacctaatcttataattcrataaaa | 60.32 | 119124123 | 119124091 | ctccagctcacctgatcttgtaattcggtaggg | 36 |
|  | 69bp | 2F | ttgttggaggggttgagtt | 61.31 | 119124560 | 119124541 | cctgctggaggggctgagcc |  |
|  | 69bp | 1F | ggagttygaggagttgttgtagatt | 60.4 | 119124599 | 119124575 | ggagttcgaggagctgctgcagacc |  |
| 123 | 165bp | 1R | cattccaaatacctaaaactctaacta | 60.14 | 119246314 | 119246288 | cattccaagtgcctaggactctggctg | 19 |
|  | 165bp | 2F | ttttagtttgggtagtaagagtaaaattt | 60.9 | 119246879 | 119246851 | ctccagcctgggcagcaagagcaaaactc | |
|  | 165bp | 1F | gtagtgagtgagttaatattagattattgt | 60.36 | 119246906 | 119246881 | gcagtgagccaacatcagaccactgc |  |
| 124 | 84bp | 2R | cctacaaaatactaaccaatacttttaaa | 59.72 | 119304602 | 119304574 | cctgcaaggtactgaccagtgcttttggg | 17 |
|  | 84bp | 1R | actatattctaatcacttatcaactaaaa | 59.76 | 119304634 | 119304606 | gctgtgttctggtcacttgtcaactgagg |  |
|  | 84bp | 1F | ggygtttatatttttttgaagagtagagttt | 59.5 | 119305254 | 119305225 | ggcgtccacattctctgaagagcagagccc | |
| 125 | 71bp | 2R | aaactaatcttaccaaaacctttaaaaaaa | 60.81 | 119411460 | 119411434 | gggctaatcttaccagaacctttagagggg | 15 |
|  | 71bp | 1R | ctctcctccatttaataaataaacaaa | 59.2 | 119411495 | 119411466 | ctctcctccatttaataggtaggcagg |  |
|  | 71bp | 1F | ttgaattttaatygaagattttatttgaaagtttt | 59.41 | 119411929 | 119411895 | ctgaattttaatcgaagatttcatctgaaagtccc | |
| 126 | 91bp | 1F | ttgatttttagagtagttttaattagatgtatt | 59.92 | 119581210 | 119581178 | ctgattcccagagcagctctaatcagatgcacc | 21 |
|  | 91bp | 1R | acraaaaatcaatctttaatcaaccaaacta | 60.52 | 119580684 | 119580654 | gcggggagtcaatctttgatcagccaagctg | |
|  | 91bp | 2R | atctttaatcaaccaaactaaaataaatcta | 59.82 | 119580673 | 119580643 | atctttgatcagccaagctgaaataggtctg | |
| 127 | 54bp | 1R | ctttcctaaataattcatataacactctaaa | 59.54 | 119758531 | 119758501 | ctttcctggatggttcatatggcactctgag | 7 |
|  | 54bp | 2F | tgtgaattgttatatttattttaagtaaggt | 59.82 | 119758849 | 119758819 | tgtgaactgtcacattcactttaagcaaggc | |
|  | 54bp | 1F | gtttttgttttaggattggttagtt | 59.86 | 119758878 | 119758854 | gttcttgcctcaggactggctagcc |  |
| 128 | 74bp | 2R | cctaacaaaaacaaatccataaataatattaa | 59.55 | 119796720 | 119796692 | tggcaggagcaggtccataaatggtgttgg | 20 |
|  | 74bp | 1R | tatatcctaaatacccaatacctaacaaa | 60.96 | 119796743 | 119796714 | tgtgtcctggatgcccagtgcctggcagg |  |
|  | 74bp | 1F | ggattgtatgatagataaggtagtttt | 59.81 | 119797339 | 119797313 | ggactgcatgatagacaaggcagtccc |  |
| 129 | 61bp | 1R | tatccctctctactaaaaccattttaa | 60.81 | 119882180 | 119882154 | tgtccctctctgctggggccattttgg | 10 |
|  | 61bp | 2F | gaggaataatggatataaaatttttatatatgtt | 59.08 | 119882782 | 119882749 | gaggaacaatggacacaaaattcccacacatgcc | |
|  | 61bp | 1F | gtttagttttttgtatgggatttgt | 59.92 | 119882815 | 119882791 | gctcagcctcttgcatgggacttgc |  |
|  |  |  |  |  |  |  |  |  |
|  |  |  |  |  |  |  | Total CG’s Assayed | 2028 |
|  |  |  |  |  |  |  |  |  |
