# Supplementary material for: Permissive Transcriptional Activity at the Centromere through Pockets of DNA Hypomethylation
Source: PLoS Genet. 2006 Feb 10;2(2):e17. doi: 10.1371/journal.pgen.0020017 (PMC1361766; doi:10.1371/journal.pgen.0020017)
Supplement: Supplementary file 6 [file pgen.0020017.st006.doc]

**Supplementary Table S5. Table of mean raw fluorescence intensity of proteins measured at the 10q25 neocentromere.**

| **Experiment:** | **Antiserum/Antibody:** | **Fluorescence Intensity (n=60)^a^** | | | |
| --- | --- | --- | --- | --- | --- |
|  |  | Control | | 20μM 5-aza-dC | |
|  |  | Mean | SEM^b^ | Mean | SEM^b^ |
| CENP-A | CREST-3 | 8370.54 | 790.36 | 14776.50 | 2045.42 |
|  | Anti-CENP-A | 6709.17 | 1179.13 | 12229 | 1808.20 |
| Methylated DNA | CREST-3 | 23786.73 | 1198.30 | 25864.77 | 1082.41 |
|  | Anti-5mC | 59891.37 | 3016.03 | 34643.48 | 2174.11 |
| MBD1 Protein | CREST-3 | 13049.26 | 1521.18 | 17268.84 | 1338.96 |
|  | Anti-MBD1 | 143588.30 | 12286.09 | 177030.40 | 11841.14 |
| Tri-methylated lysine 9 Histone H3 | CREST-3 | 17021.16 | 1032.40 | 26333.08 | 2190.85 |
|  | Anti-3MeK9H3 | 87023.99 | 6425.80 | 90932.96 | 11704.94 |
| Penta-acetylated Histone H4 | CREST-3 | 18281.05 | 9544.66 | 21654.34 | 2197.90 |
|  | Anti-5AcH4 | 55548.26 | 5582.44 | 54919.58 | 5663.59 |

^a^ Arbitrary units as measured using IP-Lab v3.6.5a (Scanalytics Inc.).

^b^ Standard error of the mean.
