# Supplementary material for: Permissive Transcriptional Activity at the Centromere through Pockets of DNA Hypomethylation
Source: PLoS Genet. 2006 Feb 10;2(2):e17. doi: 10.1371/journal.pgen.0020017 (PMC1361766; doi:10.1371/journal.pgen.0020017)
Supplement: Supplementary file 7 [file pgen.0020017.st007.doc]

**Supplementary Table S6. Table of fluorescence intensity ratios of chromatin proteins at the 10q25 neocentromere before and after 5-aza-dC treatment.**

| **Chromatin Protein** | **Fluorescence intensity ratio** | | | |
| --- | --- | --- | --- | --- |
|  | Control | | 20μM 5-aza-dC | |
|  | Mean | SEM | Mean | SEM |
| Anti CENP-A | 0.78 | 0.16 | 0.90 | 0.19 |
| Anti 5mC | 3.12 | 0.27 | 1.63 | 0.25 |
| Anti MBD1 | 22.85 | 5.75 | 12.56 | 1.54 |
| Anti 3MeK9H3 | 5.93 | 0.92 | 4.41 | 1.31 |
| Anti 5AcH4 | 3.58 | 0.96 | 3.00 | 0.57 |
