# Supplementary material for: Permissive Transcriptional Activity at the Centromere through Pockets of DNA Hypomethylation
Source: PLoS Genet. 2006 Feb 10;2(2):e17. doi: 10.1371/journal.pgen.0020017 (PMC1361766; doi:10.1371/journal.pgen.0020017)
Supplement: Supplementary file 8 [file pgen.0020017.st008.doc]

**Supplementary Table S7. Bisulfite PCR primers used for control regions in this study.**

Table listing the primers used for bisulfite PCR and sequencing of CpG islands and CpG islets found in the control regions used in this study. The Tm of bisulfite primers were calculated using PerlPrimer ([http://perlprimer.sourceforge.net](http://perlprimer.sourceforge.net/)). The unconverted primer sequences are also shown with the number of CpG dinucleotides assayed for each primer set. The start and end coordinates correspond to the hg17 May 2004 assembly of the human genome sequence.

|  | **Size of Island/Islet** |  | **Bisulfite Sequence** | **Tm** | **Start** | **End** | **Original Primer Sequence** | **CG** |
| --- | --- | --- | --- | --- | --- | --- | --- | --- |
| 10p14 – 1 | 59bp | 1F | taggggtttaatgtttattaagttaggtat | 60.61 | 11629210 | 11629239 | caggggctcaatgtccactaagtcaggcac | 5 |
| 10p14 – 1 | 59bp | 2F | ttggagtgttttaagtatatttttttaagt | 59.17 | 11629256 | 11629282 | gagtgttctaagcatattttctcaagc |  |
| 10p14 – 1 | 59bp | 1R | aataattttaaaaataaattctaaatcccaactata | 58.95 | 11629414 | 11629441 | tgagaatgggttctggatcccaactgtg |  |
| 10p14 – 2 | 211bp | 1F | tttaggattttagatatttttggagttttt | 59.21 | 10731670 | 10731699 | cccaggaccccagatatctctggagttccc | 16 |
| 10p14 – 2 | 211bp | 2F | aaaatattggtttttggagatagatatttt | 59.11 | 10731701 | 10731729 | aaatactggtttctggagacagacatccc |  |
| 10p14 – 2 | 211bp | 1R | cccaaatcaatactcataataatttcaataa | 59.53 | 10732201 | 10732230 | agtcaatgctcatgataatttcaatggggg |  |
| 10p14 – 3 | 99bp | 1F | ttttggygtttgggtggttttaaatttat | 60.55 | 11099576 | 11099605 | gctttggcgcctgggtggccctaaacctac | 65 |
| 10p14 – 3 | 99bp | 2F | ggtttaggggattttgagaagtt | 60.81 | 11099686 | 11099709 | tcggggcccaggggaccctgagaagtc |  |
| 10p14 – 3 | 99bp | 1R | tccraaaaaaaatccaacttaaaaacaaaaa | 59.59 | 11100063 | 11100093 | tccgggaggaaatccagcttgaaggcagaag |  |
| 10p14 – 4 | 1115bp | 1F | gttagggataaaggttaaataagttttt | 58.61 | 11254452 | 11254479 | gccagggacaaaggttaaataagcctcc | 9 |
| 10p14 – 4 | 1115bp | 2F | gaagaaggaatttttttaaatgttttatattt | 58.71 | 11254495 | 11254523 | gaaggaatccctttaaatgccccatactc |  |
| 10p14 – 4 | 1115bp | 1R | cattaattccaaaaaaacaaaactactaaaa | 59.63 | 11254809 | 11254837 | ttaattccagaaaagcaaagctactgggg |  |
| 10q26 – 1 | 1112bp | 1F | tgtttttttggtgtaggtttttattaaattt | 60.77 | 124629212 | 124629241 | gtttctctggtgcaggttctcaccaaatcc | 52 |
| 10q26 – 1 | 1112bp | 2F | ggtygatatttaagtattggaagagaggtt | 61.11 | 124629310 | 124629339 | ggccgacacttaagcactggaagagaggcc |  |
| 10q26 – 1 | 1112bp | 1R | ttaaataaacraactaactttaataacaacccta | 59.15 | 124629785 | 124629814 | atgggcggactgactttgatagcagccctg |  |
| 10q26 – 2 | 77bp | 2R | acctctttttaaaacatatataatacaaaa | 57.17 | 125619020 | 125619045 | agaggaaaggagagtgacaatcatcc | 8 |
| 10q26 – 2 | 77bp | 1F | aggaaaggagagtgataattattt | 51.52 | 125619022 | 125619045 | aggaaaggagagtgacaatcatcc |  |
| 10q26 – 2 | 77bp | 1R | caatttattcaaaaactactatacataaataa | 56.77 | 125619259 | 125619288 | tgatgaggactaatgaatctgtgccagagg |  |
| 10q26 – 3 | 123bp | 1F | atttttagattgattttttaagagagttt | 57.6 | 125615647 | 125615675 | accctcagactgattccctaagagagccc | 15 |
| 10q26 – 3 | 123bp | 2F | tatattttagttatgaggtgttataggt | 57.91 | 125615708 | 125615737 | cccacacttcagctatgaggtgtcacaggc |  |
| 10q26 – 3 | 123bp | 1R | ctaataaaaacctccaaaattctaaaa | 57.17 | 125616143 | 125616171 | gcctgatgggggcctccagggttctgagg |  |
| 10q26 – 4 | 62bp | 1F | gtagttaaagtgtttgtgtgataatttt | 59.55 | 125813856 | 125813884 | tgcagtcaaagtgcctgtgtgatggcctc | 9 |
| 10q26 – 4 | 62bp | 2F | ttatttttgtaaaggtatagtagtggtt | 59.11 | 125813904 | 125813931 | ccatccttgcaaaggtacagcagtggcc |  |
| 10q26 – 4 | 62bp | 1R | aaaaattaaatattcccaaatacaaccaa | 59.6 | 125814108 | 125814137 | ggggattgaatgttcccaggtacagcccag |  |
| 10q26 – 5 | 83bp | 1F | gttttgtttggagttaaatagtatgttatt | 59.72 | 126189899 | 126189926 | gtctgcctggagtcaaatagtatgtcacc | 11 |
| 10q26 – 5 | 83bp | 2R | ataaccaaaaaatcatcaaaaaccattaaa | 60.45 | 126190293 | 126190322 | ataaccaaggaatcatcaggagccattgag |  |
| 10q26 – 5 | 83bp | 1R | caaacaaaccttcaaataaccaaaa | 59.74 | 126190313 | 126190336 | aaacaggccttcagataaccaagg |  |
